# Google Scholar as replacement for systematic literature searches: good relative recall and precision are not enough

**DOI:** 10.1186/1471-2288-13-131

**Published:** 2013-10-26

**Authors:** Martin Boeker, Werner Vach, Edith Motschall

**Affiliations:** 1 Department of Medical Biometry and Medical Informatics, University Medical Center Freiburg, Stefan-Meier-Str. 26, Freiburg i. Br. 79104, Germany

**Keywords:** Literature search, Literature search, Methods, Literature search, Systematic, Information storage and retrieval, Google Scholar, Medline

## Abstract

**Background:**

Recent research indicates a high recall in Google Scholar searches for systematic reviews. These reports raised high expectations of Google Scholar as a unified and easy to use search interface. However, studies on the coverage of Google Scholar rarely used the search interface in a realistic approach but instead merely checked for the existence of gold standard references. In addition, the severe limitations of the Google Search interface must be taken into consideration when comparing with professional literature retrieval tools.

The objectives of this work are to measure the relative recall and precision of searches with Google Scholar under conditions which are derived from structured search procedures conventional in scientific literature retrieval; and to provide an overview of current advantages and disadvantages of the Google Scholar search interface in scientific literature retrieval.

**Methods:**

General and MEDLINE-specific search strategies were retrieved from 14 Cochrane systematic reviews. Cochrane systematic review search strategies were translated to Google Scholar search expression as good as possible under consideration of the original search semantics. The references of the included studies from the Cochrane reviews were checked for their inclusion in the result sets of the Google Scholar searches. Relative recall and precision were calculated.

**Results:**

We investigated Cochrane reviews with a number of included references between 11 and 70 with a total of 396 references. The Google Scholar searches resulted in sets between 4,320 and 67,800 and a total of 291,190 hits. The relative recall of the Google Scholar searches had a minimum of 76.2% and a maximum of 100% (7 searches). The precision of the Google Scholar searches had a minimum of 0.05% and a maximum of 0.92%. The overall relative recall for all searches was 92.9%, the overall precision was 0.13%.

**Conclusion:**

The reported relative recall must be interpreted with care. It is a *quality indicator* of Google Scholar confined to an experimental setting which is unavailable in systematic retrieval due to the severe limitations of the Google Scholar search interface. Currently, Google Scholar does not provide necessary elements for systematic scientific literature retrieval such as tools for incremental query optimization, export of a large number of references, a visual search builder or a history function. Google Scholar is not ready as a professional searching tool for tasks where structured retrieval methodology is necessary.

## Background

For many scientists, especially in the life-science domains, the literature retrieval process is tedious, error-prone and nontransparent [1-4]. On the other hand, the quality of literature search has been recognized as one of the key features for the generation of high quality scientific evidence [5,6]. Between ever-growing requirements for scientific literature retrieval and a variety of different literature resources, the scientists as users of literature are asking for a unified, easy-to-use and reliable entry point to scientific information.

In the light of this well justified demand, recent results on the quality of Google Scholar as a resource for scientific literature retrieval, even for systematic reviews, gave rise to high expectations on the emergence of such a unifying search interface [7-9]. A recent paper of Gehanno et al. [9] provided compelling results on the coverage of Google Scholar for studies included in Cochrane and JAMA systematic reviews. However, the paper was received with some critique on its overly positive interpretation and conclusion which may not be justified by its methods [10-13].

Many information specialists seem to be overwhelmed by the fast evolution of databases with a multitude of technical features. In a continuing discussion and agreement process, the information science and the scientific literature retrieval communities have developed methods and standards which aim for a high level of quality in literature retrieval and reporting [14-18]. Many information scientists plead for a sound validation of Google Scholar and other developing tools before advertising them. Keeping this in mind, the publication of results on new technology could be perceived as potentially dangerous, since it may raise high expectations *without being interpretated with care in the context of literature retrieval as a scientific method*. As a result, many unexperienced users of literature retrieval methodology could tend to switch to a new and putatively better technology - and forget about the methods which have been established in recent years to increase the quality of literature searches and their reporting.

Taking the critique of their study seriously, we reinvestigated the work of Gehanno et al. [9]. However, we did not only search for the existence of references in Google Scholar, but searched with realistic search expressions in the Google Scholar search interface prior to the evaluation of the result set. To estimate the quality of the search result, we calculated the *relative recall* and precision. In this approach, the references of the included studies from the Cochrane systematic reviews are used as an alternative gold standard [19,20].

The objectives of this study were to investigate (1) searches with Google Scholar under conditions which are derived from state-of-the-art structured search procedures common to scientific literature retrieval, (2) to compare the relative recall and precision of these searches with prior results, and (3) to give the reader an overview on current advantages and disadvantages of Google Scholar.

Methodically, we analyzed the given MEDLINE search strategies of 14 Cochrane reviews and tried to translate them with the limited capabilities of the Google Scholar search interface. The evaluation of our retrieval results relies on the concept of relative recall based on the studies included in systematic reviews [19,20].

### Is Google Scholar ready to be used alone for systematic reviews?

The recent study of Gehanno et al. [9] is titled “Is the coverage of Google Scholar enough to be used for systematic reviews?”. However, the authors came to a conclusion beyond their title which we want to challenge here.

In their investigation, the authors included 14 Cochrane reviews [21-34] and 15 reviews published in JAMA. The authors measured the coverage of Google Scholar by *directly* searching for the titles of the included studies one by one. Hence, they did not estimate a recall based on a prior search strategy. The authors simply measured the coverage of Google Scholar based on the immediate verification that a reference could be found with the Google Scholar search interface. The main result was that the coverage of Google Scholar is 100% for the 738 included studies [9]. This work answers an important question on the coverage of Google Scholar, however, the authors' conclusions raise very high expectations on the actual quality of Google Scholar searches. The authors conclude their work with the following paragraph: 

“In conclusion, the coverage of GS is much higher than previously thought for high quality studies. GS is highly sensitive, easy to search and could be the first choice for systematic reviews or meta-analysis. It could even be used alone. It just requires some improvement in the advanced search features to improve its precision and to become the leading bibliographic database in medicine.”

It is highly questionable, if the coverage results alone can justify these conclusions. At least, the investigation of Gehanno et al. proves the existence of the references in Google Scholar at the point in time of their searches. As such, the first sentence of the conclusion is correct as a summary of their results on high coverage; nevertheless, their own results and the results from the literature do not sufficiently justify the second part of their conclusions.

Therefore, we have to carefully distinguish the following prerequisites related to scientific literature retrieval with Google Scholar:

1. As stated, Google Scholar has a very high *coverage* for certain topics in clinical medicine [9], although these results were not observed after structured search procedures as customary in scientific literature retrieval. Several reports from different research areas underpin these high *coverage* results [35,36]. It is at least an important precondition for the applicability of Google Scholar as a search engine for systematic reviews. Yet, these results can not be generalized to structured search procedures and all subject areas of biomedical science.

2. On the other hand, it is known that the high recall retrieval results from Google Scholar have limited precision [35]. Some authors already related these issues to the limited capabilities of the Google Search interface [13]. Currently, it is not well understood to which degree it is possible to optimize Google Scholar searches both for recall and precision.

3. A further important question is, how Google Scholar integrates with current professional conduct in scientific literature retrieval. For professional work in all domains, scientific search interfaces have certain characteristics and provide at least the following integrated tools:

– reliability and stability of search results over time and place

– export functions for search result sets

– a history function which temporarily stores retrieval results for incremental refinement of search strategies

– support for search strategy documentation

– advanced user interfaces supporting the composition of complex search expressions

### Google Scholar: a search engine for scientific literature with known limitations

Google Scholar uses technology of the Google search engine. As such it is not a literature database in the traditional sense like MEDLINE, Embase or the Web of Knowledge. In a more traditional scientific literature database, the entries for a reference database are collected from selected scientific journals, books and other resources which fulfill certain quality criteria. Information on references are extracted and stored in a separate database, e.g. the MEDLINE database. On top of this, the collected information is automatically indexed and partly processed by humans.

In Google Scholar an automated software program called a crawler visits accessible scholarly documents on the internet and builds a full-text index by storing the words extracted from the full-text together with a link to the source document. However, the reference information itself is not accessible via an additional Google Scholar reference database. Hence, the Google Scholar indexes can only contain references which are accessible via the internet in any form e.g. as full-text, via a publisher’s web-page or as a citation from the full-text of a citing work. Therefore, it can not be guaranteed that all references accessible at a given point in time are retrievable at all later points in time. Search results will change over time when indexing changes due to accessibility of source documents or databases.

The Google Scholar indexing engine implements some natural language processing algorithms to process the words collected from the sources. Further, Google Scholar automatically extracts the citation information from the references. This technology known as autonomous citation indexing, is also applied to the Web of Knowledge and Scopus, however, with differing results [37,38]. To provide the user with a meaningful sorting of references, the Google search engine technology uses ranking algorithms which do not only analyze the matching between the search expression and the full-text. References are also ranked based on how often they are cited by other references and other information [39]. By its sheer size and technological power, Google and Google Scholar are able to index everything which is accessible via the internet, store it in large distributed databases and deliver results in milliseconds.

The distinction between “scientific literature database” and “scientific search engine” should not be taken too literally, at least not technologically: on the one hand, a competitive literature database uses high end natural language processing and indexing technology to process its entries and internet technology to deliver the results; on the other hand, every index generated by a crawler is stored in databases and might be enhanced by semantic technology. Thus in the future, the discrimination between “literature database” and “scientific search engine” will be blurred. A “scientific literature database” (e.g. MEDLINE) is accessible for users only by a “search engine” with its user interface (e.g. PubMed or OvidSP). Google Scholar is a scholarly web search engine which does not provide an own resource of reference information to the users. The Google Scholar search engine and user interface directly links from its index on the documents in the web.

The documentation of Google Scholar [40] itself makes no claims on the applicability of Google Scholar in certain contexts. It states nothing about completeness of coverage or the quality of retrieval results. The user is provided with the service “as is”, as clearly stated in the legal disclaimer of Google. Referring to their own official statements, Google Scholar tries to cooperate with publishers and producers of scientific texts and provides help on how to prepare documents for indexing by Google Scholar. However, when resources are closed, access is restricted e.g. by password protection, or when the owner of the resource does not want to cooperate, Google cannot process the respective documents. Thus, Google Scholar is dependent on the fundamental accessibility of scientific texts over the internet or the will of the publishers and libraries to cooperate and open their repositories for indexing.

One of the main objectives of Google is easy access and easy usability. This policy might be appropriate for many uses, but restricts the users to a simple search interface which is not sufficient to express more complex queries. Google Scholar follows the main Google interface with the easiest possible way of interaction: a single text entry field (called the “simple search interface” hereafter). In addition, an “advanced search interface” is available. This interface allows to connect search terms with logical operators or use exact phrases in search expressions (see below).

When compared with professional literature search interfaces (PubMed, OvidSP, Web of Knowledge) independently from the underlying data sources, Google Scholar has some major limitations:

• Search fields of the simple and advanced search interfaces are limited to expressions not exceeding a length of 256 characters. This factor severely deteriorates the applicability of Google Scholar as it limits the overall expressivity of searches to very short expressions. In addition, when not carefully checked that the complete intended expression is used, the search interface truncates the expression after 256 characters *without warning* and might leave a short meaningless phrase or term that increases the number of false positive results.

• Not more than 1000 results of the complete result set can be displayed in steps of maximum 20 results per page. No bulk export of results is available [40]. Results can only be exported into reference management software (e.g. ZOTERO) by the maximum number of references per page (20). With this boundary Google Scholar can not be integrated into a professional process of reference selection for systematic reviews [41].

• Google Scholar has no truncation operators. In Google Scholar search expressions, complete words have to be used. An automatic stemming mechanism is used to detect a common word stem, however, this mechanism does not work reliably. E.g., it is not enough to search for “child” to find the terms “child”, “childhood” and “children”, the same applies to “random” for “randomisation”, “randomization”, “randomized” and “randomised”.

• Logical operators can be used, though only without nesting of logical subexpressions deeper than one level. It is possible to use conjunctions of terms, phrases and subexpression connected with the logical AND. Google Scholar uses a space ‘ ’ to express the logical AND. Subexpression are disjunctions of terms and phrases connected with the logical OR and have to be enclosed in parentheses ( … ) on one level (see for an example below). This feature is not documented.

• Although the Google Scholar search interface has been improved for correct interpretation of logical connectors [42], the retrieval results are still not stable against the variation of the search term sequence of otherwise logical equivalent search expressions. The results set of a search with the expression oesophagus OR esophagus has a size of 545,000. The logical equivalent search esophagus OR oesophagus has a size of 565,000 references.

• It is not achievable to construct all possible expressions in the *advanced search interface* due to the limited number of available entry fields. Only one field for each type of expression (conjunction, disjunction and conjunction of phrases) is available, which is not sufficient to construct e.g. a simple conjunction of two disjunctions:

(hemorrhage OR bleeding) AND (esophagus OR oesophagus)

• Such search expressions with more than one subexpression have to be constructed in a text editor outside of the Google Search interface. After construction, they have to be copied and pasted as a whole into the single entry field of the *simple search interface*. In addition, the advanced search interface parses more complex expressions into its fields although the limited number of fields is not sufficient to cover the meaning of the search expression (example above). Hence, the advanced search interface might distort a query to an expression with a completely different semantic. A complex search inserted into the simple search interface should therefore never be dispatched from the advanced search interface.

• The currentness of Google Scholar may not be very high for some resources. The update period for certain resources is up to nine months [40]. Although research results indicate very high coverage of Google Scholar, the exact coverage is not known. Google itself states that it does not index journals, only articles, and does not claim to be exhaustive.

• Literature which is not available in digital form is not reliably searchable. Only references to citations of this literature may be found and are consequently only searchable by title-words and authors.

• Some fields of the advanced search interface are not available in a search expression as a keyword or field indicator. Whereas authors can be specifically searched for with the field indicator ‘author’ in an expression like "author: author name", the date is not accessible by a field indicator.

## Methods

### Development of Google Scholar search expressions

We retrieved the newest versions of the 14 Cochrane reviews from the Database of Systematic Reviews Issue 3/2013 used in [9] from the Cochrane Library [21-34,43]. We extracted the references for the included studies from the Cochrane reviews and listed them in a separate file.

Where available, the documented search strategies for MEDLINE (OvidSP or PubMed) searches were extracted from the Cochrane reviews. Otherwise the documented general search strategy was taken. We analyzed the search strategies for their constituting blocks and aspects [44]. Phrases and terms from the text-word search part of the strategies were retrieved.

We developed initial search phrases for Google Scholar searches under the Google Scholar search interface restrictions:

• Search expressions were limited to a length of 230 characters due to the restriction of a total of 256 characters and the need for further specification of the search expression in the evaluation phase (see below).

• The general structure of the search expression was the simple conjunction of *terms*, *phrases* or *subexpressions* connected with the Boolean AND. In Google Scholar the AND is expressed as a Space ‘ ’ between terms, phrases or subexpressions.

• *Terms* in Google Scholar are complete single words (truncation is not possible). Google Scholar applies automatic stemming to *terms* where the stem is recognizable for Google Scholar. However, this mechanism might not be reliable for domain specific language (e.g. the medical language).

• *Phrases* in Google Scholar are one or more terms separated by Space enclosed in quotation marks '"'. These (connected) phrases are searched by Google Scholar exactly as they are provided to the search interface.

• *Subexpressions* in Google Scholar are disjunctions of terms and phrases connected with a Boolean OR. In Google Scholar the OR is expressed as an “OR” between parts of the search. The subexpression has to be enclosed in a pair of parenthesis ‘( … )’.

No further restrictions were applied to the Google Scholar searches. We intended to keep as much of the structure and “meaning” of the original searches in the derived searches. For each aspect (block) of the original search we initially introduced one term, phrase or subexpression in the Google Scholar search. Due to the restrictions of the Google Scholar search expression, the semantics of the MEDLINE search expression could only be transferred with a trade-off.

Generally, the searches were optimized for larger recall than precision. At the beginning of the evaluation phase some searches were optimized for higher recall by experimentally including different terms in the disjunctive subexpressions. This study was not intended to show how Google Scholar searches could be optimized for *precision*, but how Google Scholar would perform under the assumption of real world systematic review retrieval. Under these premises, we developed a “short” translation of the given professional searches developed by the information specialists for the Cochrane reviews with relatively *low effort* (not more than about 30 min per Google Scholar search expression).

All original search expression from the Cochrane reviews and our derived Google Scholar search expressions are included in Additional file 1. To illustrate Google Scholar search expressions, two examples of Google Scholar searches are presented here.

We took care *not to use the graphical user interface* of Google Scholar’s advanced search interface for dispatching the search expressions. The advanced user interface groups the terms and phrases of the subexpression “as close as possible” in the limited set of input fields (for each type of query one field, also see above). Thus, an expression can result with other semantics than the original expression. The search query was developed in a text editor and copy-pasted from there into the simple search interface of Google Scholar.

The length of MEDLINE search expressions from the Cochrane review and the derived search expressions including whitespace were measured with a text editor and documented near the searches (see Additional file 1).

### Evaluation of search results

We followed Sampson et al. using the references of the included studies from the Cochrane systematic reviews as an alternative gold standard [19,20]. We checked the occurrence of each of these references in the search results of the corresponding Google Scholar search. We included all major references that were cited where more than one reference was given and did not prioritize certain study reporting types. References were counted only once if a cited reference was repeated for more than one included study. Therefore, the overall count of included *references* might be slightly lower than the documented number of included studies in the Cochrane review.

To verify the occurrence of a reference in the result sets of Google Scholar searches, in most cases it was sufficient to insert an author expression of the study at the beginning of the search expression as a further conjunction, e.g.:

For some studies these augmented searches resulted in large result sets and further authors or parts of the title were introduced as additional search terms into the original search expression. Care was taken to limit the complete search expression to 256 characters. The presence of the references was checked by verifying the *exact match* in the Google Scholar result set. References for which the presence in the result set could be verified were marked in the list of included references (see additional electronic material, Additional file 1).

The *precision* of the search was calculated as the ratio of the number of the found included references and the number of all found references. The *relative recall* of the search was calculated as the ratio of the number of the found included references and all included references. The recall derived in this way was termed *relative recall* clearly indicating that this value might not reflect the true recall due to the limitation of the gold standard [19].

Where documented properly, we extracted the number of references found with the original searches from the Cochrane reviews. We calculated the precision of the original Cochrane review searches for the number of included references as defined above. In addition, the number of databases searched was extracted. A detailed table with commentary on these values from the Cochrane reviews is provided in the additional electronic material, Additional file 2.

## Results

In this study, we examined the result sets of Google Scholar searches (GS searches) for the occurrence of references included in fourteen Cochrane reviews as a gold standard. The Google Scholar searches were translated based on the professional searches documented in the Cochrane reviews.

We investigated Cochrane reviews with a number of included references between 11 and 70 references with a total of 396 references. The GS searches resulted in sets between 4,320 and 67,800 references and a total of 291,190 references. The relative recall of the GS searches had a minimum of 76.2% and a maximum of 100%. For 7 GS searches the maximum relative recall of 100% was measured. The precision of the GS searches had a minimum of 0.05% and a maximum of 0.92%. The overall relative recall for all GS searches was 92.9%. The overall precision for all GS searches was 0.13% under the assumption of mutual disjointness of the result sets. The results are displayed in detail in Table 1.

**Table 1:** Precision and recall of the Google Scholar searches for studies included in fourteen Cochrane

**Review on**	References included^*^	**References found**	**Hits**	**Precision [%]**	**Recall [%]**
Antidepressants versus placebo for depression in primary care [21]	14	11	17,400	0.06	78.6
Artemisinin-based combination therapy for treating uncomplicated malaria [22]	49	49	5,320	0.92	100
Brief interventions for heavy alcohol users admitted to general hospital wards [23]	14	14	18,000	0.08	100
Combined DTP-HBV-HIB vaccine versus separately administered DTP-HBV and HIB vaccines … [24]	20	20	4,320	0.46	100
Erythropoietin or Darbepoetin for patients with cancer - meta-analysis based on individual patient data [25]	48	41	36,500	0.11	85.4
Green tea (Camellia sinensis) for the prevention of cancer [26]	51	48	17,900	0.29	94.1
Incentive spirometry for prevention of postoperative pulmonary complications in upper abdominal surgery [27]	11	11	13,400	0.08	100
Interventions to prevent occupational noise-induced hearing loss [28]	24	24	19,500	0.12	100
Non-pharmacological interventions for assisting the induction of anaesthesia in children [29]	17	16	29,300	0.05	94.1
Oral iron supplements for children in malaria-endemic areas [30]	70	62	67,800	0.09	88.6
Pharmacotherapy for anxiety disorders in children and adolescents [31]	22	21	17,700	0.12	95.5
Single dose oral flurbiprofen for acute postoperative pain in adults [32]	11	11	7,950	0.14	100
The effects of antimicrobial therapy on bacterial vaginosis in non-pregnant women [33]	24	24	18,700	0.13	100
Therapeutic interventions for symptomatic treatment in Huntington’s disease [34]	21	16	17,400	0.10	76.2
Total	396	368	291,190	0.13	92.9

We used the Cochrane Database of Systematic Reviews issue 3/ 2013 (Gehanno et al. used Issue 3/2009 [9]).

^*^: “References included“ is the number of single references of included studies with one major reference per study as retrieved from the Cochrane reviews.

The precisions of the original searches from the Cochrane reviews (CR searches) are shown in Table 2. Exact information on the origin and number of references from the Cochrane reviews is given in Additional file 1. Each CR reference found in the GS search is marked with an (X) in Additional file 1.

**Table 2:** Combined precision of the Cochrane review searches as retrieved from the Cochrane reviews

**Review**	References found^*^	References included^**^	**Precision [%]**
Arrol [21]	--	14	--
Sinclair [22]	517	49	9.4
McQueen [23]	636	14	2.2
Bar-On [24]	246	20	8.1
Bohlius [25]	5546 (+ 575)	48	0.9
Boehm [26]	675	51	7.6
Guimarães [27]	775	11	1.4
Verbeek [28]	2491	24	1.0
Yip [29]	--	17	--
Okebe [30]	“large number“	70	--
Ipser [31]	1395 (+ 655 + 193)	22	1.6
Sultan [32]	--	11	--
Oduyebo [33]	701	24	3.4
Mestre [34]	102	21	20.6

For more details esp. on the number of searched databases see Additional file 2.

^*^: Usually not specified if doublets are excluded. In brackets “ongoing“ studies which were not further processed.

^**^: “citations included“ is the number of single references of included studies with one major reference per study.

The lengths of the search expressions for the MEDLINE CR searches, the length of the derived GS search expression and their ratio is displayed in Table 3. The length of the MEDLINE CR search expressions ranges between 126 and 1779 characters with a median of 777.5 characters. The length of the GS search expressions lies between 93 and 230 characters with a median of 187.5 characters. The ratio of the lengths of the GS search expressions and the MEDLINE CR search expression ranges between 0.092 and 1.349 with a median of 0.219 and a mean of 0.438.

**Table 3:** Length of search expressions for Cochrane review and Google Scholar searches: number of characters including whitespace

**Review**	Cochrane SR^*^	**Google Scholar**	**Ratio GS/C-SR**
Arrol [21]	1779^**^	230	0.129
Sinclair [22]	126	170	1.349
McQueen [23]	693	144	0.208
Bar-On [24]	387	140	0.362
Bohlius [25]	1209	219	0.181
Boehm [26]	605	218	0.36
Guimarães [27]	862	93	0.108
Verbeek [28]	997	219	0.22
Yip [29]	357	194	0.534
Okebe [30]	135	181	1.341
Ipser [31]	1031	225	0.218
Sultan [32]	1053	97	0.092
Oduyebo [33]	957	150	0.157
Mestre [34]	267	229	0.858

^*^: Number of characters of the documented MEDLINE searches in the Cochrane reviews.

^**^: Number of characters of the search in the Cochrane Collaboration Depression, Anxiety and Neurosis Controlled Trials Registers (CCDANCTR) prior to the search proper.

In Additional file 2 detailed information is provided on the number and type of searched databases and the calculation of the precisions as collected from the Cochrane reviews.

## Discussion

In this study we reinvestigated a recent study of Gehanno et al. [9] with an approach that takes real world search strategies into account. We used the limited search interface of Google Scholar to retrieve result sets derived from the original MEDLINE searches of the Cochrane reviews. Although the relative recall of our search results is very high when compared with professional search results from other databases, the precision of these searches is low. Moreover, due to limitations of the Google Scholar search interface, it is currently not professionally useable in *structured scientific literature retrieval*.

At a first glance, the overall relative recall of Google Scholar of about 93% seems convincing to promote it as a search tool for systematic reviews. Given the high demand in the scientific community, for an easy and consistent search interface for literature retrieval, a unified search interface in the form of Google Scholar would be useful for many researchers. From its launch in 2004, studies compared Google Scholar with other databases for very different purposes and partly reported promising results [45]. Especially the application of Google Scholar with a high recall in clinical contexts was appealing to authors [7,9,46,47]. However, other authors already warned not to make assumptions on the search engine performance based only on retrieval quantities [48].

93% relative recall is a high value for a single database or search interface. However, it may be questioned why we could not observe an even higher relative recall based on the coverage of 100%. The lower recall is clearly attributable to the limited capabilities of Google Scholar’s search interface, as outlined in the introduction. On the one hand, it lacks the possibility to search for arbitrary long disjunctive expressions (terms and phrases connected with OR). On the other hand, it is not possible to freely combine logical subexpression which is a feature often needed when search expressions have to be optimized for both recall and precision.

A good example for the former cause of a lower relative recall is the search for the references of [21]. In the original search for the Cochrane review a large collection of drug names was used in a disjunction of 1,391 characters. In our translated Google Scholar search we could only use the general terms for the disjunction of specific drug names (Antidepressant OR “Monoamine Oxidase Inhibitors” OR “Selective Serotonin Reuptake Inhibitors” OR “Tricyclic Drugs”) due to the limited space of 256 characters. We could not use truncation because Google Scholar lacks this function. With our search we found 11 of 14 included references (relative recall 78.6%). If the specific drug names ‘Mianserin’, ‘Sertralin*’ and ‘Amitriptyline’, which were also used in the original search, were included in the search the recall would be increased to 100%.

The search for [25] is an example illustrating both causes for suboptimal recall in Google Scholar searches. The complex search of this example includes nested expressions for the pathology, the treatment and an elaborated filter for the study design. From the 48 references included in the Cochrane review 41 were found with Google Scholar (relative recall 85.4%). Most of the seven missed references would have been found if the methods filter of the original search could have been elaborated in Google Scholar. This was not possible, again due to the length restriction, but also due to the limitations of Google Scholar to interpret nested logical expressions (e.g., the expression ((singl* or doubl* or trebl* or tripl) adj25 (blind* or mask*)) could not be translated into a nested conjunction as part of a larger disjunction of method related expressions).

Some of the results on Google Scholar are very promising given that Google enhances Google Scholar taking critique into account. If Google professionalizes Google Scholar so that it supports structured search strategies and invests into these not consumer-oriented features, chances are high that Google Scholar could advance to a top position in scientific literature retrieval. It is obvious what can be achieved when state of the art natural language processing technology is paired with superior technological resources. Thus, our work does not intend to derogate the *possibilities* with a Google Scholar approach to literature retrieval but it tries to prevent a much too early “Googlisation” of the domain. Researchers should be aware of what has been achieved in literature retrieval and reporting by constant improvements in information science [14-18].

### Google Scholar is not ready for searches on systematic reviews

We find McGowans et al. statement “Systematic reviews need systematic researchers” [49] transferable from systematic reviews to any type of scientific work. Hence, the inappropriateness of Google Scholar to support *any* systematic and structured literature retrieval process is in the core of our critique. At its current developmental state, Google Scholar does not provide basic mechanism to support scientists in a systematic approach to literature retrieval resulting in consequences - e.g. low precision of search results - which make Google Scholar inappropriate for most structured tasks in literature retrieval.

#### ***Precision matters***

Our results indicate a low precision of the Google Scholar searches. These results are not a surprise given the restrictions of the search interface discussed in the section below. Several authors reported results consistent with ours [50].

Sampson et al. investigated the precision of typical Cochrane systematic reviews. She calculated a mean precision of about 3% for systematic reviews with a large range [51]. Some of the higher precisions we calculated for the original Cochrane reviews investigated in this work may be due to documentation issues (Additional file 2). However, they demonstrate how difficult it is to estimate measures for the quality of retrieval results under real life conditions. Obviously, precision matters as one of the determining factors for the success of systematic review projects with limited resources [52].

Our investigation suggests that due to the low precision of Google Scholar searches a user has to check about 20 times more references on relevance compared to the standard approach using multiple searches in traditional literature databases. In the majority of cases this implies for checking 10,000 or more references. Assuming fast reference checking for exclusion of irrelevant references as a first step of manual study selection [41], an experienced information specialist can check up to 1,000 references a day [53]. If we pragmatically estimate 15–20 working days to perform relevance checking for 10,000 references, the following considerations have to be made prior to a righteous comparison of conventional scientific literature searches with Google Scholar:

• At the current developmental state of Google Scholar, the reference checking of more than 1,000 references is *completely hypothetical* due to Google Scholar’s limitation to display only the first 1000 references! See also the next section.

• If we assume the *counterfactual* retrieval of more than 1000 references from Google Scholar, the following estimates have to be taken into account for a comparison:

– How long does it take to ‘translate’ search expressions between up to 10 different databases for the conventional search? Syntax and semantics of different databases and search interfaces differ largely and an easy translation is rare.

– How long is the duration of conventional retrieval processes (searching, transforming between formats and storing results)?

– How long does it take to check for doublets from the different databases?

– What are the competencies an information specialist needs, to access the different databases and interpret their results?

– What are the economical costs of using certain databases?

Only when taking these factors into account, a realistic comparison is possible. In our view, the low precision of (not optimized) Google Scholar searches is not a *main* argument for the inferiority of Google Scholar. It might even be, that it would be more effective to retrieve large results sets with Google Scholar than to query a number of different databases and merge their results. However, due to Google Scholar’s display and download restrictions this is a scenario which can not be investigated today.

Our results on precision provide only weak evidence that Google Scholar is *limited* as a general search tool for systematic reviews or scientific reviews. We explicitly warn readers to draw premature conclusions only from the high recall of the searches conducted for this work. Although our results on relative recall or the “raw numbers” on coverage reported elsewhere seem impressive, the usability of Google Scholar in structured and systematic literature retrieval might be impaired by the low precision reported. Only with an enhanced search interface the Google Scholar retrieval results can be better optimized for higher precision.

#### ***Limitations of Google Scholar revised***

The three most prominent restrictions of the Google Search interface will be reviewed here in the scope of this work.

• The **limitations of the Google Scholar search syntax** restrain the expressivity of search expressions below what is necessary for most structured retrieval tasks. To give the researcher the means to control what he really wants to find, features like an unlimited (large) size of search expressions, deep nesting of search subexpressions and truncation operators are necessary. In many cases, a high recall can be reached with short and uncomplicated expressions, however, a high precision together with a high recall requires complex expressions in most instances.

• The severe **limitations of the Google Scholar results retrieval** render even the best result sets useless for most projects and subsequent analysis steps. A structured approach requires the possibility to export larger result sets for import in reference management software for doublet checking and scanning by domain experts. Currently it is only possible to manually retrieve the 1000 first results which will be displayed by Google Scholar in 50 steps of 20 references each. This behavior of Google Scholar is outdated in a world where the National Library of Medicine allows the complete download of MEDLINE for scientific purposes.

• The **limitation of the graphical user interface** without a history function and without a convenient search expression builder might be tolerable for ‘power users’ who are accustomed to working with a text editor in combination with a single search expression entry field for dispatching. For the most information specialists and scientists such a work is not acceptable and obstructs their creativity on ‘subject matter’. For the high stakes documentation tasks of current systematic reviews, at least a history function has to be available.

Solutions to most of the restricting features mentioned above are already implemented in Google Scholar and other Google products, but may be artificially restricted or not fully functional in the Google Scholar search interface. Hence, it might be a question of policy and legal issues which hamper Google to provide a full-fledged professional scientific search tool. It was always a main goal of Google to provide the simplest search interface for the average internet user. A high end scientific literature retrieval search interface is a specialized tool for researchers that might not fit in this portfolio. On the other hand, some content providers and publishers might restrict the use of their reference information for Google Scholar users to a kind of “crippled access” to protect their own databases, science portals and search interfaces. Without a clear and trustworthy commitment of Google, Google Scholar will evolve only as a tool with a very limited scope of use. In its current state Google Scholar should not be employed alone in *structured* scientific work which always relies on reliable data.

Although Google Scholar was quickly adopted by the scientific community for its obvious merits and its easy user interface [54], it should be propagated with care for its limited application scope. The scientific community, especially in the biomedical domain, has a high demand for easy to use and reliable search interfaces. If this well justified requirement is met by premature and overly optimistic expectations in new technology, users may tend to leave well approved methodology. Hence, as educators of scientific methodology, we must be careful on how to communicate new technology to users. A goal of our research should be to indicate weaknesses of new technology to their providers. Providers can enhance their tools based on this information so that their product consequently meets *more* criteria for professional scholarly work.

### Limitations of the study

This study is limited due to the small number of included Cochrane reviews from few medical domains and the overall limited size of the alternative gold standard result set of about 400 included references. The results of this study were not intended to be generalizable to all types of literature search or all types of contents. We understand this study as a case series which provides limited evidence for the types of studies investigated here.

As stated before, another important limitation of our study is that we did not fully optimize the applied Google Scholar searches. We developed the searches based on the original MEDLINE searches from the Cochrane reviews with the limitation of the Google Scholar search interface. We only optimized the search expressions for obvious errors in a short iteration cycle. *Optimization of Google Scholar search expression lay outside the scope of this research*. Therefore the reported results, especially on precision, must be read with care in light of potential optimization. It is well possible, to enhance precision conserving high recall further.

### Further research

In the past, studies from a broad field of medical and general scientific subjects were chosen to investigate the performance of Google Scholar. However, even a larger number of studies and reviews from a variety of domains should be retrospectively investigated for the performance of Google Scholar under “real world search conditions”.

Google Scholar search expression optimization is another area for future research. Given the limited capabilities and the nontransparent ranking algorithms, it should be empirically investigated how search expressions can be optimized for precision and recall. It might be possible, that conventional search expression generation established for other scientific databases could be modified for Google Scholar.

To compare the effectiveness of Google Scholar and other retrieval tools, comparative prospective studies are necessary. Only with this design, it becomes possible to compare the systematic search approach of traditional literature retrieval with new methods, especially Google Scholar, with minimized biases. However, depending on information specialists in its implementation, this type of research is methodologically difficult: independent teams of information specialists search for the same research questions with different tools and are eventually compared on their retrieval performance. With this approach, confounding parameters due to unbalanced competencies in the teams can only hardly be controlled. However, with complex (cross-over) designs, the effects of such inter group unbalances could be considered in the estimation of true performance difference due to the search method.

## Conclusion

In this work, we investigated the performance of Google Scholar retrieving studies for systematic reviews. We searched with Google Scholar for references on studies of Cochrane reviews based on their original MEDLINE search expression. Considering the studies included in the Cochrane reviews as a gold standard, we calculated precision and relative recall of the Google Scholar searches.

We measured a 92.9% relative recall for the total of about 400 relative gold standard references. The overall precision was only 0.13%. However, even this low precision might be weighed against the benefit of searching only one resource. In addition, it should be considered that it was not the objective of this study to optimize the queries for precision.

Although the reported relative recall might look impressive, it must be interpreted with care. It is only a quality indicator of Google Scholar in an experimental setting which is, however, not available for systematic retrieval due to the severe limitations of the Google Scholar search interface. Currently, Google Scholar does not provide the necessary elements for systematic scientific literature retrieval like a history function as a tool for incremental query optimization, an export of a large number of references, or a visual search builder. In our view, Google Scholar is not yet ready as a professional searching tool for tasks where structured retrieval methodology is necessary.

## Competing interests

The authors declare that they have no competing interests.

## Authors’ contributions

MB and EM designed the study. MB developed the Google Search expressions and carried out the retrieval experiments. MB and EM analyzed the retrieval results. EM analyzed the Cochrane reviews for precision. MB drafted the manuscript. WV and EM helped to draft the manuscript. All authors read and approved the final manuscript.

## Authors’ information

MB: MD, MME, Computer Scientist. Head of Medical Informatics, Department of Medical Biometry and Medical Informatics at the University Medical Center Freiburg, Germany.

WV: PhD, Professor. Head of the Department of Clinical Epidemiology, Department of Medical Biometry and Medical Informatics at the University Medical Center Freiburg, Germany.

EM: Information Specialist, Scientific Librarian. Department of Medical Biometry and Medical Informatics at the University Medical Center Freiburg, Germany.

## Pre-publication history

The pre-publication history for this paper can be accessed here:

http://www.biomedcentral.com/1471-2288/13/131/prepub

## Supplementary Material

Additional file 1 **List of included studies, original MEDLINE search strategy and derived Google Scholar search strategy for each Cochrane review.** For each Cochrane review the included studies and the original search strategy were extracted to this file. The Google Scholar search expression was used with Google Scholar as provided. In the list of included studies the references which were found with the Google Scholar search expression were marked with an “(X)”. References which were used in more than one study included were marked only once. Results were counted according to this data. Length of search expressions were measured from the given search expressions. Click here for file

Additional file 2 **Detailed information on the original precision of the searches for the Cochrane reviews.** Data retrieved from the Cochrane reviews. Presented are: Number of databases searched and type of additional resources, number of references found with or without doublets where available, number of references included in the Cochrane review, and the calculated precision from the former. If a reference was cited for more than one study it was counted only once in the column “references included”. Click here for file
