# Supplementary material for: Google Scholar as replacement for systematic literature searches: good relative recall and precision are not enough
Source: BMC Med Res Methodol. 2013 Oct 26;13:131. doi: 10.1186/1471-2288-13-131 (PMC3840556; doi:10.1186/1471-2288-13-131)
Supplement: Additional file 1 — List of included studies, original MEDLINE search strategy and derived Google Scholar search strategy for each Cochrane review. For each Cochrane review the included studies and the original search strategy were extracted to this file. The Google Scholar search expression was used with Google Scholar as provided. In the list of included studies the references which were found with the Google Scholar search expression were marked with an “(X)”. References which were used in more than one study included were marked only once. Results were counted according to this data. Length of search expressions were measured from the given search expressions. [file 1471-2288-13-131-S1.pdf]

1_arrol_antidepressants

Reference: Arrol et. al. [@the*cochrane*collaboration*antidepressants*2009]

The Cochrane Depression, Anxiety and Neurosis Group (CCDAN) Controlled Trials Register was searched, together with a supplementary search of MEDLINE, PsycINFO, EMBASE, LILACS, CINAHL and PSYNDEX.

Included Studies

1. **(X) Barge-Schaapveld 2002**
   * Barge-Schaapveld DQ, Nicholson NA. Effects of antidepressant treatment on the quality of daily life: an experience sampling study. *Journal of Clinical Psychiatry* 2002;**63**(6):477–85.

2. **(X) Blashki (150 mg) 1971**
   Blashki TG, Mowbray R, Davies B. Controlled trials (spelling error: it is "trial") of amitriptyline in general practice. *BMJ* 1971;**1**(5741): 133–8.

3. **[same reference as above, excluded from count] Blashki (75mg) 1971**
   Blashki TG, Mowbray R, Davies B. Controlled trials of amitriptyline in general practice. *BMJ* 1971;**1**(5741): 133–8.

4. **Brink 1984**
   Brink CW, van der Krogt JP, Dunbar GC, Behagel LH. A controlled clinical trial of mianserin and placebo in the treatment of depression in general practice. *Tijdschrift voor therapie, geneesmiddel, en onderzoek* 1984;**9**(10):513–7.

5. **Doogan 1994**
   Doogan DP, Langdon CJ. A double-blind, placebo controlled comparison of sertraline and dothiepin in the treatment of major depression in general practice. *International Clinical Psychopharmacology* 1994;**9**(2): 95–100.

6. **Feighner 1979**
   Feighner JP, Brauzer B, Gelenberg AJ, Gomez E, Kiev A, Kurland ML, et al.A placebo-controlled multicenter trial of limbitrol versus its components amitriptyline and chlordiazepoxide. *Psychopharmacology* 1979;**61**(2):217–25.

7. **(X) Hollyman 1988**
   Hollyman JA, Freeling P, Paykel ES, Bhat A, Sedgwick P. Double-blind placebo-controlled trial of amitriptyline among depressed patients in general practice. *Journal of the Royal College of General Practitioners* 1988;**38**(314):393–7.

8. **(X) Lecrubier 1997**
   Lecrubier Y, Bourin M, Moon CA, Schifano F, Blanchard C, Danjou P, et al.Efficacy of venlafaxine in depressive illness. *Acta psychiatrica Scandinavica* 1997;**95**(6):485–93.

9. **(X) Lepola 2001 Citalopram**
   (X) Lepola U, Loft H, Reines EH. Escitalopram is efficacious and well tolerated for the treatment of depression in primary care. Abstract: Annual meeting the American Medical Association. New Orleans. 2001.
   (X) Lepola UM, Loft H, Reines EH. Escitalopram (10-20mg/ day) is effective and well

tolerated in a placebo controlled study in depression in primary care. *International Journal of Clinical Psychopharmacology* 2003;**18**(4):211–7.

10. [same reference as above, excluded from count] Lepola 2001 Escitalopram
(X) Lepola U, Loft H, Reines EH. Escitalopram is efficacious and well tolerated for the treatment of depression in primary care. Abstract: Annual meeting the American Medical Association. New Orleans. 2001.
(X) Lepola UM, Loft H, Reines EH. Escitalopram (10-20mg/ day) is effective and well tolerated in a placebo controlled study in depression in primary care. *International Journal of Clinical Psychopharmacology* 2003;**18**(4):211–7.

11. (X) Malt 1999
Malt UF, Robak OH, Madsbu H-P, Bakke O, Loeb M. The Norwegian naturalistic treatment study of depression in general practice (NORDEP)- randomised double blind study. *BMJ* 1999;**318**(7192):1180–4.

12. (X) Mynors-Wallis 1995
Mynors-Wallis LM, Gath DH, Lloyd-Thomas AR, Tomlinson D. Randomised controlled trial comparing problem solving treatment with amitriptyline and placebo for major depression in primary care. *BMJ* 1995;**310** (6877):441–5.

13. (X) Philipp [commentary on ...] 1999
Philipp M, Kohnen R, Hiller KO. Hypercum extract versus imipramine or placebo in patients with moderate depression: randomised multicentre study of treatment for eight weeks. *BMJ* 1999;**319**(7224):1534–8.

14. (X) Thompson 1989
Thompson C, Thompson CM. The prescribing of antidepressants in General Practice. II: A placebo-controlled trial of low-dose dothiepin. *Human Psychopharmacology* 1989;**4**(3):191–204.

15. (X) Thomson 1982
Thomson J, Rankin H, Ashcroft GW, Yates CM, McQueen JK, Cummings SW. The prescribing of antidepressants in General Practice. II: A placebo-controlled trial of low-dose dothiepin: a comparison of L-tryptophan, amitriptyline, and a combination of L-tryptophan and amitriptyline with placebo. *Pscychological Medicine* 1982;**12**:741–51.

16. [X] Wade 2002
Wade A, Lemming OM, Hedegaard KB. Escitalopram 10mg/day is effective and well tolerated in a placebo-controlled study in depression in primary care. *International Clinical Psychopharmacology* 2002;**17**(3):95–102.

Search strategy

Appendix 1. Search of the CCDANCTR-Studies Register

CCDANCTR-Studies - searched on 24 September 2007
Intervention = (Antidepress* or “Monoamine Oxidase Inhibitors” or “Selective Serotonin Reuptake Inhibitors” or “Tricyclic Drugs” or Acetylcarnitine or Alaproclate or Amersergide or Amiflamine or Amineptine or Amitriptyline or Amoxapine or Befloxatone or Benactyzine or Brofaromine or Bupropion or Butriptyline or Caroxazone or Chlorpoxiten or Cilosamine or Cimoxatone or Citalo- pram or Clomipramine or Clorgyline or Clorimipramine or

Clovoxamine or Deanol or Demexiptiline or Deprenyl or Desipramine or Dibenzipin or Diclofensine or Dothiepin or Doxepin or Duloxetine or Escitalopram or Etoperidone or Femoxetine or Fluotracen or Fluoxetine or Fluparoxan or Fluvoxamine or Idazoxan or Imipramine or Iprindol* or Iproniazid or isocarboxazid or Litoxetin* or Lofepramin* or Maprotilin* or Medifoxamin* or Melitracen or Metapramin* or Mianserin or Milnacipran or Minaprin* or Mir- tazapin* or Moclobemid* or Nefazodon* or Nialamid* or Nomifensin* or Nortriptylin* or Noxiptilin* or Opipramol or Oxaflozan* or Oxaprotilin* or Pargylin* or Paroxetin* or Phenelzin* or Piribedil or Pirlindol* or Pivagabin* or Prosulprid* or Protriptylin* or Quinupramin* or Reboxetin* or Rolipram or Sertralin* or Setiptilin* or Teniloxin* or Tetrindol* or Thiazesim or Thozalinon* or Tianeptin* or Toloxaton* or Tomoxetin* or Tranylcypromin* or Trazodon* or Trimipramin* or Venlafaxin* or Viloxazin* or Viqualin* or Zimeldin*)*
And Intervention = Placebo
And Diagnosis = (Depress* or Dysthymi* or "Adjustment Disorder*" or "Mood Disorder*" or "Affective Disorder" or "Affective Symptoms")
And Setting = ("General Practice" or "Primary Care" or "Community Mental Health" or "Family Practice" or "Health Maintenance Organization" or HMO or Home or "University Clinic" or Private or Ambulatory) And Age Group = Adult

Length: 1793 Characters

Appendix 2. Search of the CCDANCTR-References Register

CCDANCTR-References - searched on 24 September 2007
Free-text = (Antidepress* or "Monoamine Oxidase Inhibitors" or "Selective Serotonin Reuptake Inhibitors" or "Tricyclic Drugs" or Acetylcarnitine or Alaproclate or Amersergide or Amiflamine or Amineptine or Amitriptyline or Amoxapine or Befloxatone or Ben- actyzine or Brofaromine or Bupropion or Butriptyline or Caroxazone or Chlorpoxiten or Cilosamine or Cimoxatone or Citalopram or Clomipramine or Clorgyline or Clorimipramine or Clovoxamine or Deanol or Demexiptiline or Deprenyl or Desipramine or Dibenzipin or Diclofensine or Dothiepin or Doxepin or Duloxetine or Escitalopram or Etoperidone or Femoxetine or Fluotracen or Fluoxetine or Fluparoxan or Fluvoxamine or Idazoxan or Imipramine or Iprindol* or Iproniazid or isocarboxazid or Litoxetin* or Lofepramin* or Maprotilin* or Medifoxamin* or Melitracen or Metapramin* or Mianserin or Milnacipran or Minaprin* or Mir- tazapin* or Moclobemid* or Nefazodon* or Nialamid* or Nomifensin* or Nortriptylin* or Noxiptilin* or Opipramol or Oxaflozan* or Oxaprotilin* or Pargylin* or Paroxetin* or Phenelzin* or Piribedil or Pirlindol* or Pivagabin* or Prosulprid* or Protriptylin* or Quinupramin* or Reboxetin* or Rolipram or Sertralin* or Setiptilin* or Teniloxin* or Tetrindol* or Thiazesim or Thozalinon* or Tianeptin* or Toloxaton* or Tomoxetin* or Tranylcypromin* or Trazodon* or Trimipramin* or Venlafaxin* or Viloxazin* or Viqualin* or Zimeldin*) And Free-text= Placebo* And Keyword = (Depress* or Dysthymi* or "Adjustment Disorder*" or "Mood Disorder*" or "Affective Disorder" or "Affective Symptoms") And Free-text = ("General Practice" or "Primary Care" or "Community Mental Health" or "Family Practice" or "Health Maintenance Organization" or HMO or Home or "University Clinic" or Private or Ambulatory)

Length: 1764 Character

Average Length: 1779 Characters

GOOGLE SCHOLAR SEARCH

depression treatment placebo (Antidepressant OR "Monoamine Oxidase Inhibitors" OR "Selective Serotonin Reuptake Inhibitors" OR "Tricyclic Drugs") ("general practice" OR "primary care") (randomized OR randomised OR random OR trial)

Length: 230

searched on 2013-04-11

Hits: 17,400

16 included studies

14 included references

11 references found

2_sinclair_malaria

`Reference: Sinclair et al. [@the`*`cochrane`*`collaboration`*`artemisinin-based`*`2009]`

Included Studies

1. **(X) Adjei 2006 GHA**
   Adjei GO, Kurtzhals JAL, Rodrigues OP, Alifrangis M, Hoegberg LCG, Kitcher ED, et al.Amodiaquine-artesunate vs artemether-lumefantrine for uncomplicated malaria in Ghanaian children: a randomized efficacy and safety trial with one year follow-up. *Malaria Journal* 2008;**7**(127): DOI: 10.1186/1475-2875-7-127.

2. **(X) Ashley 2003a THA**
   Ashley EA, Krudsood S, Phaiphun L, Srivilairit S, McGready R, Leowattana W, et al.Randomized, controlled dose- optimization studies of dihydroartemisinin-piperaquine for the treatment of uncomplicated multidrug-resistant falciparum malaria in Thailand. Journal of Infectious Diseases 2004; Vol. 190, issue 10:1773–82.

3. **[same reference as above, excluded from count] Ashley 2003b THA**
   Ashley EA, Krudsood S, Phaiphun L, Srivilairit S, McGready R, Leowattana W, et al.Randomized, controlled dose- optimization studies of dihydroartemisinin-piperaquine for the treatment of uncomplicated multidrug-resistant falciparum malaria in Thailand. *Journal of Infectious Diseases* 2004;**190**(10):1773–82.

4. **(X) Ashley 2004 THA**
   Ashley EA, McGready R, Hutagalung R, Phaiphun L, Slight T, Proux S, et al.A randomized, controlled study of a simple, once-daily regimen of dihydroartemisinin-piperaquine for the treatment of uncomplicated, multidrug-resistant falciparum malaria. Clinical Infectious Diseases 2005; Vol. 41, issue 4:425–32.

5. **(X) Ashley 2005 THA**

Ashley EA, Lwin K, McGready R, Simon WH, Phaiphun L, Proux S, et al.An open label randomized comparison
of mefloquine-artesunate as separate tablets vs. a new co- formulatedcombination for the treatment of uncomplicated multidrug-resistant falciparum malaria in Thailand. *Tropical Medicine and International Health* 2006;**11**(11): 1653–60.

6. **(X) Bonnet 2004 GIN** Bonnet M, Roper C, Felix M, Coulibaly L, Kankolongo GM, Guthmann JP. Efficacy of antimalarial treatment in Guinea: in vivo study of two artemisinin combination therapies in Dabola and molecular markers of resistance to sulphadoxine-pyrimethamine in N'Zerekore. Malaria Journal 2007; Vol. 6:54.

7. **(X) Bousema 2004 KEN** Bousema JT, Schneider P, Gouagna LC, Drakeley CJ, Tostmann A, Houben R, et al.Moderate Effect of Artemisinin-Based Combination Therapy on Transmission of Plasmodium falciparum. Journal of Infectious Diseases 2006; Vol. 193, issue 8:1151–9.

8. **(X) Bukirwa 2005 UGA**
Bukirwa H, Yeka A, Kamya MR, Talisuna A, Banek K, Bakyaita N, et al.Artemisinin combination therapies for treatment of uncomplicated malaria in Uganda. PLoS Clinical Trials 2006; Vol. 1, issue 1:e7.

9. **(X) Djimde 2004 MLI** Djimde AA, Fofana B, Sagara I, Sidibe B, Toure S, Dembele D, et al.Efficacy, Safety, and Selection of Molecular Markers of Drug Resistance by Two ACTs in Mali. American Journal of Tropical Medicine and Hygiene 2008; Vol. 78, issue 3: 455–61.

10. **(X) Dorsey 2006 UGA**
Dorsey G, Staedke S, Clark TD, Njama-Meya D, Nzarubara B, Maiteki-Sebuguzi C, et al.Combination therapy for uncomplicated falciparum malaria in Ugandan children: a randomized trial. JAMA 2007; Vol. 297, issue 20:2210–9.

11. **(X) Falade 2005 NGA**
Falade CO, Ogundele AO, Yusuf BO, Ademowo OG, Ladipo SM. High efficacy of two artemisinin-based combinations (artemether-lumefantrine and artesunate plus amodiaquine) for acute uncomplicated malaria in Ibadan, Nigeria. *Tropical Medicine and International Health* 2008; **13**(5):635–643.

12. **(X) Fanello 2004 RWA**
Fanello CI, Karema C, van Doren W, Van Overmeir C, Ngamije D, D'Alessandro U. A randomised trial to assess the safety and efficacy of artemether-lumefantrine (Coartem) for the treatment of uncomplicated Plasmodium falciparum malaria in Rwanda. Transactions of the Royal Society of Tropical Medicine and Hygiene 2007; Vol. 101, issue 4:344–50.

13. **(X) Faye 2003 SEN**
Faye B, Ndiaye JL, Ndiaye D, Dieng Y, Faye O, Gaye O. Efficacy and tolerability of four antimalarial combinations in the treatment of uncomplicated Plasmodium falciparum malaria in Senegal. Malaria Journal 2007; Vol. 6, issue 1: 80.

14. **(X) Grande 2005 PER**
Grande T, Bernasconi A, Erhart A, Gamboa D, Casapia M, Delgado C, et al.A randomised controlled trial to assessthe efficacy of dihydroartemisinin-piperaquine for the treatment of uncomplicated falciparum malaria in Peru. PLoS ONE 2007; Vol. 2, issue 10:e1101.

15. **(X) Guthmann 2003 AGO** Guthmann JP, Ampuero J, Fortes F, van Overmeir C, Gaboulaud

V, Tobback S, et al.Antimalarial efficacy of chloroquine, amodiaquine, sulfadoxine-pyrimethamine, and the combinations of amodiaquine + artesunate and sulfadoxine-pyrimethamine + artesunate in Huambo and Bie provinces, central Angola. Trans R Soc Trop Med Hyg 2005; Vol. 99, issue 7:485–92.

16. **(X) Guthmann 2004 AGO**
Guthmann JP, Cohuet S, Rigutto C, Fortes F, Saraiva N, Kiguli J, et al.High efficacy of two artemisinin-based combinations (artesunate + amodiaquine and artemether + lumefantrine) in Caala, Central Angola. American Journal of Tropical Medicine and Hygiene 2006; Vol. 75, issue 1: 143–5.

17. **(X) Hamour 2003 SDN**
Hamour S, Melaku Y, Keus K, Wambugu J, Atkin S, Montgomery J, et al.Malaria in the Nuba Mountains of Sudan: baseline genotypic resistance and efficacy of the artesunate plus sulfadoxine-pyrimethamine and artesunate plus amodiaquine combinations. Transactions of the Royal Society of Tropical Medicine and Hygiene 2005; Vol. 99, issue 7:548–54.

18. **(X) Hasugian 2005 IDN** Hasugian AR, Purba HL, Kenangalem E, Wuwung RM, Ebsworth EP, Maristela R, et al.Dihydroartemisinin- piperaquine versus artesunate-amodiaquine: superior efficacy and posttreatment prophylaxis against multidrug- resistant Plasmodium falciparum and Plasmodium vivax malaria. Clinical Infectious Diseases 2007; Vol. 44, issue 8: 1067–74.

19. **(X) Hutagalung 2002 THA**
Hutagalung R, Paiphun L, Ashley EA, McGready R, Brockman A, Thwai KL, et al.A randomized trial of artemether-lumefantrine versus mefloquine-artesunate for the treatment of uncomplicated multi-drug resistant Plasmodium falciparum on the western border of Thailand. Malaria Journal 2005; Vol. 4:46.

20. **(X) Janssens 2003 KHM**
Janssens B, van Herp M, Goubert L, Chan S, Uong S, Nong S, et al.A randomized open study to assess the efficacy and tolerability of dihydroartemisinin-piperaquine for the treatment of uncomplicated falciparum malaria in Cambodia. Tropical Medicine and International Health 2007; Vol. 12, issue 2:251–9.

21. **(X) Kamya 2006 UGA**
Kamya MR, Yeka A, Bukirwa H, Lugemwa M, Rwakimari JB, Staedke SG, et al.Artemether-lumefantrine versus dihydroartemisinin-piperaquine for treatment of malaria: a randomized trial. PLoS Clinical Trials 2007; Vol. 2, issue 5: e20.

22. **(X) Karema 2004 RWA** Karema C, Fanello CI, van Overmeir C, van Geertruyden JP, van Doren W, Ngamije D, et al.Safety and efficacy ofdihydroartemisinin/piperaquine (Artekin) for the treatment of uncomplicated Plasmodium falciparum malaria in Rwandan children. Transactions of the Royal Society of Tropical Medicine and Hygiene 2006; Vol. 100, issue 12: 1105–11.

23. **(X) Karunajeewa 2007 PNG** Karunajeewa HA, Mueller I, Senn M, Lin E, Law I, Gomorrai PS, et al.A trial of combination antimalarial therapies in children from Papua New Guinea. *New England Journal of Medicine* 2008;**359**(24):2545–57.

24. **(X) Kayentao 2006 MLI**
Kayentao K, Maiga H, Newman RD, McMorrow ML, Hoppe A, Yattara O, et al.Artemisinin-based combinations versus amodiaquine plus sulphadoxine-pyrimethamine for the treatment

of uncomplicated malaria in Faladje, Mali. *Malaria Journal* 2009;**8**:5.

25. **(X) Kobbe 2007 GHA**
Kobbe R, Klein P, Adjei S, Amemasor S, Thompson WN, Heidemann H, et al.A randomized trial on effectiveness of artemether-lumefantrine versus artesunate plus amodiaquine for unsupervised treatment of uncomplicated Plasmodium falciparum malaria in Ghanaian children. *Malaria Journal* 2008;**7**:261.

26. **(X) Koram 2003 GHA**
Koram KA, Abuaku B, Duah N, Quashie N. Comparative efficacy of antimalarial drugs including ACTs in the treatment of uncomplicated malaria among children under 5 years in Ghana. Acta Tropica 2005; Vol. 95, issue 3: 194–203.

27. **(X) Lefevre 1999 THA**
Lefevre G, Looareesuwan S, Treeprasertsuk S, Krudsood S, Silachamroon U, Gathmann I, et al.A clinical
and pharmacokinetic trial of six doses of artemether- lumefantrine for multidrug-resistant Plasmodium falciparum malaria in Thailand. American Journal of Tropical Medicine and Hygiene 2001; Vol. 64, issue 5–6: 247–56.

28. **(X) Martensson 2003 TZA**
Martensson A, Stromberg J, Sisowath C, Msellem MI, Gil JP, Montgomery SM, et al.Efficacy of artesunate plus amodiaquine versus that of artemether-lumefantrine for the treatment of uncomplicated childhood Plasmodium falciparum malaria in Zanzibar, Tanzania. Clinical Infectious Diseases 2005; Vol. 41, issue 8:1079–86.

29. **(X) Mayxay 2003 LAO** Mayxay M, Khanthavong M, Lindegardh N, Keola S, Barends M, Pongvongsa T, et al.Randomized comparison of chloroquine plus sulfadoxine-pyrimethamine versus artesunate plus mefloquine versus artemether-lumefantrine in the treatment of uncomplicated falciparum malaria in the Lao People's Democratic Republic. Clinical Infectious Diseases 2004; Vol. 39, issue 8:1139–47.

30. **(X) Mayxay 2004 LAO** Mayxay M, Thongpraseuth V, Khanthavong M, Lindegardh N, Barends M, Keola S, et al.An open, randomized comparison of artesunate plus mefloquine vs. dihydroartemisinin-piperaquine for the treatment of uncomplicated Plasmodium falciparum malaria in the Lao People's Democratic Republic (Laos). Tropical Medicine and International Health 2006; Vol. 11, issue 8:1157–65.

31. **(X) Menard 2006 MDG**
Menard D, Andrianina NN, Ramiandrasoa Z, Randriamanantena A, Rasoarilalao N, Jahevitra M, et al.Randomized clinical trial of artemisinin versus non- artemisinin combination therapy for uncomplicated falciparum malaria in Madagascar. Malaria Journal 2007; Vol. 6:65.

32. **(X) Mens 2007 KEN**
Mens PF, Sawa P, van Amsterdam SM, Versteeg I, Omar SA, Schallig HD, et al.A randomized trial to monitor the efficacy and effectiveness by QT-NASBA of artemether-lumefantrine versus dihydroartemisinin-piperaquine for treatment and transmission control of uncomplicated Plasmodium falciparum malaria in western Kenya. *Malaria Journal* 2008;**7**(237):Doi:10.1186/1475–2875-7-237.

33. **(X) Mukhtar 2005 SDN**
Mukhtar EA, Gadalla NB, El-Zaki SE, Mukhtar I, Mansour FA, Babiker A, et al.A comparative study on the efficacy
of artesunate plus sulphadoxine/pyrimethamine versus artemether-lumefantrine in eastern

Sudan. Malaria Journal 2007; Vol. 6:92.

34. **(X) Mutabingwa 2004 TZA**
Mutabingwa TK, Anthony D, Heller A, Hallett R, Ahmed J, Drakeley C, et al.Amodiaquine alone, amodiaquine+sulfadoxine-pyrimethamine, amodiaquine+artesunate, and artemether-lumefantrine for outpatient treatment of malaria in Tanzanian children: a four-arm randomised effectiveness trial. Lancet 2005; Vol. 365, issue 9469:1474–80.

35. **(X) Owusu-Agyei 2006 GHA** Owusu-Agyei S, Asante KP, Owusu R, Adjuik M, Amenga-Etego S, Dosoo DK, et al.An open label, randomised trial of artesunate+amodiaquine, artesunate+chlorproguanil- dapsone and artemether-lumefantrine for the treatment of uncomplicated malaria. *PLoS ONE* 2008;**3**(6):e2530.

36. **(X) Ratcliff 2005 IDN** Ratcliff A, Siswantoro H, Kenangalem E, Maristela R, Wuwung RM, Laihad F, et al.Two fixed-dose artemisinin combinations for drug-resistant falciparum and vivax malaria in Papua, Indonesia: an open-label randomised comparison. Lancet 2007; Vol. 369, issue 9563:757–65.

37. **(X) Sagara 2005b MLI**
Sagara I, Diallo A, Kone M, Coulibaly M, Diawara SI, Guindo O, et al.A randomized trial of artesunate- mefloquine versus artemether-lumefantrine for treatment of uncomplicated Plasmodium falciparum malaria in Mali. *American Journal of Tropical Medicine and Hygiene* 2008;**79** (5):655–61.

38. **(X) Smithuis 2004 MMR**
Smithuis F, Kyaw MK, Phe O, Aye KZ, Htet L, Barends M, et al.Efficacy and effectiveness of dihydroartemisinin- piperaquine versus artesunate-mefloquine in falciparum malaria: an open-label randomised comparison. Lancet 2006; Vol. 367, issue 9528:2075–85.

39. **(X) Staedke 2003 UGA** Staedke SG, Mpimbaza A, Kamya MR, Nzarubara BK, Dorsey G, Rosenthal PJ. Combination treatments for uncomplicated falciparum malaria in Kampala, Uganda: randomised clinical trial. Lancet 2004; Vol. 364, issue 9449:1950–7.

40. **(X) Stohrer 2003 LAO**
Stohrer JM, Dittrich S, Thongpaseuth V, Vanisaveth V, Phetsouvanh R, Phompida S, et al.Therapeutic efficacy of artemether-lumefantrine and artesunate-mefloquine for treatment of uncomplicated Plasmodium falciparum malaria in Luang Namtha Province, Lao People's Democratic Republic. Tropical Medicine and International Health 2004; Vol. 9, issue 11:1175–83.

41. **(X) Swarthout 2004 ZAR**
Swarthout TD, van den Broek IV, Kayembe G, Montgomery J, Pota H, Roper C. Artesunate + amodiaquine and artesunate + sulphadoxine-pyrimethamine for treatment of uncomplicated malaria in Democratic Republic of Congo: a clinical trial with determination of sulphadoxine and pyrimethamine-resistant haplotypes. Tropical Medicine and International Health 2006; Vol. 11, issue 10:1503–11.

42. **(X) Tangpukdee 2005 THA**
Tangpukdee N, Krudsood S, Thanachartwet W, Chalermrut K, Pengruksa C, Srivilairit S, et al.An open randomized clinical trial of Artekin vs artesunate-mefloquine in the treatment of acute uncomplicated falciparum malaria. Southeast Asian Journal of Tropical Medicine and Public Health 2005; Vol. 36, issue 5:1085–91.

43. **(X) Tran 2002 VNM**
Tran TH, Dolecek C, Pham PM, Nguyen TD, Nguyen TT, Le HT, et al.Dihydroartemisinin-piperaquine against multidrug-resistant Plasmodium falciparum malaria in Vietnam: randomised clinical trial. Lancet 2004; Vol. 363, issue 9402:18–22.

44. **(X) Van den Broek 2003a BGD** van den Broek IV, Maung UA, Peters A, Liem L, Kamal M, Rahman M, et al.Efficacy of chloroquine + sulfadoxine-- pyrimethamine, mefloquine + artesunate and artemether + lumefantrine combination therapies to treat Plasmodium falciparum malaria in the Chittagong Hill Tracts, Bangladesh. Transactions of the Royal Society of Tropical Medicine and Hygiene 2005; Vol. 99, issue 10:727–35.

45. **(X) Van den Broek 2004 ZAR** van den Broek I, Kitz C, Al Attas S, Libama F, Balasegaram M, Guthmann JP. Efficacy of three artemisinin combination therapies for the treatment of uncomplicated Plasmodium falciparum malaria in the Republic of Congo. Malaria Journal 2006; Vol. 5:113.

46. **(X) Van Vugt 1998 THA**
van Vugt M, Looareesuwan S, Wilairatana P, McGready R, Villegas L, Gathmann I, et al.Artemether-lumefantrine for the treatment of multidrug-resistant falciparum malaria. Transactions of the Royal Society of Tropical Medicine and Hygiene 2000; Vol. 94, issue 5:545–8.

47. **(X) Yeka 2004 UGA**
Yeka A, Banek K, Bakyaita N, Staedke SG, Kamya MR, Talisuna A, et al.Artemisinin versus nonartemisinin combination therapy for uncomplicated malaria: randomized clinical trials from four sites in Uganda. PLoS Medicine 2005; Vol. 2, issue 7:e190.

48. **(X) Yeka 2007 UGA**
Yeka A, Dorsey G, Kamya MR, Talisuna A, Lugemwa
M, Rwakimari JB, et al.Artemether-lumefantrine versus dihydroartemisinin-piperaquine for treating uncomplicated malaria: a randomized trial to guide policy in Uganda. *PLoS ONE* 2008;**3**(6):e2390.

49. **(X) Zongo 2005 BFA**
Zongo I, Dorsey G, Rouamba N, Tinto H, Dokomajilar C, Guiguemde RT, et al.Artemether-lumefantrine
versus amodiaquine plus sulfadoxine-pyrimethamine for uncomplicated falciparum malaria in Burkina Faso: a randomised non-inferiority trial. Lancet 2007; Vol. 369, issue 9560:491–8.

50. **(X) Zongo 2007 BFA**
Zongo I, Dorsey G, Rouamba N, Dokomajilar C, Sere Y, Rosenthal PJ, et al.Randomized comparison of amodiaquine plus sulfadoxine-pyrimethamine, artemether-lumefantrine, and dihydroartemisinin-piperaquine for the treatment of uncomplicated Plasmodium falciparum malaria in Burkina Faso. Clinical Infectious Diseases 2007; Vol. 45, issue 11: 1453–61.

Detailed search strategy

Length: 115 + 11 = 126

Search set

	CIDG SR[a]	CENTRAL	MEDLINE[b]	EMBASE[b]	LILACS[b]
**1**	malaria	malaria	malaria	malaria	malaria
**2**	arte*	arte*	arte*	arte*	arte*
**3**	dihydroarte*	dihydroarte*	dihydroarte*	dihydroarte*	dihydroarte*
**4**	amodiaq*	amodiaq*	amodiaq*	amodiaq$	amodiaq$
**5**	lumefantrine	lumefantrine	lumefantrine	lumefantrine	lumefantrine
**6**	Coartem*	Coartem*	Coartem*	Coartem$	Coartem$
**7**	mefloquine	mefloquine	mefloquine	mefloquine	mefloquine
**8**	2 or 3	2 or 3	2 or 3	2 or 3	2 or 3
**9**	4 or 5 or 6 or 7	4 or 5 or 6 or 7	4 or 5 or 6 or 7	4 or 5 or 6 or 7	4 or 5 or 6 or 7
**10**	1 and 8 and 9	1 and 8 and 9	1 and 8 and 9	1 and 8 and 9	1 and 8 and 9
**11**	-	-	Limit 10 to humans	Limit 10 to human	-

GOOGLE SCHOLAR SEARCH

(randomized OR randomised OR random) Malaria (artemisinin OR artemether OR hydroartemisinin OR dihydroartemisinin) (amodiaquine OR lumefantrine OR coartem OR mefloquine)

Length: 170

Hits: 5320

50 included studies

49 included references

searched on 2013-04-09

49 references found

3_mcqueen_heavy-alcohol

Reference: `McQueen et al. [@thecochranecollaborationbrief2011]`

```
The study of Gehanno et al. [@gehannois2013] used the version from 2009 with only
11 included studies based on a different search strategy.
```

Included studies

1. **(X) Antti-Poika 1988**
   Antti-Poika I, Karaharju E, Roine R, Salaspuro M. Intervention of heavy drinking - A prospective and controlled study of 438 consecutive injured male patients. *Alcohol and Alcoholism* 1988;**23**(2):115–21.

2. **(X) Chick 1985**
   Chick J, Lloyd G, Crombie E. Counselling problem drinkers in medical wards a controlled study. *British Medical Journal* 1985;**290**:965–7.

3. **(X) Freyer-Adam 2008**
   Freyer-Adam J, Coder B, Baumeister S.E, Bischof G, Riedel J, Paatsch K, et al.Brief alcohol intervention for general hospital inpatients: A randomised controlled trial. *Drug and Alcohol Dependence* 2008;**93**:233–43.

4. **(X) Gentilello 1999**
   Gentilello LM, Rivara FP, Donovan DM, Jurkovich GJ, Daranciag E, et al.Alcohol interventions in a trauma centre as a means of reducing the risk of injury recurrence. *Annals of Surgery* 1999;**230**(4):473–90.

5. **(X) Heather 1996**
   Heather N, Rollnick S, Bell A, Richmond R. Effects of brief counselling among male heavy drinkers identified on general hospital wards. *Drug and alcohol review* 1996;**15**: 29–38.

6. **(X) Holloway 2007**
   Holloway AS, Watson HE, Arthur AJ, Starr G, McFadyn AK, McIntosh J. The effect of brief interventions on alcohol consumption among heavy drinkers in a general hospital setting. *Addiction* 2007;**102**(11):1762–70.

7. **(X) Liu 2011**
   Liu S, Wu S, Chen S, Huang H, Sun F, Fang C, et al.Randomized controlled trial of a brief intervention for unhealthy alcohol use in hospitalised Taiwanese men. *Addiction* 2011;**106**(5): 928–40.

8. **(X) McManus 2003**
   McManus S, Hipkins J, Haddad P, Guthrie E, Creed F. Implementing an effective intervention for problem drinkers on medical wards. *General Hospital Psychiatry* 2003;**25**:332–7.

9. **(X) McQueen 2006**
   McQueen J, Allan L, Mains D. Brief Motivational Counselling for Alcohol Abusers admitted to Medical Wards. *British Journal of Occupational Therapy* 2006;**69**(7): 327–33.

10. **(X) Saitz 2007**
    Saitz R, Palfai TP, Cheng DM, Horton NJ, Freedner N, Dukes K, Kraemer KL, et al.Brief intervention for medical inpatients with unhealthy alcohol use. *Annals of internal medicine* 2007;**146**(3):167–76.

11. **(X) Schermer 2006**
    Schermer CR, Moyers TB, Miller WR, Bloomfield LA. Trauma centre brief interventions for

alcohol disorders decrease subsequent driving under the influence arrests. *The journal of trauma injury infection and critical care* 2006;**60**: 29–34.

12. **(X) Sommers 2006**
Sommers MS, Dyehouse JM, Howe SR, Fleming M, Fargo JD, Schafer JC. Effectiveness of brief interventions after alcohol related vehicular injury: A randomised controlled trial. *The journal of trauma injury infection and critical care* 2006;**61**(3):523–33.

13. **(X) Tsai 2009**
Tsai Y-F, Mei-Chu T, Yea-Pyng L, Ching-Yen C. Brief Intervention for Problem Drinkers in a Chinese Population: A Randomized Controlled Trial in a Hospital Setting. *Alcoholism: Clinical and Experimental Research* 2009;**33**(1): 95–101.

14. **(X) Watson 1999**
Watson HE. A study of minimal interventions for problem drinkers in acute care settings. *International Journal of Nursing Studies* 1999;**36**:425–34.

PubMed search strategy

Subject specific

1. Alcohol-Related Disorders[Mesh]
2. ((alcohol) and (abuse or misuse* or disorder* or drink* or consumption *))*
3. *((hazard* or risk or heav*) AND (drink*))
4. #1or#2or#3
5. "Patient Care"[MeSH]
6. "Patient Admission"[Mesh]
7. Inpatients[MeSH]
8. Hospitals[MeSH]
9. Hospital* or inpatient*

10. #5or#6or#7or#8or#9 11. "brief intervention*"
12. "alcohol reduction"
13. "alcohol intervention"

14. "early intervention"
15. "minimal intervention"
16. counselling or counseling
17. #11or#12or#13or#14or#15or#16
18. randomized controlled trial[pt]
19. controlled clinical trial[pt]
20. Random*[tiab]
21. placebo[tiab]
22. drug therapy [sh]
23. trial [tiab]
24. groups [tiab]
25. #18or#19or#20or#21or#22or#23or#24 26. animals [mh] NOT humans [mh]
27. #25 NOT #26
28. #4 AND #10 AND #17 AND #27

Length: 788 - 9*3* - *17*4 = 693

GOOGLE SCHOLAR SEARCH

("alcohol abuse" OR "alcohol misuse" OR "alcohol disorder" OR "heavy drinking") intervention (controlled OR random OR randomized OR randomised)

Length: 144

Hits: 18,800

14 included studies/references

searched on 2013-04-10

14 references found

4_bar-on_combined-dtp

Reference: Bar-On et. al [@the*cochrane*collaboration*combined*2012]

New version of review has 20 included studies (old version only 18).

Included studies

1. **(X) Aristegui 2003**
   Aristegui J, Dal-Re R, Diez-Delgado J, Mares J, Casanovas JM, Garcia-Corbeira P, et al. Comparison of the reactogenicity and immunogenicity of a combined diphtheria, tetanus, acellular pertussis, hepatitis B, inactivated polio (DTPa-HBV-IPV) vaccine, mixed with the Haemophilus influenzae type b (Hib) conjugate vaccine and administered as a single injection, with the DTPa- IPV/Hib and hepatitis B vaccines administered in two simultaneous injections to infants at 2, 4 and 6 months of age. *Vaccine* 2003;**21**(25-6):3593–600.

2. **(X) Avdicova 2002**
   Avdicova M, Prikazsky V, Hudeckova H, Schuerman L, Willems P. Immunogenicity and reactogenicity of a novel hexavalent DTPa-HBV-IPV/Hib vaccine compared to separate concomitant injections of DTPa-IPV/Hib and HBV vaccines, when administered according to a 3, 5 and 11 month vaccination schedule. *European Journal of Pediatrics* 2002;**161**(11):581–7.

3. **(X) Bravo 1998**
   Bravo L, Carlos J, Gatchalian S, Borja-Tabora C, Bibera G, Willems P, et al.The new DTPw-HBV-Hib combination vaccine can be used at the who schedule with a monovalent dose of

hepatitis B vaccine at birth. *Southeast Asian Journal of Tropical Medicine and Public Health* 1998;**29**(4):772–8.

4. (X) Faingezicht 2002
Faingezicht I, Avila-Aguerro ML, Cervantes Y, Fourneau M, Clemens SA. Primary and booster vaccination with DTPw- HB/Hib pentavalent vaccine in Costa Rican children who had received a birth dose of hepatitis b vaccine. *Pan American Journal of Public Health* 2002;**12**(4):247–57.

5. (X) Gabutti 2004
Gabutti G, Zepp F, Schuerman L, Dentico P, Bamfi F, Soncini R, et al.Evaluation of the immunogenicity and reactogenicity of a DTPa-HBV-IPV. Combination vaccine co-administered with a Hib conjugate vaccine either
as a single injection of a hexavalent combination or as two separate injections at 3, 5 and 11 months of age. *Scandinavian Journal of Infectious Diseases* 2004;**36**(8): 585–92.

6. (X) Gabutti 2005
Gabutti G, Bona G, Dentico P, Bamfi F, Hardt K, Majori S, et al.Immunogenicity and reactogenicity following primary immunisation with a combined DTaP-HBV vaccine and a Haemophilus influenzae type B vaccine administered by separate or mixed injection. *Clinical Drug Investigation* 2005;**25**(5):315–23.

7. (X) Greenberg 2000
Greenberg DP, Wong VK, Partridge S, Chang SJ, Jing
J, Howe BJ, et al.Immunogenicity of a Haemophilus influenzae type b-tetanus toxoid conjugate vaccine when mixed with a diphtheria-tetanus-acellular pertussis-hepatitis B combination vaccine. *Pediatric Infectious Disease Journal* 2000;**19**(12):1135–40.

8. (X) Mallet 2000
Mallet E, Fabre P, Pines E, Salomon H, Staub T, Schodel F, et al.Immunogenicity and safety of a new liquid hexavalent combined vaccine compared with separate administration of reference licensed vaccines in infants. *Pediatric Infectious Disease Journal* 2000;**19**(12): 1119–27.

9. (X) **Marshall 2010**
Marshall H, McIntyre P, Roberton D, Dinan L, Hardt
K. Primary and booster immunization with a diphtheria, tetanus, acellular pertussis, hepatitis B (DTPa-HBV) and Haemophilus influenzae type b (Hib) vaccine administered separately or together is safe and immunogenic. *International Journal of Infectious Diseases* 2010;**14**(1): e41–9. [PUBMED: 19467896]

10. (X) Nolan 2001
Nolan T, Hogg G, Darcy MA, Skeljo M, Carlin J, Boslego J. A combined liquid Hib (PRP-OMPC), hepatitis B, diphtheria, tetanus and whole-cell pertussis vaccine: controlled studies of immunogenicity and reactogenicity. *Vaccine* 2001;**19**(15-16):2127–37.

11. (X) Omenaca 2001
Omenaca F, Dal-Re R, D'Apuzzo V, Kattamis C, Gnehm HP, Garcia-Sicilia J, et al.Reactogenicity of DTPa-HBV/ Hib vaccine administered as a single injection vs DTPa-HBV and Hib vaccines administered simultaneously at separate sites, to infants at 2, 4 and 6 months of age. *Vaccine* 2001;**19**(30):4260–6.

12. (X) Ortega-Barria 2007
Ortega-Barria E, Kanra G, Leroux G, Bravo L, Safary A, Levlevre I. The immunogenicity and

reactogenicity of DTPw-HBV/Hib 2.5 combination vaccine: Results from four phase III multicenter trials across three continents. *Vaccine* 2007;**25**(50):8432–40.

13. (X) Pichichero 1997
Pichichero ME, Passador S. Administration of combined diphtheria and tetanus toxoids and pertussis vaccine, hepatitis B vaccine, and Haemophilus influenzae type b (Hib) vaccine to infants and response to a booster dose of Hib conjugate vaccine. *Clinical Infectious Diseases* 1997;**25** (6):1378–84.

14. (X) Ramkissoon 2001
Ramkissoon A, Coovadia HM, Jugnundan P, Willems P, Clemens BR. A new combined DTP-HBV-Hib vaccine-- strategy for incorporation of Hib vaccination into childhood immunisation programmes. *South African Medical Journal* 2001;**91**(10):864–9.

15. (X) **Rao 2009** ***{published data only}***
Rao R, Dhingra MS, Bavdekar S, Behera N, Daga SR, Dutta AK, et al. A comparison of immunogenicity and safety of indigenously developed liquid (DTwPHB-Hib) pentavalent combination vaccine (Shan 5) with Easyfive (liq) and TritanrixHB + Hiberix (lyo) in Indian infants administered according to the EPI schedule. *Human vaccines* 2009;**5**(6): 425–9. [PUBMED: 19333002]

16. (X) Riedemann 2002
Riedemann S, Reinhardt G, Jara J, Rios R, Wenzel MS, Willems P, et al.Immunogenicity and reactogenicity of combined versus separately administered DTPw-HBV and Hib vaccines given to healthy infants at 2, 4, and 6 months of age, with a booster at 18 months. *International Journal of Infectious Diseases* 2002;**6**(3):215–22.

17. (X) Santos 2002
Santos JI, Martin A, De Leon T, Rivera L, Gaitan ME, Del Rio C, et al.DTPw-HB and Hib primary and booster vaccination: combined versus separate administration to Latin American children. *Vaccine* 2002;**20**(13-4):1887–93.

18. (X) Schmitt 2000
Schmitt HJ, Knuf M, Ortiz E, Sanger R, Uwamwezi MC, Kaufhold A. Primary vaccination of infants with diphtheria- tetanus-acellular pertussis-hepatitis B virus- inactivated polio virus and Haemophilus influenzae type b vaccines given as either separate or mixed injections. *Journal of Pediatrics* 2000;**137**(3):304–12.

19. (X) Tregnaghi 2006
Tregnaghi M, Lopez P, Rocha C, Rivera L, David M, Ruttimann R. A new DTPw-HB/Hib combination vaccine for primary and booster vaccination of infants in Latin America. *Pan American Journal of Public Health* 2006;**19** (3):179–88.

20. (X) Win 1997
Win KM, Aye M, Htay-Htay H, Safary A, et al.Comparison of separate and mixed administration of DTPw-HBV and Hib vaccines: Immunogenicity and reactogenicity profiles. *International Journal of Infectious Diseases* 1997;**2**(2):79–84.

MEDLINE (OVID) SEARCH

1 Diphtheria-Tetanus-Pertussis Vaccine/
2 Diphtheria-Tetanus-acellular Pertussis Vaccines/

3 (diphtheria and tetanus and pertussis).mp.
4 (dtp* or dtap*).tw.
5 1 or 2 or 3 or 4
6 exp Haemophilus Vaccines/
7 exp Haemophilus influenzae type b/
8 exp HAEMOPHILUS/
9 (haemophilus or hemophilus).mp.
10 Hib.mp.
11 or/6-10
12 exp Hepatitis B Vaccines/
13 exp Hepatitis B/
14 (hepatitis b or HBV).mp.
15 or/12-14
16 5 and 11 and 15

Length: 426 - 92 - 73 = 387

GOOGLE SCHOLAR SEARCH

diphtheria tetanus (pertussis OR dtp OR dtap) (haemophilus OR hemophilus OR hib) ("hepatitis b" OR HBV) (random OR randomized OR randomised)

Length: 140

Hits: 4320

20 studies/references included

searched on 2013-04-10

20 references found

5_bohlius_cancer-erythropoietin

Reference: `Bohlius et al. [@thecochranecollaborationerythropoietin2009]`

Studies included

1. **(X) Aapro 2008**
   Aapro M, Barnadas A, Leonard RC, Marangolo M, Untch M. Effects of epoetin beta treatment in patients with metastatic breast cancer receiving chemotherapy. Results of the BRAVE trial. Breast Cancer Research and Treatment 2006; Vol. 100:abstract 6095.
   * Aapro M, Leonard RC, Barnadas A, Marangolo M, Untch M, Malamos N, et al.Effect of once-weekly epoetin beta on survival in patients with metastatic breast cancer receiving

anthracycline- and/or taxane-based chemotherapy: Results of the Breast Cancer-Anemia and the Value of Erythropoietin (BRAVE) Study. *Journal of Clinical Oncology* 2008;**26**(4):592–8.

2. (X) Abels 1993
* Abels R. Erythropoietin for anemia in cancer patients. *European Journal of Cancer* 1993;**29a**(Suppl 2):2–8.
Abels R. Recombinant Human Erythropoietin in the Treatment of the Anaemia of Cancer. *Acta Haematologica* 1992;**87**(Suppl 1):4–11.
Abels RI, Larholt KM, Krantz KD, Bryant EC. Recombinant Human Erythropoietin (rHuEPO) for the Treatment of the Anemia of Cancer. *Oncologist* 1996;**1**(3): 140–50.

3. (X) Boogaerts 2003
* Boogaerts M, Coiffier B, Kainz C, and the Epoetin beta QOL Working Group. Impact of epoetin beta on quality of life in patients with malignant disease. *British Journal of Cancer* 2003;**88**(7):988–95.
Coiffier B. Epoetin beta (Neorecormon©) improves quality of life in cancer-associated anaemia to a similar degree in patients with lymphoid malignancies or solid tumours. Proceedings of the 8th Congress of the European Haematology Association 2003:abstract 0153.
Coiffier B, Boogaerts M, Kainz C. Impact of epoetin beta versus standard care on quality of life in patients with malignant disease. *Proceedings of the 6th Congress of the European Haematology Association* 2001;**2**(Suppl 1):abstract 194.

4. (X) Case 1993
Abels RI, Larholt KM, Krantz KD, Bryant EC. Recombinant Human Erythropoietin (rHuEPO) for the Treatment of the Anemia of Cancer. *Oncologist* 1996;**1**(3): 140–50.
* Case DC, Bukowski RM, Carey RW, Fishkin EH, Henry DH, Jacobson RJ, et al.Recombinant human erythropoietin therapy for anemic cancer patients on combination hemotherapy. *Journal of the National Cancer Institute* 1993; **85**(10):801–6.

5. (X) Cazzola 1995
Cazzola M, Messinger D, Battistel V, Bron D, Cimino R, Enller Ziegler L, et al.Recombinant human erythropoietin in the anemia associated with multiple myeloma or non- Hodgkin's lymphoma: dose finding and identification of predictors of response. *Blood* 1995;**86**(12): 4446–53.

6. (X) CC2574-P-174
* Luksenburg H, Weir A, Wager R. P-174 in: Safety concerns associated with Aranesp (darbepoetin alfa) Amgen, Inc. and Procrit (epoetin alfa) Ortho Biotech, L.P., for the treatment of anemia associated with cancer chemotherapy. Department of Health and Human Services, Food and Drug Administration, Center for Drug Evaluation and Research, Oncologic Drugs Advisory Committee Rockville (MD), USA, 2004; Vol. http://www.fda.gov/ohrms/dockets/ac/ 04/briefing/4037b2˙04.pdf [date of last access March 27, 2009].
Pangalis GA, Poziopoulos C, Angelopoulou MK, Siakantaris MP, Panayiotidis P. Effective treatment of disease-related anaemia in B-chronic lymphocytic leukaemia patients with recombinant human erythropoietin. *British Journal of Haematology* 1995;**89**(3):627–9.

7. (X) Chang 2005
Chang J, Couture F, Young S, McWatters KL, Lau CY. Weekly epoetin alfa maintains hemoglobin, improves quality of life, and reduces transfusion in breast cancer patients receiving chemotherapy. *Journal of Clinical Oncology* 2005;**23**(12):2597–605.

8. (X) Charu 2007
Charu V, Belani CP, Gill AN, Bhatt M, Ben Jacob A, Tomita D, et al.A controlled,

randomized, open-label study to evaluate the effect of every-2-week darbepoetin alfa for anemia of cancer. Annual Meeting Proceedings of the American Society of Clinical Oncology 2004:abstract 8084. * Charu V, Belani CP, Gill AN, Bhatt M, Tomita D, Rossi G, et al.Efficacy and safety of every-2-week darbepoetin alfa in patients with anemia of cancer: a controlled, randomized, open-label phase II trial. *Oncologist* 2007;**12**(6):727–37. Charu V, Saidman B, Ben Jacob A, Justice GR, Maniam AS, Rearden T, et al.Improvements in fatigue are associated with early treatment with darbepoetin alfa every 3 weeks in anemic patients receiving chemotherapy. *The Journal of Supportive Oncology* 2005;**3**(2 Suppl 1):14–5.

9. **(X) Dammacco 2001**
* Dammacco F, Castoldi G, Rodjer S. Efficacy of epoetin alfa in the treatment of anaemia of multiple myeloma. *British Journal of Haematology* 2001;**113**(1):172–9. Dammacco F, Silvestris F, Castoldi GL, Grassi B, Bernasconi C, Nadali G, et al.The effectiveness and tolerability of epoetin alfa in patients with multiple myeloma refractory to chemotherapy. *International Journal of Clinical and Laboratory Research* 1998;**28**:127–34.

10. **Debus 2006**
* Debus J, Hindermann S, Morr H, Mezger J, Sebastian M, Angermund R, et al.Epoetin alfa (EPO) and survival in patients with non-resectable NSCLC - Interim results. 27th Congress of the German Cancer Society Berlin, Germany, 2006. German Medical Science 2006:abstract PO147. Debus J, Hindermann S, Morr H, Mezger J, Sebastian M, Angermund R, et al.Epoetin alfa (EPO) and survival in patients with non-resectable NSCLC - Interim results. Lung Cancer 2005; Vol. 49, issue Suppl 3:S57.

11. **[same reference as above, excluded from count] EPO-GBR-7**
Luksenburg H, Weir A, Wager R. EPO-GBR-7: Safety concerns associated with Aranesp (darbepoetin alfa) Amgen Inc. and Procrit (epoetin alfa) Ortho Biotech L.P., for the treatment of anemia associated with cancer chemotherapy. Department of Health and Human Services, Food and Drug Administration, Center for Drug Evaluation and Research, Oncologic Drugs Advisory Committee, Rockville (MD), USA, 2004; Vol. http://www.fda.gov/ohrms/dockets/ac/ 04/briefing/4037b2˙04.pdf [date of last access March 27, 2009].

12. **[unpublished, personal communication, not retrievable, excluded from count] EPO-GER-20**
EPO-GER-20. Prospective, randomized, controlled, open phase-IV study on the treatment of small cell lung cancer (SCLC) in the extensive disease (ED) stage per VALGB classification with doxorubicin, cyclophosphamide, etoposide (ACE regimen). unpublished: Angermund R, Janssen-Cilag, personal communication.

13. **[same reference as above, excluded from count] EPO-INT-1**
Luksenburg H, Weir A, Wager R. EPO-INT-1: Safety concerns associated with Aranesp (darbepoetin alfa) Amgen, Inc. and Procrit (epoetin alfa) Ortho Biotech, L.P., for the treatment of anemia associated with cancer chemotherapy. Department of Health and Human Services, Food and Drug Administration, Center for Drug Evaluation and Research, Oncologic Drugs Advisory Committee, Rockville (MD), USA, 2004; Vol. http://www.fda.gov/ohrms/dockets/ac/ 04/briefing/4037b2˙04.pdf [date of last access March 27, 2009].

14. **[same reference as above, excluded from count] EPO-INT-3**
Luksenburg H, Weir A, Wager R. EPO-INT-3: Safety concerns associated with Aranesp (darbepoetin alfa) Amgen, Inc. and Procrit (epoetin alfa) Ortho Biotech, L.P., for the treatment of anemia associated with cancer chemotherapy. Department of Health and Human Services, Food and Drug Administration, Center for Drug Evaluation and Research,

Oncologic Drugs Advisory Committee Rockville (MD), USA, 2004; Vol. http://www.fda.gov/ohrms/dockets/ac/ 04/briefing/4037b2˙04.pdf [date of last access March 27, 2009].

15. **(X) Gordon 2006**
Gordon D, Nichols G, Ben Jacob A, Tomita D, Lillie T, Miller C. Treating anemia of cancer with every-4-week darbepoetin alfa: Final efficacy and safety results from a phase II, randomized, double-blind, placebo-controlled study. *The Oncologist* 2008;**13**(6):715–24.
* Gordon DH, Nichols G, Ben Jacob A, Lam H, Lillie T, Miller C. Treating anemia of cancer with darbepoetin alfa administered every 4 weeks: Final results from a phase
2, randomized, double-Blind, placebo-controlled study
in cancer patients not receiving chemotherapy and/or radiotherapy. Blood 2006; Vol. 108, issue 11 Suppl:abstract 1304.

16. **(X) Goss 2005**
* Goss G, Feld R, Bezjak A, Perry G, Melosky B, Smith C, et al.Impact of maintaining Hb with epoetin alfa on time to progression (TTP), overall survival (OS), quality of life (QOL) and transfusion reduction in limited disease SCLC patients. Lung cancer 2005; Vol. 49, issue Suppl 2:S53. Luksenburg H, Weir A, Wager R. EPO-CAN-15: Safety concerns associated with Aranesp (darbepoetin alfa) Amgen Inc and Procrit (epoetin alfa) Ortho Biotech L.P. for the treatment of anemia associated with cancer chemotherapy. Department of Health and Human Services, Food and Drug Administration, Center for Drug Evaluation and Research, Oncologic Drugs Advisory Committee, Rockville (MD), USA, 2004; Vol. http://www.fda.gov/ohrms/dockets/ac/ 04/briefing/4037b2˙04.pdf [date of last access March 27, 2009].

17. **(X) Grote 2005**
* Grote T, Yeilding AL, Castillo R, Butler D, Fishkin E, Henry DH, et al.Efficacy and safety analysis of epoetin
alfa in patients with small-cell lung cancer: a randomized, double-blind, placebo-controlled trial. *Journal of Clinical Oncology* 2005;**23**(36):9377–86.
Luksenburg H, Weir A, Wager R. N93-004: Safety concerns associated with Aranesp (darbepoetin alfa) Amgen Inc and Procrit (epoetin alfa) Ortho Biotech L.P. for the treatment of anemia associated with cancer chemotherapy. Department of Health and Human Services, Food and Drug Administration, Center for Drug Evaluation and Research, Oncologic Drugs Advisory Committee, Rockville (MD), USA, 2004; Vol. http://www.fda.gov/ohrms/dockets/ac/ 04/briefing/4037b2˙04.pdf [date of last access March 27, 2009].

18. **(X) Hedenus 2003**
20000161. Continuing reassessment of the risks of erythropoiesis-stimulating agents (ESAs) administered
for the treatment of anemia associated with cancer chemotherapy. Department of Health and Human Services, Food and Drug Administration, Center for Drug Evaluation and Research, Oncologic Drugs Advisory Committee, Rockville (MD), USA, 2007; Vol. http://www.fda.gov/ohrms/dockets/ac/07/briefing/2007–4301b2–02– FDA.pdf.
* Hedenus M, Adriansson M, San Miguel J, Kramer MH, Schipperus MR, Juvonen E, et al.Efficacy and safety of darbepoetin alfa in anaemic patients with lymphoproliferative malignancies: a randomized, double- blind, placebo-controlled study. *British Journal of Haematology* 2003;**122**(3):394–403.
Hedenus M, Brandberg Y, Molostova V, Iosova G, Abdulkadyrov K, Messinger D, et al.Efficacy of epoetin beta in treating the anaemia of cancer in patients with haematological malignancies. Proceedings of the 6th Congress of the European Haematology Association 2001: abstract 190.

19. **(X) Henke 2003**

* Henke M, Laszig R, Ruebe C, Schaefer U, Haase KD, Schilcher B, et al.Erythropoietin to treat head and neck cancer patients with anaemia undergoing radiotherapy: randomised, double-blind, placebo-controlled trial. *Lancet* 2003;**362**:1255–60.
Schipper J, Henke M. Erythropoietin in patients with head and neck carcinomas? [Erythropoetin bei Karzinomen im Kopf–/Halsbereich?]. *Laryngorhinootologie* 2004;**83**(5): 292–7.

20. (X) Henry 1995
Abels RI, Larholt KM, Krantz KD, Bryant EC. Recombinant Human Erythropoietin (rHuEPO) for the Treatment of the Anemia of Cancer. *Oncologist* 1996;**1**(3): 140–50.
Henry DH, Abels RI. Recombinant human erythropoietin in the treatment of cancer and chemotherapy-induced anemia: Results of double-blind and open-label follow-up studies. *Seminars in Oncology* 1994;**21**(2 (Suppl 3)):21–8.
* Henry DH, Brooks BJ, Case DC, Fishkin E, Jacobson R, Keller AM, et al.Recombinant Human Erythropoietin Therapy for Anemic Cancer Patients Receiving Cisplatin Chemotherapy. *The Cancer Journal from Scientific American* 1995;**1**:252–60.

21. (X) Huddart 2002
Huddart RA, Welch RS, Chan S, Perren T, Atkinson R. A prospective randomised comparative-group evaluation of epoetin alfa for the treatment of anaemia in UK cancer patients receiving platinum-based chemotherapy. Annals of Oncology 2002; Vol. 13 (suppl 5):177.

22. (X) Kotasek 2002
* Kotasek D, Albertsson M, Mackey J, Darbepoetin Alfa 980291 Study Group. Randomized, double-blind, placebo-controlled, dose-finding study of darbepoetin alfa administered once every 3 (Q3W) or 4 (Q4W) weeks in patients with solid tumors. Proceedings of the American Society of Clinical Oncology 2002; Vol. 21:356a.
Kotasek D, Albertsson M, Mackey J, Steger G, Rossi G, O'Byne J, et al.Once per cycle dosing of darbepoetin alfa is feasible in anemic cancer patients receiving chemotherapy. Annals of Oncology 2002; Vol. 13, issue Suppl 5:170.

23. (X) Kotasek 2003
Kotasek D, Berg R, Poulsen E, Colowick A. Randomized, double-blind, placebo controlled, phase I/II dose finding study of ARANESP (TM) administered once every three weeks in solid tumor patients. Blood 2000; Vol. 96, issue 11:abstract 1268.
* Kotasek D, Steger G, Faught W, Underhill C, Poulsen E, Colowick AB, et al.Darbepoetin alfa administered every 3 weeks alleviates anaemia in patients with solid tumours receiving chemotherapy; results of a double-blind, placebo- controlled, randomised study. *European Journal of Cancer* 2003;**39**(14):2026–34.

24. (X) Leyland-Jones 2003
* Leyland-Jones B. Breast cancer trial with erythropoietin terminated unexpectedly. *Lancet Oncology* 2003;**4**:459–60. Leyland-Jones B, Semiglazov V, Pawlicki M, Pienkowski T, Tjulandin S, Manikhas G, et al.Maintaining normal hemoglobin levels with epoetin alfa in mainly nonanemic patients with metastatic breast cancer receiving first-line chemotherapy: A survival study. *Journal of Clinical Oncology* 2005;**23**(25):5865–8.

25. (X) Littlewood 2001
Aapro MS, Cella D, Zagari M. Age, anemia, and fatigue. *Seminars in Oncology* 2002;**29**(3 Suppl 8):55–9.
Fairclough DL, Gagnon DD, Zagari MJ, Marschner N, Dicato M. Evaluation of quality of life

in a clinical trial with nonrandom dropout: the effect of epoetin alfa in anemic cancer patients. *Quality of Life Research* 2003;**12**(8): 1013–27.
Fallowfield L, Gagnon D, Zagari M, Cella D, Bresnahan B, Littlewood TJ, et al.Multivariate regression analyses of data from a randomised, double-blind, placebo-controlled study confirm quality of life benefit of epoetin alfa in patients receiving non-platinum chemotherapy. *British Journal of Cancer* 2002;**87**(12):1341–53.
Fallowfield L, Gagnon D, Zagari M, Cella D, Bresnahan B, Littlewood TJ, et al.Multivariate regression analyses of data from a randomised, double-blind, placebo-controlled study confirm quality of life benefit of epoetin alfa in patients receiving non-platinum chemotherapy. *British Journal of Cancer* 2002;**87**(12):1341–53.
Littlewood TJ. Efficacy and quality of life outcomes of epoetin-alpha in a double-blind, placebo-controlled, multicentre study of cancer patients receiving non- platinum-containing chemotherapy. *Frontiers of radiation therapy and oncology* 2002;**37**:34–7.
* Littlewood TJ, Bajetta E, Nortier JW, Vercammen
E, Rapoport B. Effects of epoetin alfa on hematologic parameters and quality of life in cancer patients receiving non-platinum chemotherapy: Results of a randomized, double-blind, placebo-controlled trial. *Journal of Clinical Oncology* 2001;**19**(11):2865–74.
Littlewood TJ, Cella D, Nortier JW. Erythropoietin improves quality of life. *Lancet Oncology* 2002;**3**(8):459–60. Littlewood TJ, Kallich JD, San Miguel J, Hendricks L, Hedenus M. Efficacy of darbepoetin alfa in alleviating fatigue and the effect of fatigue on quality of life in anemic patients with lymphoproliferative malignancies. *Journal of Pain & Symptom Management* 2006;**31**(4):317–25. Littlewood TJ, Nortier J, Rapoport B, Pawlicki M, de Wasch G, Vercammen E, et al.Epoetin alfa corrects anemia and improves quality of life in patients with hematologic malignancies receiving non-platinum chemotherapy. *Hematological Oncology* 2003;**21**(4):169–80.
Martin SC, Gagnon DD, Zhang L, Bokemeyer C, Van Marwijk KM, van Hout B. Cost-utility analysis of survival with epoetin-alfa versus placebo in stage IV breast cancer. *Pharmacoeconomics* 2003;**21**(16):1153–69.

26. **(X) Machtay 2007**
Machtay M, Pajak T, Suntharalingam M, Hershock D, Stripp DC, Cmelak A, et al.Definitive radiotherapy +/- erythropoietin for squamous cell carcinoma of the head and neck: preliminary report of RTOG 99-03. *International Journal of Radiation Oncology, Biology and Physics* 2004;**60** (Suppl 1):132.
Machtay M, Pajak TF, Suntharalingam M, Shenouda
G, Hershock D, Stripp DC, et al.Radiotherapy with or without erythropoietin for anemic patients with head and neck cancer: a randomized trial of the Radiation Therapy Oncology Group (RTOG 99-03). *International Journal of Radiation Oncology, Biology, Physics* 2007;**69**(4):1008–17.

27. **(X) Milroy 2003**
Milroy R, Scagliotti G, van den Berg PM, Galanis NE, Gómez RG, Greil R, et al.Early intervention with epoetin alfa maintains hemoglobin (HB) in advanced non-small-cell lung cancer (NSCLC) patients. Lung Cancer 2003; Vol. 41, issue Suppl 2:S74.

28. **(X) Moebus 2007**
Moebus V, Bastert G, Kreienberg R, Eidtmann H, Cierna M, Untch M, et al.Epoetin Alpha Prevents Anemia and Transfusions of RBCS in Patients Receiving Dose-Dense Sequential Chemotherapy. Proceedings of the American Society of Clinical Oncology 2001; Vol. 20:abstract 36.
* Moebus V, Lueck H, Thomssen C, Harbeck N, Nitz U, Kreienberg R, et al.The impact of epoetin-alpha on anemia, red blood cell (RBC) transfusions, and survival in breast cancer patients (pts) treated with dose-dense sequential chemotherapy: Mature results of an AGO

phase III study (ETC trial). Journal of Clinical Oncology 2007; Vol. 25, issue 18 Suppl:569.
Moebus V, Untch M, du Bois A, Lueck HJ, Thomssen C, Kuhn W, et al.Dose-dense sequential chemotherapy with epirubicin(E), paclitaxel (T) and cyclophosphamide (C) (ETC) is superior to conventional dosed chemotherapy in high-risk breast cancer patients (>= 4 +LN). First results of an AGO-trial. Journal of Clinical Oncology 2004; Vol. 22, issue 14 Suppl:abstract 513.
Untch M, Jackisch C, Lenhard MS, du Bois A, Lueck HJ, Thomssen C, et al.Epoetin-alpha reduces red blood cell transfusions (RBC) in high-risk breast cancer patients with adjuvant dose-dense, sequential chemotherapy with epirubicin (E), paclitaxel (T) and cyclophosphamide (C) (ETC). Journal of Clinical Oncology 2005; Vol. 23, issue 16 Suppl:613.

29. **(X) O'Shaugnessy 2005**
O'Shaugnessy J, Vukelja S, Savin M, Holmes FA, Jones M, Royall D, et al.Effects of epoetin alfa (Procrit) on cognitive function, mood, asthenia, and quality of life in women with breast cancer undergoing adjuvant or neoadjuvant chemotherapy: a double-blind, randomized, placebo- controlled trial. Proceedings of the American Society of Clinical Oncology 2002:abstract 1449.
O'Shaugnessy J, Vukelja S, Savin M, Holmes FA, Jones M, Royall D, et al.Impact of epoetin alfa on cognitive function, asthenia, and quality of life in women with breast cancer receiving adjuvant or neoadjuvant chemotherapy: analysis of 6-month follow-up data. Proceedings from the 25th Annual Breast Cancer Symposium 2002:abstract: 550.
* O'Shaugnessy J, Vukelja SJ, Holmes FA, Savin M, Jones M, Royall D, et al.Feasibility of quantifying the effects of epoetin alfa therapy on cognitive function in women with breast cancer undergoing adjuvant or neoadjuvant chemotherapy. *Clinical Breast Cancer* 2005;**5**(6):439–46.

30. **[unpublished, personal communication, not retrievable, excluded from count] OBE/EPO-INT-03** OBE/EPO-INT-03. A randomized comparison of the effect of maintaining haemoglobin levels with weekly epoetin alfa or with conventional anaemia management in subjects with Multiple Myeloma undergoing chemotherapy (EMMY). unpublished: Murphy R, Janssen-Cilag, personal communication.

31. **(X) Oberhoff 1998**
Oberhoff C, Krumeich B, Winkler UH, Hoffmann O, Schindler AE. Recombinant human erythropoietin (epoetin beta) in the treatment of chemotherapy - associated anaemia: effects on blood coagulation and fibrinolysis in patients with gynecological malignancies. Annals of Hematology 2000; Vol. 79 Suppl 3:B16.
Oberhoff C, Neri B, Amadori D, Petry KU, Gamucci T, Rebmann U, et al.Recombinant human erythropoietin in the treatment of chemotherapy- induced anemia and prevention of transfusion requirement associated with solid tumors: a randomized, controlled study. *Annals of Oncology* 1998;**9**(3): 255–60.
Oberhoff C, Stauch M, Wilhelm G, Musch E, Heinrich B, Neise M, et al.Prevention and therapy of anemia in tumor patients with Epoetin beta (NeoRecormon). *Tumor Diagnostik und Therapie* 2005;**26**(4):166–71.

32. **(X) Osterborg 1996**
Osterborg A, Boogaerts MA, Cimino R, Essers U, Holowiecki J, Juliusson G, et al.Recombinant human erythropoietin in transfusion-dependent anemic patients with multiple myeloma and non-Hodgkin's lymphoma - a randomized multicenter study. *Blood* 1996;**87**(7): 2675–82.

33. (X) Osterborg 2002
Osterborg A, Brandberg Y, Hedenus M. Impact of epoetin-ß on survival of patients with lymphoproliferative malignancies: long-term follow up of a large randomized study. *British Journal of Haematology* 2005;**129**:206–9.
* Osterborg A, Brandberg Y, Molostova V, Iosava G, Abdulkadyrov K, Hedenus M, et al.Randomized, double- blind, placebo-controlled trial of recombinant human erythropoietin, epoetin beta, in hematologic malignancies. *Journal of Clinical Oncology* 2002;**20**(10):2486–94.

34. (X) Pirker 2008
Amgen. Aranesp(R) “145 Study” Shows No Difference in Survival in Patients with Small-Cell Lung Cancer. Amgen Thousand Oaks, CA, 2007; Vol. http:// wwwext.amgen.com/media/media˙pr˙detail.jsp?year= 2007&releaseID=987476 [date of last access April 28, 2009].
* Pirker R, Ramlau RA, Schuette W, Zatloukal P, Ferreira I, Lillie T, et al.Safety and Efficacy of Darbepoetin Alfa in Previously Untreated Extensive-Stage Small-Cell Lung Cancer Treated With Platinum Plus Etoposide. *Journal of Clinical Oncology* 2008;**26**(14):2342–9.

35. Pronzato 2002
Pronzato P, Cortesi E, van der Rijt C, Moreno-Nogueria A, Raimundo D, Ostler P, et al.Early intervention with epoetin alfa in breast cancer (BC) patients (pts) undergoing chemotherapy (CT): results of a randomized, multicenter, phase IIIb study (EPO-INT-47 Study Group). Annals of Oncology 2002; Vol. 13, issue Suppl 5:168.

36. Quirt 1996
Quirt I, Micucci S, Moran LA, Pater J, Browman G.
The role of recombinant human erythropoietin (EPO) in reducing red blood cell transfusions and maintaining quality of life (QOL) in patients with lymphoma and solid tumors receiving cytotoxic chemotherapy. Results of a randomized, double-blind, placebo-controlled clinical trial. Blood 1996; Vol. 88, issue 10 Suppl 1:347a, abstract 1378.

37. (X) Ray-Coquard 2006
Ray-Coquard I, Dussart S, Goillot C, Mayeur D, Debourdeau P, Ghesquieres H, et al.A risk model for severe anemia to select cancer patients for primary prophylaxis with epoetin {alpha}: a prospective randomized controlled trial of the ELYPSE study group. Annals of Oncology 2009, issue doi:10.1093/annonc/mdn750.
Ray-Coquard I, Perol D, Debourdeau P, Chabaud S, Chelghoum M, Mayeur D, et al.ELYPSE 4: A prospective randomized trial comparing Epo A in primary prophylaxis of severe anemia requiring red cells transfusion in high risk patients. *Annals of Oncology* 2006;**17**(Suppl 9):ix294.

38. (X) Razzouk 2006
Hinds PS, Hockenberry M, Feusner J, Hord JD, Rackoff W, Rozzouk BI. Hemoglobin response and improvements in quality of life in anemic children with cancer receiving myelosuppressive chemotherapy. *Journal of Supportive Oncology* 2005;**3**(6 Suppl 4):10–1.
Razzouk BI, Hockenberry M, Hinds PS, Feusner J, Rackoff W, Hord JD. Influence of hemoglobin response to epoetin alfa on quality-of-life in anemic children with cancer receiving myelosuppressive chemotherapy. Blood 2004; Vol. 104, issue 11:abstract 221.
Razzouk BI, Hockenberry M, Hinds PS, Rackoff W, Hord JD. A double-blind, placebo-controlled study of once- weekly epoetin alfa in children with cancer undergoing myelosuppressive chemotherapy. Journal of Clinical Oncology 2004; Vol. 22, issue 14S:abstract 8527.
* Razzouk BI, Hord JD, Hockenberry M, Hinds PS, Feusner J, Williams D, et al.Double-blind, placebo- controlled study of quality of life, hematologic end points, and safety of weekly epoetin alfa in children with cancer receiving myelosuppressive chemotherapy. *Journal of*

Clinical Oncology 2006;**24**(22):3583–9.

39. (X) Rose 1994
Rose E, Rai K, Revicki D, et al.Clinical and health status assessments in anemic chronic lymphocytic leukemia (CLL) patients treated with epoetin alfa (EPO). Blood 1994; Vol. 84, issue 10 Suppl 1:526a.

40. (X) Savonije 2005
Savonije J, Van Groeningen C, Van Bochove A, Pinedo
H, Giaccone G. Early intervention with epoetin-alfa during platinum-based chemotherapy. Journal of Clinical Oncology 2004; Vol. 22, issue 14S:abstract 8111.
* Savonije JH, van Groeningen CJ, Van Bochove A, Honkoop AH, van Felius CL, Wormhoudt LW, et al.Effects of early intervention with epoetin alfa on transfusion requirement, hemoglobin level and survival during platinum-based chemotherapy: Results of a multicenter randomised controlled trial. *European Journal of Cancer* 2005;**41**(11):1560–9.
Savonije JH, van Groeningen CJ, Wormhoudt LW, Giaccone G. Early Intervention with epoetin alfa during platinum-based chemotherapy: an analysis of quality-of- life results of a multicenter, randomized, controlled trial compared with population normative data. *The oncologist* 2006;**11**(2):197–205.

41. Smith 2008
20010103. Continuing reassessment of the risks of erythropoiesis-stimulating agents (ESAs) administered
for the treatment of anemia associated with cancer chemotherapy. Department of Health and Human Services, Food and Drugs Administration, Center
for Drug Evaluation and Research, Oncologic Drugs Advisory Committee, Rockville (MD), USA, 2007;
Vol. http://www.fda.gov/ohrms/dockets/ac/07/briefing/ 2007–4301b2–02– FDA.pdf.
Glaspy J, Smith RE, Aapro M, Ludwig H, Pinter T, Smakal M, et al.Results from a phase 3 randomized, double blind, placebo controlled study of darbepoetin alfa for
the treatment of anemia in cancer patients not receiving chemotherapy or radiotherapy. Haematologica 2007; Vol. 92, issue Suppl 1:136.
* Smith RE, Aapro MS, Ludwig H, Pinter T, Smakal M, Ciuleanu TE, et al.Darbepoetin Alfa for the Treatment of Anemia in Patients With Active Cancer Not Receiving Chemotherapy or Radiotherapy: Results of a Phase
III, Multicenter, Randomized, Double-Blind, Placebo- Controlled Study. *Journal of Clinical Oncology* 2008;**26**(7): 1040–50.

42. Strauss 2008
Strauss H, Haendgen G, Dunst.J, Koelbl H. Effects of anaemia correction with epoetin beta in patients with advanced cervical cancer. Proceedings of the American Society of Clinical Oncology 2003:abstract 5121.
* Strauss HG, Haendgen G, Dunst J, Haywrad CRW, Burger HU, Scherhag A, et al.Effects of anemia correction with epoetin beta in patients receiving radiochemotherapy for advanced cervical cancer. *International Journal of Gynecological Cancer* 2008;**18**(Suppl 3):515–24.

43. Taylor 2005
Taylor K, Ganly P, Charu V, DiBenedetto J, Kracht K, Rossi G, et al.Randomized, double-blind, placebo-controlled study of darbepoetin alfa every 3 weeks for the treatment of chemotherapy-induced anemia. Blood 2005; Vol. 106, issue 11:abstract 3556.

44. (X) Ten Bokkel Huinink 1998
Reed N, Rhan S, Hayward C, Burger H, Ten Bokkel Huinink W. Impact of epoetin beta on the survival of anemic patients with ovarian cancer receiving platinum- based chemotherapy.

Proceedings of the American Society of Clinical Oncology 2003; Vol. 23, issue 16s:abstract 5102.
* Ten Bokkel Huinink WW, De Swart CA, Van Toorn DW, Morack G, Breed WP, Hillen HF, et al.Controlled multicentre study of the influence of subcutaneous recombinant human erythropoietin on anaemia and transfusion dependency in patients with ovarian carcinoma treated with platinum-based chemotherapy. *Medical Oncology* 1998;**15**(3):174–82.

45. (X) Thatcher 1999
De Campos E, Radford J, Steward Milroy R, Dougal M, Swindell R, et al.Clinical and in vitro effects of recombinant human erythropoietin in patients receiving intensive chemotherapy for small-cell lung cancer. *Journal of Clinical Oncology* 1995;**13**(7):1623–31.
Thatcher N, De Campos ES, Bell DR, Steward WP, Varghese G, Morant R, et al.Epoetin alpha prevents anaemia and reduces transfusion requirements in patients undergoing primarily platinum-based chemotherapy for small cell lung cancer. *British Journal of Cancer* 1999;**80**(3-4):396–402.

46. Thomas 2002
Thomas H, McAdam KF, Thomas RJ, Joffe JK, Sugden EM, Awwad ST, et al.Early intervention with epoetin alfa for treatment of anaemia and improvement of quality of life in cancer patients undergoing myelotoxic chemotherapy. Annals of Oncology 2002; Vol. 13, issue Suppl 5:177.

47. (X) Thomas 2008
Luksenburg H, Weir A, Wager R. GOG-191: Safety concerns associated with Aranesp (darbepoetin alfa) Amgen Inc. and Procrit (epoetin alfa) Ortho Biotech L.P., for the treatment of Anemia associated with cancer chemotherapy. Department of Health and Human Services, Food and Drug Administration, Center for Drug Evaluation and Research, Oncologic Drugs Advisory Committee, Rockville (MD), USA, 2004; Vol. http://www.fda.gov/ohrms/dockets/ac/04/briefing/4037b2˙04.pdf [date of last access March 27, 2009].
Thomas G, Ali S, Hoebers FJP, Darcy KM, Rodgers
WH, Patel M, et al.Phase III trial to evaluate the efficacy of maintaining hemoglobin levels above 12.0 g/dL with erythropoietin vs above 10.0 g/dL without erythropoietin in anemic patients receiving concurrent radiation and cisplatin for cervical cancer. *Gynecologic Oncology* 2008;**108**(2): 317–25.

48. (X) Untch 2008
Untch M, Fascing PA, Bauernfeind I, Conrad U, Camara O, Fett W, et al.PREPARE trial. A randomized phase III trial comparing preoperative, dose-dense, dose-intensified chemotherapy with epirubicin, paclitaxel and CMF with a standard dosed epirubicin/cyclophosphamide followed by paclitaxel +/- darbepoetin alfa in primary breast cancer: A preplanned interim analysis of efficacy at surgery. Journal of Clinical Oncology 2008; Vol. 26, issue Suppl:abstract 517.

49. (X) Vadhan-Raj 2004
Vadhan-Raj S, Skibber JM, Crane C, Buesos-Ramos CE, Rodriguez-Bigas MA, Feig BW, et al.Randomized, double- blind, placebo-controlled trial of epoetin alfa (Procrit)
in patients with rectal and gastric cancer undergoing chemo-radiotherapy (CT/RT) followed by surgery: early termination of the trial due to increased incidence of thromboembolic events (TEE). Blood 2004; Vol. 104, issue 11:797a, abstract 2915.

50. (X) Vansteenkiste 2002
Kallich JD, Tchekmedyian NS, Damiano AM, Shi J, Black JT, Erder MH. Psychological outcomes associated with anemia-related fatigue in cancer patients. *Oncology (Huntingt)* 2002;**16**(9 Suppl 10):117–24.

Pirker R, Vansteenkiste J, Gateley J, Yates P, Colowick
A, Musil J. A phase 3, double-blind, placebo-controlled, randomized study of novel erythropoiesis stimulating protein (NESP) in patients undergoing platinum treatment for lung cancer. Proceedings of the American Society of Clinical Oncology 2001; Vol. 20:abstract 1572. Tchekmedyian NS, Kallich J, McDermott A, Fayers P, Erder MH. The relationship between psychologic distress and cancer-related fatigue. *Cancer* 2003;**98**(1):198–203.
Tchekmedyian S, Glaspy J, Colowick A, Tomita D, Rossi G. Effect of darbepoetin alfa and recombinant human erythropoietin (rHuEPO) on early hemoglobin (Hb) changes in anemic cancer patients (pts). Annals of Oncology 2002; Vol. 13, issue Suppl 5:184.
* Vansteenkiste J, Pirker R, Massuti B, Barata F, Font
A, Fiegl M, et al.Double-blind, placebo-controlled, randomized phase III trial of darbepoetin alfa in lung cancer patients receiving chemotherapy. *Journal of the National Cancer Institute* 2002;**94**:1211–20.
Vansteenkiste J, Poulsen E, Rossi G, Glaspy J. Darbepoetin alfa: impact on treatment for chemotherapy-induced anemia and considerations in special populations. *Oncology (Huntingt)* 2002;**16**(10 Suppl 11):45–55.
Vansteenkiste J, Tomita D, Rossi G, Pirker R. Darbepoetin alfa in lung cancer patients on chemotherapy: a retrospective comparison of outcomes in patients with mild versus moderate-to-severe anaemia at baseline. *Supportive Care in Cancer* 2004;**12**(4):253–62.

51. (X) Wilkinson 2006
Wilkinson PM, Antonopoulos M, Lahousen M, Lind M, Kosmidis P, Epo INT. Epoetin alfa in platinum- treated ovarian cancer patients: results of a multinational, multicentre, randomised trial. *British Journal of Cancer* 2006;**94**(7):947–54.

52. (X) Witzig 2005
Dicato M, Vercammen E, Liu KL, Xiu LX, Bowers P. Relationship of body weight to efficacy of a fixed-dose regimen of epoetin alfa vs placebo in anemic cancer patients. Haematologica 2005; Vol. 90, issue Suppl 2:abstract 0077. Witzig TE, Silberstein PT, Loprinzi CL, Sloan JA, Novotny PJ, Mailliard JA, et al.Phase III, randomized, double- blind study of epoetin alfa compared with placebo in anemic patients receiving chemotherapy. *Journal of Clinical Oncology* 2005;**23**(12):2606–17.

53. (X) Wright 2007
Luksenburg H, Weir A, Wager R. EPO-CAN-20: Safety concerns associated with Aranesp (darbepoetin alfa) Amgen Inc and Procrit (epoetin alfa) Ortho Biotech
L.P. for the treatment of anemia associated with cancer chemotherapy. Oncologic Drugs Advisory Committee, Department of Health and Human Services, Food and Drug Administration, Center for Drug Evaluation and Research, Rockville (MD), USA, 2004; Vol. http://www.fda.gov/ ohrms/dockets/ac/04/briefing/4037b2˙04.pdf [date of last access March 27, 2009].
Wright JR, Ung YC, Julian JA, Pritchard KI, Whelan, TJ, Smith C, et al.Randomized, double-blind, placebo- controlled trial of erythropoietin in non-small-cell lung cancer with disease-related anemia. *Journal of Clinical Oncology* 2007;**25**(9):1027–32.

Search strategies

Search strategies for IPD meta-analysis update

Database: Ovid MEDLINE(R)

. 1 exp ERYTHROPOIETIN/

. 2 exp ERYTHROPOIETIN, RECOMBINANT/

. 3 erythropoietin.mp.

. 4 erythropoiesis.mp.

. 5 exp EPOETIN ALFA/

. 6 epoetin.mp.

. 7 epo.mp.

. 8 epoetin alfa.mp.

. 9 epoetin beta.mp.

. 10 eprex.mp.

. 11 neorecormon.mp.

. 12 aranesp.mp.

. 13 procrit.mp.

. 14 recombinant erythropoietin.mp.

. 15 darbepoetin alfa.mp.

. 16 darbepoetin.mp.

17. RECEPTORS, ERYTHROPOIETIN/
18. CERA.mp.
19 or/1-18

20 exp ANEMIA/dt, th [Drug Therapy, Therapy]

21 anaemia.mp.

22 anemia.mp.

23 (anemi$ adj3 cancer).mp.

24 (anaemi$ adj3 cancer).mp.

25 or/20-24

26. exp Neoplasms/

27 malignan$.mp.

28. cancer$.mp.

29. oncolog$.tw.

30. myelodysplas$.tw.

31 chemotherapy.mp.

32 tumo?r$.mp.

33 carcinom$.mp.

34 or/26-33

35 19 and 25

36 34 and 25

. randomized controlled trial.pt. controlled clinical trial.pt. randomized controlled trials/

. random allocation/ double blind method/ single blind method/ or/37-42

. (ANIMALS not HUMANS).sh. 43 not 44

. clinical trial.pt.
exp clinical trials/
(clin$ adj25 trial$).ti,ab.
((singl$ or doubl$ or trebl$ or tripl$) adj25 (blind$ or mask$)).ti,ab.

. placebos/ placebo$.ti,ab. random$.ti,ab. research design/

. or/46-53 54 not 44 55 not 45

. comparative study/ exp evaluation studies/ follow up studies/

. prospective studies/

. (control$ or prospectiv$ or volunteer$).ti,ab. or/57-61

. 62 not 44
63 not (45 or 56) 45or56or64 36 and 65

.

Length: 1450 - 96 - 67 - 29*5 = 1209

GOOGLE SCHOLAR SEARCH

(erythropoietin OR erythropoiesis OR epoetin OR epo) (anemia OR anaemia) (malignant OR cancer OR oncology OR myelodysplasy OR chemotherapy OR tumor OR carcinoma) (random OR randomized OR randomised OR "clinical trial")

Length: 219

searched on 2013-04-09

Hits: 36.500

53 studies included

48 references for included

41 references found

6_boehm_green-tea

Reference: Boehm et al. [@the*cochrane*collaboration*green*2009]

Included studies

1. **(X) Bettuzzi 2006**
Bettuzzi S, Brausi M, Rizzi F, Castagnetti G, Peracchia G, Corti A. Chemoprevention of Human Prostate Cancer by Oral Administration of Green Tea Catechins in Volunteers with High-Grade Prostate Intraepithelial Neoplasia: A Preliminary Report from a One-Year Proof-of-Principle Study. *Cancer Research* 2006;**66**(2):1234.

2. **(X) Bonner 2005**
Bonner MR, Rothman N, Mumford JL, He X, Shen M, Welch R, et al.Green tea consumption, genetic susceptibility, PAH-rich smoky coal, and the risk of lung cancer. *Mutation Research* 2005;**582**(1-2):53–60.

3. **(X) Chyou 1993**
Chyou PH, Nomura AM, Stemmermann GN. A prospective study of diet, smoking, and lower urinary tract cancer. *Annals of Epidemiology* 1993;**3**(3):211–6.

4. **(X) Fujino 2002**
Fujino Y, Tamakoshi A, Ohno Y, Mizoue T, Tokui N, Yoshimura T. Prospective study of educational background and stomach cancer in Japan. *Preventive Medicine* 2002;**35**(2):121–7.

5. **(X) Galanis 1998**
Galanis DJ, Kolonel LN, Lee J, Nomura A. Intakes
of selected foods and beverages and the incidence of gastric cancer among the Japanese residents of Hawaii:
a prospective study. *International Journal of Epidemiology* 1998;**27**(2):173–80.

6. **(X) Gao 1994**
Gao YT, McLaughlin JK, Blot WJ, Ji BT, Dai Q, Fraumeni JF Jr. Reduced risk of esophageal cancer associated with green tea consumption. *Journal of the National Cancer Institute* 1994;**86**(11):855–8.

7. **(X) Goto 1990**
Goto R, Masuoka H, Yoshida K, Mori M, Miyake H. [A case control study of cancer of the pancreas]. *Gan No Rinsho* 1990;**Spec No**:344–50.

8. **(X) Hoshiyama 2002**
Hoshiyama Y, Kawaguchi T, Miura Y, Mizoue T, Tokui
N, Yatsuya H, et al.A prospective study of stomach cancer death in relation to green tea

consumption in Japan. *British Journal of Cancer* 2002;**87**(3):309–13.

9. **Huang 1999**
Huang X, Tajima K, Hamajima N, Inoue M, Takezaki T, Kuroishi T, et al.Effect of life styles on the risk of subsite- specific gastric cancer in those with and without family history. *Journal of Epidemiology* 1999;**9**(1):40–5.

10. **(X) Ide 2007**
Ide R, Fujino Y, Hoshiyama Y, Mizoue T, Kubo T, Pham TM, et al.A prospective study of green tea consumption and oral cancer incidence in Japan. *Annals of Epidemiology* 2007;**17**:821–6.

11. **(X) Inoue 1994**
Inoue M, Tajima K, Hirose K, Kuroishi T, Gao CM, Kitoh T. Life-style and subsite of gastric cancer--joint effect of smoking and drinking habits. *International Journal of Cancer* 1994;**56**(4):494–9.

12. **(X) Inoue 1998**
Inoue M, Tajima K, Hirose K, Hamajima N, Takezaki T, Kuroishi T, et al.Tea and coffee consumption and the risk of digestive tract cancers: data from a comparative case- referent study in Japan. *Cancer Causes & Control* 1998;**9**(2): 209–16.

13. **(X) Inoue 2008**
Inoue M, Robien K, Wang R, Van Den Berg J, Koh WP, Yu MC. Green tea intake, MTHFR/TYMS genotype and breast cancer risk: the Singapore Chinese Health Study. *Carcinogenesis* 2008;**29**(10):1967–72.

14. **(X) Ishikawa 2006**
Ishikawa A, Kuriyama S, Tsubono Y, Fukao A, Takahashi H, Tachiya H, et al.Smoking, alcohol drinking, green tea consumption and the risk of esophageal cancer in Japanese men. *Journal of Epidemiology* 2006;**16**(5):185–92.

15. **Ji 1996**
Ji BT, Chow WH, Yang G, McLaughlin JK, Gao RN, Zheng W, et al.The influence of cigarette smoking, alcohol, and green tea consumption on the risk of carcinoma of the cardia and distal stomach in Shanghai, China. *Cancer* 1996; **77**(12):2449–57.

16. **(X) Ji 1997**
Ji BT, Chow WH, Hsing AW, McLaughlin JK, Dai Q, Gao YT, et al.Green tea consumption and the risk of pancreatic and colorectal cancers. *International Journal of Cancer* 1997; **70**(3):255–8.

17. **(X) Jian 2007**
Jian L, Lee AH, Binns CW. Tea and lycopene protect against prostate cancer. *Asia Pacific Journal of Clinical Nutrition* 2007;**16**(Suppl 1):453–7.

18. **(X) Kato 1990a**
Kato I, Tominaga S, Matsuura A, Yoshii Y, Shirai M, Kobayashi S. A comparative case-control study of colorectal cancer and adenoma. *Japanese Journal of Cancer Research* 1990;**81**(11):1101–8.

19. **(X) Kato 1990b**
Kato I, Tominaga S, Ito Y, Kobayashi S, Yoshii Y, Matsuura A, et al.A comparative case-control analysis of stomach cancer and atrophic gastritis. *Cancer Research* 1990;**50**(20):

6559–64.

20. **(X) Key 1999**
Key TJ, Sharp GB, Appleby PN, Beral V, Goodman
MT, Soda M, et al.Soya foods and breast cancer risk: a prospective study in Hiroshima and Nagasaki, Japan. *British Journal of Cancer* 1999;**81**(7):1248–56.

21. **(X) Kikuchi 2006**
Kikuchi N, Ohmori K, Shimazu T, Nakaya N, Kuriyama S, Nishino Y, et al.No association between green tea and prostate cancer risk in Japanese men: the Ohsaki Cohort Study. *British Journal of Cancer* 2006;**95**(3):371–3.

22. **(X) Koizumi 2003**
Koizumi Y, Tsubono Y, Nakaya N, Nishino Y, Shibuya D, Matsuoka H, et al.No association between green tea and the risk of gastric cancer: pooled analysis of two prospective studies in Japan. *Cancer Epidemiology, Biomarkers & Prevention* 2003;**12**(5):472–3.

23. **(X) Kono 1988**
Kono S, Ikeda M, Tokudome S, Kuratsune M. A case- control study of gastric cancer and diet in northern Kyushu, Japan. *Japanese Journal of Cancer Research* 1988;**79**(10): 1067–74.

24. **(X) Kurahashi 2007**
Kurahashi N, Sasazuki S, Iwasaki M, Inoue M, Tsugane S. Green tea consumption and prostate cancer risk in Japanese men: a prospective study. *American Journal of Epidemiology* 2007;**167**(1):71–7.

25. **(X) Kuriyama 2006**
Kuriyama S, Shimazu T, Ohmori K, Kikuchi N, Nakaya N, Nishino Y, et al.Green tea consumption and mortality due to cardiovascular disease, cancer, and all causes in Japan. *Journal of the American Medical Association* 2006;**296**: 1255–65.

26. **(X) Li 2008**
Li Q, Kakizaki M, Kuriyama S, Sone T, Yan H, Nakaya N, et al.Green tea consumption and lung cancer risk: the Ohsaki study. *British Journal of Cancer* 2008;**99**:1179–84.

27. **(X) Lin 2008**
Lin Y, Kikuchi S, Tamakoshi A, Yagyu K, Obata Y, Kurosawa M, et al.Green tea consumption and the risk of pancreatic cancer in Japanese adults. *Pancreas* 2008;**37**(1): 25–30.

28. **(X) Luo 2007**
Luo J, Inoue M, Iwasaki M, Sasazuki S, Otani T, Ye W, et al.Green tea and coffee intake and risk of pancreatic cancer in a large-scale, population-based cohort study in japan (JPHC study). *European Journal of Cancer Prevention* 2007; **16**:542–8.

29. **(X) Mizuno 1992**
Mizuno S, Watanabe S, Nakamura K, Omata M, Oguchi H, Ohashi K, et al.A multi-institute case-control study on the risk factors of developing pancreatic cancer. *Japanese Journal of Clinical Oncology* 1992;**22**(4):286–91.

30. **(X) Mu 2003**
Mu LN, Zhou XF, Ding BG, Wang RH, Zhang ZF, Chen CW, et al.[A case-control study on drinking green tea and decreasing risk of cancers in the alimentary canal among cigarette smokers and alcohol drinkers]. *Zhonghua Liu Xing Bing Xue Za Zhi* 2003;**24**(3):192–5.

31. (X) Nagano 2001
Nagano J, Kono S, Preston DL, Mabuchi K. A prospective study of green tea consumption and cancer incidence, Hiroshima and Nagasaki (Japan). *Cancer Causes & Control* 2001;**12**(6):501–8.

32. (X) Nakachi 2000
Nakachi K, Matsuyama S, Miyake S, Suganuma M, Imai K. Preventive effects of drinking green tea on cancer and cardiovascular disease: epidemiological evidence for multiple targeting prevention. *Biofactors* 2000;**13**(1-4): 49–54.

33. (X) Sasazuki 2004
Sasazuki S, Inoue M, Hanaoka T, Yamamoto S, Sobue T, Tsugane S. Green tea consumption and subsequent risk of gastric cancer by subsite: the JPHC Study. *Cancer Causes & Control* 2004;**15**(5):483–91.

34. (X) Setiawan 2001
Setiawan VW, Zhang ZF, Yu GP, Lu QY, Li YL, Lu ML, et al.Protective effect of green tea on the risks of chronic gastritis and stomach cancer. *International Journal of Cancer* 2001;**92**(4):600–4.

35. (X) Song 2008
Song YJ, Kristal AR, Wicklund KG, Cushing-Haugen KL, Rossing MA. Coffee, tea, colas, and risk of epithelial ovarian cancer. *Cancer Epidemiology, Biomarkers & Prevention* 2008; **17**(3):712–6.

36. (X) Sonoda 2004
Sonoda T, Nagata Y, Mori M, Miyanaga N, Takashima N, Okumura K, et al.A case-control study of diet and prostate cancer in Japan: possible protective effect of traditional Japanese diet. *Cancer Science* 2004;**95**(3):238–42.

37. (X) Sun 2007
Sun CL, Yuan JM, Koh WP, Lee HP, Yu MC. Green tea and black tea consumption in relation to colorectal cancer risk: the Singapore Chinese Health Study. *Carcinogenesis* 2007; **28**(10): 2143–8.

38. (X) Suzuki 2004
Suzuki Y, Tsubono Y, Nakaya N, Suzuki Y, Koizumi Y, Tsuji I. Green tea and the risk of breast cancer: pooled analysis of two prospective studies in Japan. *British Journal of Cancer* 2004;**90**(7):1361–3.

39. (X) Suzuki 2005
Suzuki Y, Tsubono Y, Nakaya N, Koizumi Y, Suzuki Y, Shibuya D, et al.Green tea and the risk of colorectal cancer: pooled analysis of two prospective studies in Japan. *Journal of Epidemiology* 2005;**15**(4):118–24.

40. Tajima 1985
Tajima K, Tominaga S. Dietary habits and gastro-intestinal cancers: a comparative case-control study of stomach and large intestinal cancers in Nagoya, Japan. *Japanese Journal of Cancer Research* 1985;**76**(8):705–16.

41. (X) Tsubono 2001
Tsubono Y, Nishino Y, Komatsu S, Hsieh CC, Kanemura S, Tsuji I, et al.Green Tea and the Risk of Gastric Cancer in Japan. *New England Journal of Medicine* 2001;**344**(9): 632–6.

42. **(X) Wakai 2004**
Wakai K, Hirose K, Takezaki T, Hamajima N, Ogura
Y, Nakamura S, et al.Foods and beverages in relation to urothelial cancer: case-control study in Japan. *International Journal of Urology* 2004;**11**(1):11–9.

43. **(X) Wang 1999**
Wang M, Guo C, Li M. [A case-control study on the dietary risk factors of upper digestive tract cancer]. *Zhonghua Liu Xing Bing Xue Za Zhi* 1999;**20**(2):95–7.

44. **(X) Wang 2007**
Wang JM, Xu B, Rao JY, Shen HB, Xue HC, Jiang QW. Diet habits, alcohol drinking, tobacco smoking, green tea drinking, and the risk of esophageal squamous cell carcinoma in the Chinese population. *European Journal of Gastroenterology and Hepatology* 2007;**19**(2): 171–6.

45. **(X) Wu 2003**
Wu AH, Yu MC, Tseng CC, Hankin J, Pike MC. Green tea and risk of breast cancer in Asian Americans. *International Journal of Cancer* 2003;**106**(4):574–9.

46. **(X) Yang 2007**
Yang G, Shu XO, Li H, Chow WH, Ji BT, Zhang X, et al.Prospective cohort study of green tea consumption and colorectal cancer risk in women. *Cancer Epidemiology, Biomarkers & Prevention* 2007;**16**(6):1219–23.

47. **(X) Ye 1998**
Ye WM, Yi YN, Luo RX, Zhou TS, Lin RT, Chen GD. Diet and gastric cancer: a case-control study in Fujian Province, China. *World Journal of Gastroenterology* 1998;**4**(6):516–8.

48. **(X) Yu 1995**
Yu GP, Hsieh CC, Wang LY, Yu SZ, Li XL, Jin TH. Green- tea consumption and risk of stomach cancer: a population- based case-control study in Shanghai, China. *Cancer Causes & Control* 1995;**6**(6):532–8.

49. **(X) Zhang 2002**
Zhang M, Binns CW, Lee AH. Tea consumption and ovarian cancer risk: a case-control study in China. *Cancer Epidemiology, Biomarkers & Prevention* 2002;**11**(8):713–8.

50. **(X) Zhang 2007**
Zhang M, Holman CDAJ, Huang JP, Xie X. Green tea and the prevention of breast cancer: a case-control study in Southeast China. *Carcinogenesis* 2007;**28**(5):1074–8.

51. **(X) Zhong 2001**
Zhong L, Goldberg MS, Gao YT, Hanley JA, Parent
ME, Jin F. A population-based case-control study of lung cancer and green tea consumption among women living in Shanghai, China. *Epidemiology* 2001;**12**(6):695–700.

Ovid Search Strategy

Ovid Search Strategy modified for MEDLINE, EMBASE, Amed and PsychInfo Last searched on 13/01/09

1. green tea.ti,ab,rw,sh.

2. camellia sinensis.ti,ab,rw,sh.
3. tea.ti,ab,rw,sh.
4. thea.ti,ab,rw,sh.
5. Gruner Tee.ti,ab,rw,sh.
6. matsu-cha.ti,ab,rw,sh.
7. mattsu-cha.ti,ab,rw,sh.
8. antiox$.mp.
9. anti-oxid$.mp.
10. 1or2or3or4or5or6or7or8or9
11. tumour.ti,ab,rw,sh.
12. cancer$.ti,ab,rw,sh.
13. oncol$.ti,ab,rw,sh.
14. malignant.ti,ab,rw,sh.
15. survival$.mp.
16. mortality$.mp.
17. 11or12or13or14or15or16
18. trial$.ti,ab,rw,sh.
19. study.ti,ab,rw,sh.
20. cohort$.mp.
21. exp Cohort studies/
22. exp Clinical trials/
23. exp Clinical trial/
24. ((prospectiv$ or observation$) adj5 (research$ or data$ or stud$)).mp.
25. longitud$.mp.
26. 18or19or20or21or22or23or24or25
27. 10 and 17 and 26

Length: 726 - 94 - 175 = 605

GOOGLE SCHOLAR SEARCH

("green tea" OR "camellia sinensis" OR "Gruner Tee" OR matsu-cha OR mattsu-cha) (tumor OR cancer OR oncological OR malignant OR survival OR mortality) (trial OR study OR cohort OR "observational study")

length: 218

searched on 2013-04-09

Hits: 17.900

51 references/studies included

48 references found

7_guimaraes_incentive-spirometry

Reference: Guimarães et al. [@the*cochrane*collaboration*incentive*2009]

Included studies

1. **(X) Celli 1984**
   [*] Celli BR, Rodriguez K, Snider GL. A controlled trial of intermittent positive pressure breathing incentive spirometry and deep breathing exercises in preventing pulmonary complications after abdominal surgery. *The American Review of Respiratory Disease* 1984;**130**:12–5. [MEDLINE: 6377994]

2. **(X) Craven 1974**
   [*] Craven JL, Evans GA, Davenport PJ, Williams RH. The evaluation of the incentive spirometer in the management of postoperative pulmonary complications. *The British Journal of Surgery* 1974;**61**:793–7. [MEDLINE: 4416262]

3. **(X) Dohi 1978**
   [*] Dohi S, Gold MI. Comparison of two methods of postoperative respiratory care. *Chest* 1978;**73**(5):592–5.

4. **(X) Hall 1991**
   [*] Hall JC, Tarala R, Harris J, Tapper J, Christiansen K. Incentive spirometer versus routine chest physiotherapy for prevention of pulmonary complications after abdominal surgery. *Lancet* 1991;**337**:953–5. [MEDLINE: 1678039]

5. **(X) Hall 1996**
   [*] Hall JC, Tarala RA, Tapper J, Hall, JL. Prevention of respiratory complications after abdominal surgery: a randomised clinical trial. *BMJ* 1996;**312**:148–52. [MEDLINE: 8563533]

6. **(X) Jung 1980**
   [*] Jung R, Wight J, Nusser R, Rosoff L. Comparison of three methods of respiratory care following upper abdominal surgery. *Chest* 1980;**78**(1):31–5.

7. **(X) Lyager 1979**
   [*] Lyager S, Wernberg M, Rajani N, Boggild-Madsen B, Nielsen L, Nielsen HC, et al.Can postoperative pulmonary conditions be improved by treatment with the Bartlett- Edwards incentive spirometer after upper abdominal surgery?. *Acta Anaesthesiologica Scandinavica* 1979;**23**(4): 312–9.

8. **(X) O'Connor 1988**
   [*] O'Connor M, Tattersall MP, Carter JA. An evaluation of the incentive spirometer to improve lung function after cholecystectomy. *Anaesthesia* 1988;**43**(9):785–7.

9. **(X) Ricksten 1986**
   [*] Ricksten SE, Bengtsson A, Soderberg C, Thorden M, Kvist H. Effects of periodic positive airway pressure by mask on postoperative pulmonary function. *Chest* 1986;**89**(6): 774–81.

10. **(X) Schwieger 1986**

* Schwieger I, Gamulin Z, Forster A, Meyer P, Gemperle M, Suter PM. Absence of benefit of incentive spirometry in low-risk patients undergoing elective cholecystectomy. A controlled randomized study. *Chest* 1986;**89**(5):652–6.

11. **(X) Stock 1985**
* Stock MC, Downs JB, Gauer PK, Alster JM, Imrey PB. Prevention of postoperative pulmonary complications with CPAP, incentive spirometry, and conservative therapy. *Chest* 1985;**87**(2):151–7.

Search strategy for MEDLINE

#1 explode spirometry/ all subheadings
#2 (incentiv* near spiromet?r*) or (incentiv* spiromet?r*)
#3 spiromet?r in TI, AB
#4 explode breathing exercises/ all subheadings
#5 (breath* exercis*) or (breath* near exercis*)
#6 #1 or #2 or #3 or #4 or #5
#7 explode Bronchial Spasm/ all subheadings
#8 explode Respiratory Distress Syndrome, Adult/ all subheadings
#9 explode Atelectasis/ all subheadings
#10 explode Pneumonia/ all subheadings
#11 ((lung or pulmonary) complication) or ((lung or pulmonary) near complication*)
#12 Tracheo?bronchial
#13 bronchospasm
#14 breath near (inadequacy or insufficiency or failure)
#15 #7 or #8 or #9 or #10 or #11 or #12 or #13 or #14
#16 #6 and #15
#17 explode adult/ all subheadings
#18 (explode "Infant-+" / all SUBHEADINGS in MIME,MJME) or (explode "Adolescent-" / all SUBHEADINGS in MIME,MJME) or (explode "Child-+" / all SUBHEADINGS in MIME,MJME)
#19 #18 not (#18 and #17)
#20 #16 not #19

Length: 933 - 9*3 - 11*4 = 862

GOOGLE SCHOLAR SEARCH

"incentive spirometry" OR "incentive spirometer" OR "breathing exercise" OR "breath exercise"

Length: 93

searched on 2013-04-10

Hits: 13.400

11 references/studies included

11 references found

8_verbeek_occupational-hearing-loss

Reference: Verbeek et al. [@the*cochrane*collaboration*interventions*2012]

Included studies

1. **(X) Adera 1993**
   Adera T, Donahue AM, Malit BD, Gaydos JC. An epidemiologic method for assessing the effectiveness of hearing conservation programs using audiometric data. *Military Medicine* 1993;**158**(11):698–701.

2. **(X) Adera 2000**
   Adera T, Amir C, Anderson L. Use of comparison populations for evaluating the effectiveness of hearing loss prevention programs. *American Industrial Hygiene Association Journal* 2000;**61**(1):11–5.

3. **(X) Berg 2009**
   Berg RL, Pickett W, Fitz-Randolph M, Broste SK, Knobloch MJ, Wood DJ, et al.Hearing conservation program for agricultural students: short-term outcomes from a cluster-randomised trial with planned long-term follow-up. *Prev.Med*. 2009;**49**(6):546–52.
   Marlenga B, Linneman JG, Pickett W, Wood DJ, Kirkhorn SR, Broste SK, et al.Randomized trial of a hearing conservation intervention for rural students: Long-term outcomes. *Pediatrics* 2011;**128**(5):e1139–46.

4. **(X) Brink 2002**
   Brink LL, Talbott EO, Burks JA, Palmer CV. Changes over time in audiometric thresholds in a group of automobile stamping and assembly workers with a hearing conservation program. *American Industrial Hygiene Association Journal* 2002;**63**(4):482–7.

5. **(X) Davies 2008**
   Davies H, Marion S, Teschke K. The impact of hearing conservation programs on incidence of noise-induced hearing loss in Canadian workers. *American Journal of Industrial Medicine* 2008;**51**:923–31.

6. **(X) Erlandsson 1980**
   Erlandsson B, Hakanson H, Ivarsson A, Nilsson P. The difference in protection efficiency between earplugs and earmuffs. An investigation performed at a workplace. *Scandinavian Audiology* 1980;**9**(4):215–21.

7. **(X) Gosztonyi 1975**
   Gosztonyi RE. The effectiveness of hearing protective devices. *Journal of Occupational Medicine* 1975;**17**(9): 569–80.

8. **(X) Hager 1982**
Hager WL, Hoyle ER, Hermann ER. Efficacy of enforcement in an industrial hearing conservation program. *American Industrial Hygiene Association Journal* 1982;**43**(6): 455–65.

9. **(X) Heyer 2011**
Heyer N, Morata TC, Pinkerton LE, Brueck SE, Stancescu D, Panaccio MP, et al.Use of historical data and a novel metric in the evaluation of the effectiveness of hearing conservation program components. *Occup.Environ.Med.* 2011;**68**(7):510–7.

10. **(X) Horie 2002**
Horie S. Improvement of occupational noise-induced temporary threshold shift by active noise control earmuff and bone conduction microphone. *Journal of Occupational Health* 2002;**44**(6):414–20.

11. **(X) Joy 2007**
Joy G, Middendorf PJ. Noise exposure and hearing conservation in US coal mines, a surveillance report. *Journal of Occupational and Environmental Hygiene* 2007;**4**:26–35.

12. **(X) Lee-Feldstein 1993**
Lee-Feldstein A. Five-year follow-up study of hearing loss at several locations within a large automobile company. *American Journal of Industrial Medicine* 1993;**24**(1):41–54.

13. **(X) Meyer 1993**
Meyer GD, Wirth DB. An evaluation of the U.S. Air Force's detailed follow-up audiometric examination program. *Military Medicine* 1993;**158**(9):603–5.

14. **(X) Muhr 2006**
Muhr P, Månsson B, Hellström PA. A study of hearing changes among military conscripts in the Swedish army. *International Journal of Audiology* 2006;**45**:247–51.

15. **(X) Nilsson 1980**
Nilsson R, Lindgren F. The effect of long term use of hearing protectors in industrial noise. *Scandinavian Audiology* 1980; **Suppl 12**:204–11.

16. **(X) Pääkkönen 1998**
Pääkkönen R, Lehtomaki K, Savolainen S. Noise attenuation of communication hearing protectors against impulses from assault rifle. *Military Medicine* 1998;**163**(1): 40–3.

17. **(X) Pääkkönen 2001**
Pääkkönen R, Kuronen P, Korteoja M. Active noise reduction in aviation helmets during a military jet trainer test flight. *Scandinavian Audiology Supplementum* 2001;**52**: 177–9.

18. **(X) Park 1991a instructions**
Park MY, Casali JG. A controlled investigation of in-field attenuation performance of selected insert, earmuff, and canal cap hearing protectors. *Human Factors* 1991;**33**(6): 693–714.

19. **[same reference as above, excluded from count] Park 1991b protection**
Park MY, Casali JG. A controlled investigation of in-field attenuation performance of selected insert, earmuff, and canal cap hearing protectors. *Human Factors* 1991;**33**(6): 693–714.

20. **(X) Pell 1973**
Pell S. An evaluation of hearing conservation program - a five-year longitudinal study. *American Industrial Hygiene Association Journal* 1973;**34**(2):82–91.

21. **(X) Rabinowitz 2011**
Rabinowitz PM, Galusha D, Kirsche SR, Cullen MR, Slade MD, Dixon-Ernst C. Effect of daily noise exposure monitoring on annual rates of hearing loss in industrial workers. *Occupational and Environmental Medicine* 2011; **68**(6):414–8.

22. **(X) Reynolds 1990a**
Reynolds JL, Royster LH, Pearson RG. Hearing conservation programs (HCPs): the effectiveness of one company's HCP in a 12-hr work shift environment. *American Industrial Hygiene Association Journal* 1990;**51**(8):437–46.

23. **(X) Royster 1980**
Royster LH. An evaluation of the effectiveness of two different insert types of ear protection in preventing TTS in an industrial environment. *American Industrial Hygiene Association Journal* 1980;**41**(3):161–9.

24. **(X) Seixas 2011**
Seixas NS, Neitzel R, Stover B, Sheppard L, Daniell B, Edelson J. A multi-component intervention to promote hearing protector use among construction workers. International Journal of Audiology 2011; Vol. 50:s46–s56.

25. **(X) Simpson 1994**
Simpson TH, Stewart M, Kaltenbach JA. Early indicators of hearing conservation program performance. *Journal of the American Academy of Audiology* 1994;**5**:300–6.

PubMed Search

2009

#1 noise [tiab] AND (reduction [tiab] OR abatement [tiab] OR diminishment [tiab] OR elimination [tiab] OR "engineering controls" [tiab] OR "administrative controls"[tiab])
#2 "hearing loss prevention" [tiab] OR "hearing conservation" [tiab] OR "hearing surveillance" [tiab]
#3 "ear protective device" [tiab] OR "ear protective devices" [tiab] OR "hearing pro- tective device" [tiab] OR "hearing protec- tive devices" [tiab] OR "hearing protector" [tiab] OR "hearing protectors" [tiab] OR "hearing protection" [tiab] OR "ear muffs" [tiab] OR "ear plugs" [tiab] OR "ear de- fenders" [tiab]
#4 ("noise reduction" [tiab] AND "protec- tive equipment" [tiab])
#5 "Noise, Occupational/prevention and control"[Mesh]
#6 "Noise, Occupational"[Mesh]
#7 "protective equipment" [tiab]
#8 #6 AND #7
#9 #1 OR #2 OR #3 #4 OR #5 OR #8
#10 (effect*[tiab] OR control*[tiab] OR eval- uation*[tiab] OR program*[tiab]) AND (work*[tiab] OR worker*[tiab] OR work- place*[tiab] OR occupation*[tiab] OR pre- vention*[tiab] OR protect*[tiab])
#11 #9 AND #10

2012

#12 2008:2012[dp]
#13 #11 AND #12

Length: 1032 - 93 - 24 = 997

GOOGLE SCHOLAR SEARCH

"noise reduction" OR "hearing loss prevention" OR "hearing conservation" OR "hearing protection" OR "ear protection" OR "protective device" OR "protective equipment" OR "hearing protector" OR "ear muffs" OR "ear plugs"

Length: 219

searched on 2013-04-10

Hits: 19.500

25 studies included

24 references included

24 references found

9_yip_induction-anesthesia-children

Reference: `Yip et al. [@the`*`cochrane`*`collaboration`*`non-pharmacological`*`2009]`

Included Studies

1. **(X) Arai 2007**
   Arai YC, Ito H, Kandatsu N, Kurokawa S, Kinugasa S, Komatsu T. Parental presence during induction enhances the effect of oral midazolam on emergence behavior of children undergoing general anaesthesia. *Acta Anaesthesiologica Scandinavica* 2007;**51**:858–61. [PUBMED: 17578463]

2. **(X) Bevan 1990**
   Bevan JC, Johnston C, Haig MJ, Tousignant G, Lucy S, Kirnon V, et al.Preoperative parental anxiety predicts behavioural and emotional responses to induction of anaesthesia in children. *Canadian Journal of Anaesthesia* 1990;**37**:177–82. [PUBMED: 2311148]

3. **(X) Calipel 2005**
   * Calipel S, Lucas-Polomeni MM, Wodey E, Ecoffey C. Premedication in children: hypnosis versus midazolam. *Pediatric Anesthesia* 2005;**15**:275–81. [PUBMED: 15787917]

4. **Campbell 2005**
   * Campell C, Hosey MT, McHugh S. Facilitating coping behavior in children prior to dental general anesthesia: a randomized controlled trial. *Pediatric Anesthesia* 2005;**15**: 831–8. [PUBMED: 16176310]

5. **(X) Kain 1996**

Kain Z, Mayes L, Caramico L, Silver D, Spieker M, Nygren M, et al.Parental presence during induction of anesthesia: a randomized controlled trial. *Anesthesiology* 1996;**84**: 1060–7. [PUBMED: 8623999]

6. **(X) Kain 1998**
Kain ZN, Mayes LC, Wang SM, Caramico LA, Hofstadter MB. Parental presence during induction of anesthesia versus sedative premedication: which intervention is more effective?. *Anesthesiology* 1998;**89**(5):1147–56. [PUBMED: 9822003]

7. **(X) Kain 2000**
Kain ZN, Mayes LC, Wang SM, Caramico LA, Krivutza DM, Hofstadter MB. Parental presence and a sedative premedication for children undergoing surgery. *Anesthesiology* 2000;**92**:939–46. [PUBMED: 10754612]

8. **(X) Kain 2001**
Kain ZN, Wang SM, Mayes LC, Krivutza DM, Teague BA. Sensory stimuli and anxiety in children undergoing surgery: a randomized, controlled trial. *Anesthesia and Analgesia* 2001;**92**:897–903. [PUBMED: 11273921]

9. **(X) Kain 2003**
Kain ZN, Caldwell-Andrews AA, Mayes LC, Wang SM, Krivutza DM, LoDolce ME. Parental presence during induction of anesthesia. *Anesthesiology* 2003;**98**:58–64. [PUBMED: 12502980]

10. **(X) Kain 2004**
Kain ZN, Caldwell-Andrews AA, Krivutza DM, Weinberg ME, Gaal D, Wang SM, et al.Interactive music therapy as a treatment for preoperative anxiety in children: a randomized controlled trial. *Anesthesia and Analgesia* 2004; **98**:1260–6. [PUBMED: 15105197]

11. **(X) Kain 2007**
Kain ZN, Caldwell-Andrews AA, Mayes LC, Weinberg ME, Wang SM, MacLaren JE, et al. Family-centered preparation for surgery improves perioperative outcomes in children: a randomized controlled trial. *Anesthesiology* 2007;**106**: 65–74. [PUBMED: 17197846]

12. **(X) McEwen 2007**
McEwen A, Moorthy C, Quantock C, Rose H, Kavanagh R. The effect of videotaped preoperative information on parental anxiety during anesthesia induction for elective pediatric procedures. *Paediatr Anaesth* 2007;**17**(6):534–9. [PUBMED: 17498014]

13. **(X) Palermo 2000**
Palermo TM, Tripi PA, Burgess E. Parental presence during anaesthesia induction for outpatient surgery of the infant. *Paediatric Anaesthesia* 2000;**10**:487–91. [PUBMED: 11012951]

14. **(X) Patel 2006**
Patel A, Schieble T, Davidson M, Tran MC, Schoenberg C, Delphin E, et al.Distraction with a hand-held video game reduces pediatric preoperative anxiety. *Pediatric Anesthesia* 2006;**16**:1019–26. [PUBMED: 16972829]

15. **(X) Vagnoli 2005**
Vagnoli L, Caprilli S, Robiglio A, Messeri A. Clown doctors as a treatment for preoperative anxiety in children: a randomized, prospective study. *Pediatrics* 2005;**116**:563–7. [PUBMED: 16199685]

16. (X) Wang 2004
* Wang SM, Maranets I, Weinberg ME, Caldwell-Andrews AA, Kain ZN. Parental auricular acupuncture as an adjunct for parental presence during induction of anesthesia. *Anesthesiology* 2004;**100**:1399–404. [PUBMED: 15166558]

17. (X) Zuwala 2001
Zuwala R, Barber KR. Reducing anxiety in parents before and during pediatric anesthesia induction. *AANA Journal* 2001;**69**:21–5. [PUBMED: 11759134]

MEDLINE search strategy

#1. exp Anesthesia (MESH)
#2. an?esthe$.ti, ab
3. induc$.ti, ab
#4. exp Anxiety (MESH)
#5. exp Stress, Psychological (MESH)
6. distress$.ti,ab
7. distract$.ti,ab
8. cooperat$.ti,ab
9. exp Preoperative Care (MESH)
10. preoperat$.ti,ab
11. exp Child (MESH)
12. exp Adolescent (MESH)
13. #1 or #2 or #3
#14. #4 or #5 or #6 or #7 or #8
#15. #9 or #10
#16. #11 or #12
#17. #13 and #14 and #15 and #16

Length: 416 - 93 - 84 = 357

GOOGLE SCHOLAR SEARCH

((anesthesia OR anaesthesia) (child OR children OR adolescent) (anxiety OR stress OR distress OR "emergence behavior") ("preoperative care" OR distraction OR cooperation OR "parental presence"))

Length: 194

searched on 2013-04-10

Hits 29,300

17 references/studies included

16 references found

10_okebe_iron-children-malaria

Reference: `Okebe et al. [@the`*`cochrane`*`collaboration`*`oral`*`2011]`

References to studies included in this review

1. **(X) Adam 1997 (C)** ***{unpublished data only}***
   * Adam C. Iron supplementation and malaria. A randomized placebo-controlled field trial in rural Ethiopia. PhD thesis, London School of Tropical Medicine and Hygiene, London, UK.

2. **(X) Agarwal 2003**
   Agarwal KN, Gomber S, Bisht H, Som M. Anemia prophylaxis in adolescent school girls by weekly or daily iron-folate supplementation. *Indian Pediatrics* 2003;**40**(4): 296–301.

3. **(X) Aggarwal 2005**
   Aggarwal D, Sachdev HPS, Mallika V, Singh T. Iron supplementation in breast fed, full term, low birth weight infants. Cited in: Gera 2002 (Gera T, Sachdev HP. Effect of iron supplementation on incidence of infectious illness in children: systematic review. BMJ 2002;325(7373):1142) 1999. [: Gera 2002 (Additional references)]
   * Aggarwal D, Sachdev HPS, Nagpal J, Singh T, Mallika V. Hematological effect of iron supplementation in breastfed term low birth weight infants. *Archives of Disease in Childhood* 2005;**90**(1):26–9.

4. **(X) Aguayo 2000** ***{published data only (unpublished sought but not used)}*** Aguayo VM. School-administered weekly iron supplementation - effect on the growth and hemoglobin status of non-anemic Bolivian school-age children. Arandomized placebo-controlled trial. *European Journal of Nutrition* 2000;**39**(6):263–9.

5. **(X) Akenzua 1985**
   Akenzua GI, Ihongbe JC, Imasuen IW. Haemopoietic response of Nigerian village children to iron, folate supplementation and malaria prophylaxis. *Journal of Tropical Pediatrics* 1985;**31**(1):59–62.

6. **(X) Angeles 1993**
   Angeles IT, Schultink WJ, Matulessi P, Gross R, Sastroamidjojo S. Decreased rate of stunting among anemic Indonesian preschool children through iron supplementation. *The American Journal of Clinical Nutrition* 1993;**58**(3):339–42.

7. **(X)Ayoya 2009**
   Ayoya MA, Spiekermann-Brouwer GM, Traoré AK, Stoltzfus RJ, Habicht JP, Garza C. Multiple micronutrients including iron are not more effective than iron alone for improving hemoglobin and iron status of Malian school children. *The Journal of Nutrition* 2009;**139**(10): 1972–9.

8. **(X) Baqui 2003**
   Baqui AH, Walker CL, Zaman K, El Arifeen S, Chowdhury HR, Wahed MA, et al.Weekly iron supplementation does not block increases in serum zinc due to weekly zinc

supplementation in Bangladeshi infants. *The Journal of Nutrition* 2005;**135**(9):2187–91.
* Baqui AH, Zaman K, Persson LA, El Arifeen S, Yunus M, Begum N, et al.Simultaneous weekly supplementation of iron and zinc is associated with lower morbidity due to diarrhea and acute lower respiratory infection in Bangladeshi infants. *The Journal of Nutrition* 2003;**133**(12):4150–7. Black MM, Baqui AH, Zaman K, Ake Persson L, El Arifeen S, Le K, et al.Iron and zinc supplementation promote motor development and exploratory behavior among Bangladeshi infants. *The American Journal of Clinical Nutrition* 2004;**80** (4):903–10. Fischer Walker CL, Baqui AH, Ahmed S, Zaman
K, El Arifeen S, Begum N, et al.Low-dose weekly supplementation of iron and/or zinc does not affect growth among Bangladeshi infants. *European Journal of Clinical Nutrition* 2009;**63**(1):87–92.

9. **(X) Berger 1997**
Berger J, Aguayo VM, Tellez W, Lujan C, Traissac P, San Miguel JL. Weekly iron supplementation is as effective as 5 day per week iron supplementation in Bolivian school children living at high altitude. *European Journal of Clinical Nutrition* 1997;**51**(6):381–6.

10. **(X) Berger 2000**
* Berger J, Dyck JL, Galan P, Aplogan A, Schneider D, Traissac P, et al.Effect of daily iron supplementation on iron status, cell-mediated immunity, and incidence of infections in 6-36 month old Togolese children. *European Journal of Clinical Nutrition* 2000;**54**(1):29–35. Chippaux JP, Schneider D, Aplogan A, Dyck JL, Berger
J. Effects of iron supplementation on malaria infection [Effets de la supplementation en fer sur l'infection palustre]. *Bulletin de la Societe de Pathologie Exotique et de ses Filiales* 1991;**84**(1):54–62.
Schneider D, Chippaux JP, Aplogan A, Dyck JL, Berger J. Evaluation of the impact of iron treatment. Interference of malaria [Evaluation de l'impact d'un traitement martial. Interference du paludisme.]. *Bulletin de la Societe de Pathologie Exotique et de ses Filiales* 1995;**88**(5):260–4.

11. **(X) Berger 2006** ***{published data only (unpublished sought but not used)}*** Berger J, Ninh NX, Khan NC, Nhien NV, Lien DK,
Trung NQ, et al.Efficacy of combined iron and zinc supplementation on micronutrient status and growth in Vietnamese infants. *European Journal of Clincal Nutrition* 2006;**60**(4):443–54.

12. **(X) Bhatia 1993 (C)**
Bhatia D, Seshadri S. Growth performance in anemia and following iron supplementation. *Indian Pediatrics* 1993;**30** (2):195–200.

13. **(X) Charoenlarp 1973**
Charoenlarp P. Effect of iron and folate supplementation on haematocrit levels of school children in a rural area of Central Thailand. *Southeast Asian Journal of Tropical Medicine and Public Health* 1973;**4**(4):588–92.

14. **(X) Chwang 1988**
* Chwang LC, Soemantri AG, Pollitt E. Iron supplementation and physical growth of rural Indonesian children. *The American Journal of Clinical Nutrition* 1988; **47**(3):496–501. Soemantri AG, Pollitt E, Kim I. Iron deficiency anemia and educational achievement. *The American Journal of Clinical Nutrition* 1985;**42**(6):1221–8.

15. **(X) de Silva 2003**
Atukorala S, de Silva A, Ahluwalia N. Evaluation of the iron status of children in the presence of infections: effects of iron supplementation on iron status infection and morbidity. International Nutritional Anaemia Consultative Group Symposium. Hanoi, Vietnam 2001.

* de Silva A, Atukorala S, Weerasinghe I, Ahluwalia N. Iron supplementation improves iron status and reduces morbidity in children with or without upper respiratory tract infections: a randomized controlled study in Colombo, Sri Lanka. *The American Journal of Clinical Nutrition* 2003; **77**(1):234–41.

16. **(X) Desai 2003**
* Desai MR, Mei JV, Kariuki SK, Wannemuehler KA, Phillips-Howard PA, Nahlen BL, et al.Randomized, controlled trial of daily iron supplementation and intermittent sulfadoxine-pyrimethamine for the treatment of mild childhood anemia in western Kenya. *Journal of Infectious Diseases* 2003;**187**(4):658–66.
Terlouw DJ, Desai MR, Wannemuehler KA, Kariuki SK, Pfeiffer CM, Kager PA, et al.Relation between the response to iron supplementation and sickle cell hemoglobin phenotype in preschool children in western Kenya. *The American Journal of Clinical Nutrition* 2004;**79**(3):466–72.

17. **(X) Devaki 2007**
Devaki PB, Chandra RK, Geisser P. Effect of oral supplementation with iron(III)-hydroxide polymaltose complex on the immunological profile of adolescents with varying iron status. *Arzneimittel-Forschung (Drug Research)* 2007;**57**(6A):417-25.

18. **Dossa 2001a**
Dossa RA, Ategbo EA, de Koning FL, van Raaij JM, Hautvast JG. Impact of iron supplementation and deworming on growth performance in preschool Beninese children. *European Journal of Clinical Nutrition* 2001;**55**(4): 223–8.

19. **(X) Dossa 2001b**
Dossa RA, Ategbo EA, Van Raaij JM, de Graaf C, Hautvast JG. Multivitamin-multimineral and iron supplementation did not improve appetite of young stunted and anemic Beninese children. *The Journal of Nutrition* 2001;**131**(11): 2874–9.

20. **Fahmida 2007**
Fahmida U, Rumawas JS, Utomo B, Patmonodewo S, Schultink W. Zinc-iron, but not zinc-alone supplementation, increased linear growth of stunted infants with low haemoglobin. *Asia Pacific Journal of Clinical Nutrition* 2007;**16**(2): 301–9.

21. **(X) Gara 2010**
Gara SN, Madaki AJK, Thacher TD. A comparison of iron and folate with folate alone in hematologic recovery of children treated for acute malaria. *American Journal of Tropical Medicine and Hygiene* 2010;**83**(4):843–7.

22. **Gebresellassie 1996**
Gebreselassie H. Iron supplementation and malaria infection: results of a randomized controlled field trial. PhD dissertation. McGill University, Montreal, Quebec, Canada 1996.

23. **(X) Gopaldas 1983**
Gopaldas T, Raghavan R, Kanani S. Nutritional impact of anti-parasitic drugs, prophylactic vitamin A and iron- folic acid on underprivileged school girls in India. *Nutrition Research* 1983;**3**:831–44.

24. **(X) Gopaldas 1985**
* Gopaldas T, Kale M. Prophylactic iron supplementation for underprivileged school boys. I. Two levels of dosing and efficacy of teacher-distributions. *Indian Pediatrics* 1985;**22** (10): 731–6.

Gopaldas T, Kale M, Bhardwaj P. Prophylactic iron supplementation for underprivileged school boys. III. Impact on submaximal work capacity. *Indian Pediatrics* 1985;**22**(10):745–52.
Gopaldas T, Kale T, Bhardwaj P. Prophylactic iron supplementation for underprivileged school boys. II. Impact on selected tests of cognitive function. *Indian Pediatrics* 1985;**22**(10): 737–43.

25. (X) Greisen 1986 (C)
* Greisen G. Mild anaemia in African school children: effect on running performance and an intervention trial. *Acta Paediatrica Scandinavica* 1986;**75**(4):662–7.

26. (X) Hall 2002 (C)
Hall A, Roschnik N, Ouattara F, Touré I, Maiga F, Sacko M, et al.A randomised trial in Mali of the effectiveness of weekly iron supplements given by teachers on the haemoglobin concentrations of schoolchildren. *Public Health Nutrition* 2002;**5**(3):413–8.

27. Harvey 1989
Harvey PW, Heywood PF, Nesheim MC, Galme K, Zegans M, Habicht JP, et al.The effect of iron therapy on malarial infection in Papua New Guinean schoolchildren. *American Journal of Tropical Medicine and Hygiene* 1989;**40**(1):12–8.

28. (X) Hess 2002
Hess SY, Zimmermann MB, Adou P, Torresani T, Hurrell RF. Treatment of iron deficiency in goitrous children improves the efficacy of iodized salt in Cote d'Ivoire. *The American Journal of Clinical Nutrition* 2002;**75**(4):743–8.

29. (X) Hettiarachchi 2008 (C) Hettiarachchi M, Liyanage C, Wickremasinghe R, Hilmers DC, Abrams SA. The efficacy of micronutrient supplementation in reducing the prevalence of anaemia and deficiencies of zinc and iron among adolescents in Sri Lanka. *European Journal of Clinical Nutrition* 2008;**62**(7):856–65.

30. (X) Idjradinata 1993
* Idjradinata P, Pollitt E. Reversal of developmental delays in iron-deficient anaemic infants treated with iron. *Lancet* 1993;**341**(8836):1–4.
Idjradinata P, Watkins WE, Pollitt E. Adverse effect of iron supplementation on weight gain of iron-replete young children. *Lancet* 1994;**343**(8908):1252–4.

31. (X) Kapur 2003
* Kapur D, Agarwal KN, Sharma S, Kela K, Kaur I. Iron status of children aged 9-36 months in an urban slum integrated child development services project in Delhi. *Indian Pediatrics* 2002;**39**(2):136–44.
Kapur D, Sharma S, Agarwal KN. Dietary intake and growth pattern of children 9-36 months of age in an urban slum in Delhi. *Indian Pediatrics* 2005;**42**(4):351–6.
Kapur D, Sharma S, Agarwal KN. Effectiveness of nutrition education, iron supplementation or both on iron status in children. *Indian Pediatrics* 2003;**40**(12):1131–44.

32. (X) Kashyap 1987
* Kashyap P, Gopaldas T. Hematinic supplementation and hematological status of underprivileged school girls (8-15 years of age). *Nutrition Research* 1987;**7**(11):1127–38.
Seshadri S, Gopaldas T. Impact of iron supplementation on cognitive functions in preschool and school-aged children: the Indian experience. *The American Journal of Clinical Nutrition* 1989;**50 Suppl 3**:675–86.

33. (X) Kianfar 1999

Kianfar H, Kimiagar M, Ghaffarpour M. Effect of daily and intermittent iron supplementation on iron status of high school girls. *International Journal for Vitamin and Nutrition Research* 2000;**70**(4):172–7.

34. (X) Latham 1990
Latham MC, Stephenson LS, Kinoti SN, Zaman MS, Kurz KM. Improvements in growth following iron supplementation in young Kenyan school children. *Nutrition* 1990;**6**(2):159–65.

35. (X) Lawless 1994
Lawless JW, Latham MC, Stephenson LS, Kinoti SN, Pertet AM. Iron supplementation improves appetite and growth in anemic Kenyan primary school children. *The Journal of Nutrition* 1994;**124**(5):645–54.

36. (X) Leenstra 2009
Leenstra T, Kariuki SK, Kurtis JD, Oloo AJ, Kager PA, Ter Kuile FO. The effect of weekly iron and vitamin A supplementation on hemoglobin levels and iron status in adolescent schoolgirls in western Kenya. *European Journal of Clinical Nutrition* 2009;**63**(2):173–82.

37. (X) Lind 2004
* Lind T, Lonnerdal B, Stenlund H, Gamayanti IL, Ismail D, Seswandhana R, et al.A community-based randomized controlled trial of iron and zinc supplementation in Indonesian infants: effects on growth and development. *The American Journal of Clinical Nutrition* 2004;**80**(3): 729–36.
Lind T, Lönnerdal B, Stenlund H, Ismail D, Seswandhana R, Ekström EC, et al.A community-based randomized controlled trial of iron and zinc supplementation in Indonesian infants: interactions between iron and zinc. *The American Journal of Clinical Nutrition* 2003;**77**(4): 883–90. Lind T, Seswandhana R, Persson LA, Lonnerdal B. Iron supplementation of iron-replete Indonesian infants is associated with reduced weight-for-age. *Acta Paediatrica* 2008;**97**(6):770–5.

38. (X) Majumdar 2003
Majumdar I, Paul P, Talib VH, Ranga S. The effect of iron therapy on the growth of iron-replete and iron-deplete children. *Journal of Tropical Pediatrics* 2003;**49**(2):84–8.

39. (X) Massaga 2003
Massaga JJ, Kitua AY, Lemnge MM, Akida JA, Malle LN, Rønn AM, et al.Effect of intermittent treatment with amodiaquine on anaemia and malarial fevers in infants in Tanzania: a randomised placebo-controlled trial. *Lancet* 2003;**361**(9372):1853–60.

40. (X) Mebrahtu 2004 (C)
* Mebrahtu T, Stoltzfus RJ, Chwaya HM, Jape JK, Savioli L, Montresor A, et al.Low-dose daily iron supplementation for 12 months does not increase the prevalence of malarial infection or density of parasites in young Zanzibari children. *The Journal of Nutrition* 2004;**134**(11):3037–41.
Rice AL, Stoltzfus RJ, Tielsch JM, Savioli L, Montresor A, Albonico M, et al.Iron supplementation and mebendazole treatment do not affect respiratory or diarrhoeal morbidity incidence rates in Tanzanian preschoolers. Cited in: Gera 2002 (Gera T, Sachdev HP. Effect of iron supplementation on incidence of infectious illness in children: systematic review. BMJ 2002;325(7373):1142) 1999. [: Gera 2002 (additional references)]
Stoltzfus RJ, Chway HM, Montresor A, Tielsch JM, Jape JK, Albonico M, et al.Low dose daily iron supplementation improves iron status and appetite but not anemia, whereas quarterly anthelminthic treatment improves growth, appetite and anemia in Zanzibari preschool children. *The Journal of Nutrition* 2004;**134**(2):348–56.

Stoltzfus RJ, Kvalsvig JD, Chwaya HM, Montresor
A, Albonico M, Tielsch JM, et al.Effects of iron supplementation and anthelmintic treatment on motor and language development of preschool children in Zanzibar: double blind, placebo controlled study. *BMJ* 2001;**323** (7326):1389–93.

41. **(X) Mejia 1988**
Mejia LA, Chew F. Hematological effect of supplementing anemic children with vitamin A alone and in combination with iron. *The American Journal of Clinical Nutrition* 1988; **48**(3): 595–600.

42. **(X) Menendez 1997**
Beck HP, Felger I, Vounatsou P, Hirt R, Tanner M, Alonso P, et al.Effect of iron supplementation and malaria prophylaxis in infants on *Plasmodium falciparum* genotypes and multiplicity of infection. *Transactions of the Royal Society of Tropical Medicine and Hygiene* 1999;**93 Suppl 1**:41–5.
* Menendez C, Kahigwa E, Hirt R, Vounatsou P, Aponte JJ, Font F, et al.Randomised placebo-controlled trial of iron supplementation and malaria chemoprophylaxis for prevention of severe anaemia and malaria in Tanzanian infants. *Lancet* 1997;**350**(9081):844–50.
Menendez C, Schellenberg D, Quinto L, Kahigwa E, Alvarez L, Aponte JJ, et al.The effects of short-term iron supplementation on iron status in infants in malaria- endemic areas. *American Journal of Tropical Medicine and Hygiene* 2004;**71**(4):434–40.
Menendez C, Sunyer J, Ventura PJ, Aponte JJ, Acosta CJ, Schellenberg D, et al.Malaria infection does not appear to modify the risk of bronchiolitis early in life. *Pediatric Infectious Disease Journal* 2002;**21**(3):249–54.

43. **(X) Mwanri 2000**
Mwanri L, Worsley A, Ryan P, Masika J. Supplemental vitamin A improves anemia and growth in anemic school children in Tanzania. *The Journal of Nutrition* 2000;**130** (11):2691–6.

44. **(X) Nagpal 2004**
* Nagpal J, Sachdev HP, Singh T, Mallika V. A randomized placebo controlled trial of iron supplementation in breast fed young infants initiated on complementary feeding. Effects of haematological status. *Journal of Health, Population, and Nutrition* 2004;**22**(2):203–11.
Nagpal J, Sachdev HPS, Mallika V, Singh T. Iron supplementation with complementary feeding in predominantly breastfed infants. Cited in: Gera 2002 (Gera T, Sachdev HP. Effect of iron supplementation on incidence of infectious illness in children: systematic review. BMJ 2002;325(7373):1142) 2000. [: Gera 2002 (Additional references)]

45. **Nwanyanwu 1996**
Kazembe PN, Nwanyanwu OC, Ziba C, Gamadzi
D, Redd SC. Does iron therapy enhance hematologic recovery in children treated for malaria with sulfadoxine- pyrimethamine. *American Journal of Tropical Medicine and Hygiene* 1995;**53 Suppl 2**:91.
* Nwanyanwu OC, Ziba C, Kazembe PN, Gamadzi G, Gandwe J, Redd SC. The effect of oral iron therapy during treatment for *Plasmodium falciparum* malaria with sulphadoxine-pyrimethamine on Malawian children under 5 years of age. *Annals of Tropical Medicine and Parasitology* 1996;**90**(6):589–95.

46. **(X) Olsen 2006**
Olsen A, Nawiri J, Magnussen P, Krarup H, Friis H. Failure of twice-weekly iron supplementation to increase blood haemoglobin and serum ferritin concentrations: results of a randomized controlled trial. *Annals of Tropical Medicine and Parasitology* 2006;**100**(3):251–

63.

47. (X) Palupi 1997
Palupi L, Schultink W, Achadi E, Gross R. Effective community intervention to improve hemoglobin status in preschoolers receiving once-weekly iron supplementation. *The American Journal of Clinical Nutrition* 1997;**65**(4): 1057–61.

48. (X) Powers 1983
Powers HJ, Bates CJ, Prentice AM, Lamb WH, Jepson M, Bowman H. The relative effectiveness of iron and iron with riboflavin in correcting a microcytic anaemia in men and children in rural Gambia. *Human Nutrition. Clinical Nutrition* 1983;**37**(6):413–25.

49. (X) Richard 2006
Richard SA, Zavaleta N, Caulfield LE, Black RE, Witzig RS, Shankar AH. Zinc and iron supplementation and malaria, diarrhea, and respiratory infections in children in the Peruvian Amazon. *American Journal of Tropical Medicine and Hygiene* 2006;**75**(1):126–32.

50. (X) Rosado 1997
Allen LH, Rosado JL, Casterline JE, López P, Muñoz E, Garcia OP, et al.Lack of hemoglobin response to iron supplementation in anemic Mexican preschoolers with multiple micronutrient deficiencies. *The American Journal of Clinical Nutrition* 2000;**71**(6):1485–94.
* Rosado JL, Lopez P, Munoz E, Martinez H, Allen LH. Zinc supplementation reduced morbidity, but neither zinc nor iron supplementation affected growth or body composition of Mexican preschoolers. *The American Journal of Clinical Nutrition* 1997;**65**(1):13–9.

51. (X) Roschnik 2003 (C) ***{unpublished data only}***
* Roschnik N, Phiri V, Mukaka M. The impact of weekly school-based iron supplementation, Mangochi district, Malawi. Save the Children USA February 2003.

52. (X) Roschnik 2004 (C) Roschnik N, Parawan A, Baylon MA, Chua T, Hall A. Weekly iron supplements given by teachers sustain
the haemoglobin concentration of schoolchildren in the Philippines. *Tropical Medicine & International Health* 2004; **9**(8):904–9.

53. Sarma 1977 (C)
Sarma KV, Damodaran M, Naidu AN. The Balwadi as an outlet for anaemia prophylaxis programme. *Indian Journal of Medical Research* 1977;**65**(6):839–44.

54. (X) Sazawal 2006 (C)a
Olney DK, Pollitt E, Kariger PK, Khalfan SS, Ali NS, Tielsch JM, et al.Combined iron and folic acid supplementation with or without zinc reduces time to walking unassisted among Zanzibari infants 5- to 11-mo old. *The Journal of Nutrition* 2006;**136**(9):2427–34.
* Sazawal S, Black RE, Ramsan M, Chwaya HM, Stoltzfus RJ, Dutta A, et al.Effects of routine prophylactic supplementation with iron and folic acid on admission to hospital and mortality in preschool children in a high malaria transmission setting: community-based, randomized, placebo-controlled trial. *Lancet* 2006;**367** (9505):133–43.

55. (X) Sazawal 2006 (C)b Olney DK, Pollitt E, Kariger PK, Khalfan SS, Ali NS, Tielsch JM, et al.Combined iron and folic acid supplementation with or without zinc reduces time to walking unassisted among Zanzibari infants 5- to 11-mo old. *The Journal of Nutrition* 2006;**136**(9): 2427–34.
* Sazawal S, Black RE, Ramsan M, Chwaya HM, Stoltzfus RJ, Dutta A, et al.Effects of routine prophylactic supplementation with iron and folic acid on admission to hospital and mortality in preschool children in a high malaria transmission setting: community-based,

randomized, placebo-controlled trial. *Lancet* 2006;**367** (9505):133–43.

56. **(X) Seshadri 1982b**
* Seshadri S, Hirode K, Naik P, Malhotra S. Behavior responses of young anemic Indian children to iron-folic supplements. *British Journal of Nutrition* 1982;**48**(2): 233–40.
Seshadri S, Malhotra S. The effect of hematinics on the physical work capacity in anemics. *Indian Pediatrics* 1984; **21**(7):529–33.

57. **Seshadri 1984a**
Seshadri S, Hirode K, Naik P, Shah A, Gupta N. An effective intervention to reduce the prevalence of anaemia in children. *Indian Journal of Medical Research* 1984;**80**: 164–73.

58. **[same reference as above, excluded from count] Seshadri 1984b (C)**
Seshadri S, Hirode K, Naik P, Shah A, Gupta N. An effective intervention to reduce the prevalence of anaemia in children. *Indian Journal of Medical Research* 1984;**80**: 164–73.

59. **(X) Shah 2002**
Shah BK, Gupta P. Weekly vs. daily iron and folic acid supplementation in adolescent Nepalese girls. *Archives of Pediatrics & Adolescent Medicine* 2002;**156**(2):131–5.

60. **Smith 1989 (C)**
* Smith AW, Hendrickse RG, Harrison C, Hayes RJ, Greenwood BM. Iron-deficiency anaemia and its response to oral iron: report of a study in rural Gambian children treated at home by their mothers. *Annals of Tropical Paediatrics* 1989;**9**(1):6–16.
Smith AW, Hendrickse RG, Harrison C, Hayes RJ, Greenwood BM. The effects on malaria of treatment of iron-deficiency anaemia with oral iron in Gambian children. *Annals of Tropical Paediatrics* 1989;**9**(1):17–23.

61. **(X) Smuts 2005**
Hop le T, Berger J. Multiple micronutrient supplementation improves anemia, micronutrient nutrient status, and growth of Vietnamese infants: double-blind, randomized, placebo-controlled trial. *The Journal of Nutrition* 2005;**135**(3): 660S–5S.
* Smuts CM, Lombard CJ, Benadé AJ, Dhansay MA, Berger J, Hop le T, et al.Efficacy of a foodlet-based multiple micronutrient supplement for preventing growth faltering, anemia, and micronutrient deficiency of infants: the four country IRIS trial pooled data analysis. *Journal of Clinical Nutrition* 2005;**135**(3):631S–8S.
Untoro J, Karyadi E, Wibowo L, Erhardt MW, Gross R. Multiple micronutrient supplements improve micronutrient status and anemia but not growth and morbidity of Indonesian infants: a randomized, double-blind, placebo- controlled trial. *The Journal of Nutrition* 2005;**135**(3): 639S–45S.
Wijaya-Erhardt M, Erhardt JG, Untoro J, Karyadi E, Wibowo L, Gross R. Effect of daily or weekly multiple- micronutrient and iron foodlike tablets on body iron stores of Indonesian infants aged 6-12 mo: a double-blind, randomized, placebo-controlled trial. *The American Journal of Clinical Nutrition* 2007;**86**(6):1680–6.

62. **(X) Soekarjo 2004 (C)**
Soekarjo DD, Pee Sd S, Kusin JA, Schreurs WH, Schultink W, Muhilal, et al.Effectiveness of weekly vitamin A (10, 000 IU) and iron (60 mg) supplementation for adolescent boys and girls through schools in rural and urban East Java, Indonesia. *European Journal of Clinical Nutrition* 2004;**58** (6):927–37.

63. **(X) Soemantri 1989**
* Soemantri AG. Preliminary findings on iron supplementation and learning achievement of rural Indonesian children. *The American Journal of Clinical Nutrition* 1989;**50**(3 Suppl):698–

701.

64. (X) Soewondo 1989
Soewondo S, Husaini M, Pollitt E. Effects of iron deficiency on attention and learning processes in preschool children: Bandung, Indonesia. *The American Journal of Clinical Nutrition* 1989;**50**(3 Suppl):667–73.

65. (X) Taylor 2001
Taylor M, Jinabhai CC, Couper I, Kleinschmidt I, Jogessar VB. The effect of different anthelmintic treatment regimens combined with iron supplementation on the nutritional status of schoolchildren in KwaZulu-Natal, South Africa: a randomized controlled trial. *Transactions of the Royal Society of Tropical Medicine and Hygiene* 2001;**95**(2):211–6.

66. (X) van den Hombergh 1996
van den Hombergh J, Dalderop E, Smit Y. Does iron therapy benefit children with severe malaria-associated anaemia? A clinical trial with 12 weeks supplementation of oral iron in young children from the Turiani Division, Tanzania. *Journal of Tropical Pediatrics* 1996;**42**(4):220–7.

67. (X) van Hensbroek 1995
van Hensbroek MB, Morris-Jones, Meisner S, Jaffar S, Bayo L, Dackour R, et al.Iron, but not folic acid, combined with effective antimalarial therapy promotes haematological recovery in African children after acute falciparum malaria. *Transactions of the Royal Society of Tropical Medicine and Hygiene* 1995;**89**(6):672–6.

68. (X) Verhoef 2002 ***{published data only (unpublished sought but not used)}*** Verhoef H, West CE, Kraaijenhagen R, Nzyuko SM, King
R, Mbandi MM, et al.Malarial anemia leads to adequately increased erythropoiesis in asymptomatic Kenyan children. *Blood* 2002;**100**(10):3489–94.
* Verhoef H, West CE, Nzyuko SM, de Vogel S, van der Valk R, Wanga MA, et al.Intermittent administration of iron and sulfadoxine-pyrimethamine to control anaemia in Kenyan children: a randomised controlled trial. *Lancet* 2002;**360**(9337):908–14.

69. (X) Wasantwisut 2006 Wasantwisut E, Winichagoon P, Chitchumroonchokchai C, Yamborisut U, Boonpraderm A, Pongcharoen T, et al.Iron and zinc supplementation improved iron and zinc status, but not physical growth, of apparently healthy, breast-fed infants in rural communities of Northeast Thailand. *The Journal of Nutrition* 2006;**136**(9):2405–11.

70. (X) Zavaleta 2000
Zavaleta N. Efficacy of an intermittent iron dose compared to daily iron supplementation in adolescent girls. 16th International Congress of Nutrition. Canada, 1997:PW5, 62.
* Zavaleta N, Respicio G, Garcia T. Efficacy and acceptability of two iron supplementation schedules in adolescent school girls in Lima, Peru. *The Journal of Nutrition* 2000;**130 Suppl 2S**:462S–4S.

71. (X) Zlotkin 2003
Zlotkin S, Antwi KY, Schauer C, Yeung G. Use of microencapsulated iron(II) fumarate sprinkles to prevent recurrence of anaemia in infants and young children at high risk. *Bulletin of the World Health Organization* 2003;**81**(2): 108–15.

Detailed search strategies

1. iron
2. ferrous
3. IRON COMPOUNDS
4. 1 or 2 or 3
5. supplem*
6. 4 and 5
7. malaria
8. anemia
9. anaemia
10. 7 or 8 or 9
11. 6 and 10
12. child
13. infant*
14. 12 or 13
15. 11 and 14

Length: 186 - 9*3* - *6*4 = 135

((iron OR ferrous OR IRON COMPOUNDS) AND supplem*) AND (malaria OR anemia OR anaemia) AND (child OR infant*)

GOOGLE SCHOLAR SEARCH

(iron OR ferrous) (supplement OR supplementation) (malaria OR anemia OR anaemia) (child OR children OR infant)

Length: 181

searched on 2013-04-10

Hits: 67,800

71 studies included

70 references included

62 references found

11_ipser_pharmacotherapy-anxiety-children

Reference: `Ipser et al. [@the`*`cochrane`*`collaboration`*`pharmacotherapy`*`2009]`

References to studies included in this review

1. **(X) Beidel 2007**
   * Beidel DC, Turner SM, Sallee FR, Ammerman RT, Crosby LA, Pathak S. SET-C versus fluoxetine in the treatment of childhood social phobia. *Journal of the American Academy of Child and Adolescent Psychiatry* 2007;**46**(12):1622–32.

2. **(X) Birmaher 2003**
   * Birmaher B, Axelson DA, Monk K, Kalas C, Clark DB, Ehmann M, Bridge J, Heo J, Brent DA. Fluoxetine for the treatment of childhood anxiety disorders. *Journal of the American Academy of Child and Adolescent Psychiatry* 2003; **42**(4):415–24.

3. **(X) Black 1994**
   * Black B, Uhde TW. Treatment of elective mutism with fluoxetine: A double-blind, placebo-controlled study. *Journal of the American Academy of Child and Adolescent Psychiatry* 1994;**33**(7):1000–6.

4. **(X) DeVeaugh-Geiss 1992**
   * DeVeaugh-Geiss J, Moroz G, Biederman J, Cantwell
   D, Fontaine R, Greist JH, Reichler R, Katz R, Landau P. Clomipramine hydrochloride in childhood and adolescent obsessive-compulsive disorder - a multicenter trial. *Journal of the American Academy of Child and Adolescent Psychiatry* 1992;**31**(1):45–9.

5. **(X) Flament 1985**
   * Flament MF, Rapoport JL, Berg CJ, Sceery W, Kilts C, Mellstrom B, Linnoila M. Clomipramine treatment of childhood obsessive-compulsive disorder. *Archives of General Psychiatry* 1985;**42**:977–83.

6. **(X) Geller 2001a**
   * Geller DA, Hoog SL, Heiligenstein JH, Ricardi Rk, Tamura R, Kluszynski S, Jacobson JG, FPOCDST. Fluoxetine treatment for obsessive-compulsive disorder in children and adolescents: A placebo-controlled clinical trial. *Journal of the American Academy of Child and Adolescent Psychiatry* 2001;**40**(7):773–9.

7. **(X) Geller 2001b**
   * Geller DA, Biederman J, Stewart SE, Mullin B, Farrell C, Wagner KD, Emslie G, Carpenter D. Impact of comorbidity on treatment response to paroxetine in pediatric obsessive-compulsive disorder: Is the use of exclusion criteria empirically supported in randomized clinical trials?. *Journal of Child and Adolescent Psychopharmacology* 2003;**13**(Suppl 1):S19–29.

8. **(X) Geller 2004**
   * Geller DA, Wagner KD, Emslie G, Murphy T, Carpenter DJ, Wetherhold E, Perera P, Machin A, Gardiner C. Paroxetine treatment in children and adolescents with obsessive-compulsive disorder: A randomized, multicenter, double-blind, placebo-controlled trial. *Journal of the American Academy of Child and Adolescent Psychiatry* 2004; **43**(11):1387–96.

9. **(X) Graae 1994**
   * Graae F, Milner J, Rizzotto L, Klein RG. Clonazepam in childhood anxiety disorders. *Journal of the American Academy of Child and Adolescent Psychiatry* 1994;**33**(3): 372–6.

10. **(X) Liebowitz 2002**
   * Liebowitz MR, Turner SM, Piacentini J, Beidel DC, Clarvit SR, Davies SO, Graae F, Jaffer

M, Lin S, Sallee FR, Schmidt AB, Simpson HB. Fluoxetine in children and adolescents with OCD: A placebo-controlled trial. *Journal of the American Academy of Child and Adolescent Psychiatry* 2002;**41**(12):1431–8.

11. **(X) March 1998**
Cook EH, Wagner KD, March JS, Biederman J, Landau P, Wolkow R, Messig M. Long-term sertraline treatment of children and adolescents with obsessive-compulsive disorder. *J Am Acad Child Adolesc Psychiatry* 2001;**40**(10): 1175–81.
* March JS, Biederman J, Wolkow R, Safferman A, Mardekian J, Cook EH, Cutler NR, Dominguez R, Ferguson J, Muller B, Riesenberg R, Rosenthal M, Sallee FR, Steiner H, Wagner KD. Sertraline in children and adolescents with obsessive-compulsive disorder: A multicenter randomized controlled trial. *Journal of the American Medical Association* 1998;**280**(20):1752–6. Wagner KD, Cook EH, Chung H, Messig M. Remission status after long-term sertraline treatment of pediatric obsessive-compulsive disorder. *Journal of Child and Adolescent Psychopharmacology* 2003;**13**(Suppl. 1):S53–60.

12. **(X) March 2007**
* March JS, Entusah RA, Rynn M, Albano AM, Tourian KA. A randomized controlled trial of venlafaxine ER versus placebo in pediatric social anxiety disorder. *Biological Psychiatry* 2007;**62**(10):1149–54.

13. **(X) POTS 2004**
* The Pediatric OCD Treatment Study (POTS)
team. Cognitive-behavior therapy, sertraline, and their combination for children and adolescents with obsessive- compulsive disorder. *Journal of the American Medical Association* 2004;**292**(16):1969–76.

14. **Rapoport 1980**
* Rapoport J, Elkins R, Mikkelsen E. Clinical controlled trial of chlorimipramine in adolescents with obsessive- compulsive disorder. *Psychopharmacology Bulletin* 1980;**16** (3): 61–3.

15. **(X) Riddle 1992**
* Riddle MA, Scahill L, King RA, Hardin MT, Anderson GM, Ort SI, Smith JC, Leckman JF, Cohen DJ. Double- blind, crossover trial of fluoxetine and placebo in children and adolescents with obsessive-compulsive disorder. *Journal of the American Academy of Child and Adolescent Psychiatry* 1992;**31**(6):1062–9.

16. **(X) Riddle 2001**
* Riddle MA, Reeve EA, Yaryura-Tobias JA, Yang HM, Claghorn JL, Gaffney G, Greist JH, Holland D, McConville BJ, Pigott T, Walkup JT. Fluvoxamine for children and adolescents with obsessive-compulsive disorder: A randomized, controlled, multicenter trial. *Journal of the American Academy of Child and Adolescent Psychiatry* 2001; **40**(2):222–9.

17. **(X) RUPPASG 2001**
Ginsburg GS, Riddle MA, Davies M. Somatic symptoms in children and adolescents with anxiety disorders. *Journal of the American Academy of Child and Adolescent Psychiatry* 2006;**45**(10):1179–87.
* The Research Unit on Pediatric Psychopharmacology Anxiety Study Group. Fluvoxamine for the treatment of anxiety disorders in children and adolescents. *New England Journal of Medicine* 2001;**344**(17):1279–85.
Walkup JT, Labellarte MJ, Riddle MA, Pine D, Greenhill L, Klein R, Davies M, Sweeney M, Fu C, Abikoff H, Hack S, Klee B, McCracken J, Bergman L, Piacentini J, March J, Compton

S, Robinson J, O'Hara T, Baker S, Vitiello B, Ritz L, Roper M, Research Units on Pediatric Psychopharmacology Anxiety Study Group. Searching for moderators and mediators of pharmacological treatment effects in children and adolescents with anxiety disorders. *Journal of the American Academy of Child and Adolescent Psychiatry* 2003;**42**(1):13–21.

18. (X) Rynn 2001
* Rynn MA, Siqueland L, Rickels K. Placebo-controlled trial of sertraline in the treatment of children with generalized anxiety disorder. *American Journal of Psychiatry* 2001;**158** (12): 2008–14.

19. (X) Rynn 2007
Kunz NR, Khan A, Lamm LW, Nicolacopoulos E, Jenkins L. Efficacy and safety of venlafaxine extended release in children and adolescents with generalised anxiety disorder. *European Neuropsychopharmacology* 2002;**12**(3):358.
* Rynn MA, Riddle MA, Yeung PP, Kunz NR. Efficacy and safety of extended-release venlafaxine in the treatment of generalized anxiety disorder in children and adolescents: Two placebo-controlled trials. *The American Journal of Psychiatry* 2007;**164**(2):290–300.

20. (X) Simeon 1992
* Simeon JG, Ferguson B, Knott V, Roberts N, Gauthier B, Dubois C, Wiggins D. Clinical, cognitive, and neurophysiological effects of alprazolam in children and adolescents with overanxious and avoidant disorders. *Journal of the American Academy of Child and Adolescent Psychiatry* 1992;**31**(1):29–33.

21. (X) Wagner 2004
* Wagner KD, Berard R, Stein MB, Wetherhold E, Carpenter DJ, Perera P, Gee M, Davy K, Machin A. A multicenter, randomized, double-blind, placebo-controlled trial of paroxetine in children and adolescents with social anxiety disorder. *Archives of General Psychiatry* 2004;**61**: 1153–62.

22. (X) Walkup 2008
* Walkup JT, Albano AM, Piacentini J, Birmaher B, Compton SN, Sherrill JT, Ginsburg GS, Rynn MA, McCracken J, Waslick B, Iyengar S, March JS, Kendall PC. Cognitive behavioral therapy, sertraline, or a combination in childhood anxiety. *New England Journal of Medicine* 2008;**359**(26):2753–66.

Search strategy for electronic databases

PubMed search strategy:

(randomized controlled trial [pt] OR controlled clinical trial [pt] OR randomized controlled trials [mh] OR random allocation [mh] OR double-blind method [mh] OR single-blind method [mh] OR clinical trial[pt] OR clinical trials [mh] OR ("clinical trial" [tw]) OR ((singl* [tw] OR doubl* [tw] OR trebl* [tw] OR tripl* [tw]) AND (mask* [tw] OR blind* [tw])) OR ("latin square" [tw]) OR random* [tw] OR research design [mh:noexp] OR comparative study [pt] OR evaluation studies [pt] OR follow-up studies [mh] OR prospective studies [mh] OR cross-over studies [mh] OR control* [tw] OR prospectiv* [tw] OR volunteer* [tw]) NOT (animal [mh] NOT human [mh]) AND ((placebos [mh] OR placebo* [tw]) AND (anxiety disorder [mh:noexp] OR "anxiety disorder" [tw] OR phobic disorders [mh:noexp] OR obsessive-compulsive disorder [mh] OR "obsessive-compulsive" [tw] OR stress disorders, post- traumatic [mh:noexp] OR "post-traumatic" [tw]) AND ("paediatric" [tw] OR "pediatric" [tw] OR child*[tw] OR child [mh] OR adolesc* [tw] OR

adolescent [mh]))

Length: 1031

GOOGLE SCHOLAR SEARCH

placebo ("anxiety disorder" OR "obsessive-compulsive" OR "post-traumatic") (randomized OR randomised OR random OR controlled OR "double-blind" OR "clinical trial" OR prospective) (child OR children OR adolescent OR pediatric)

Length: 225

searched on 2013-04-10

Hits: 17,700

22 references/studies included

21 references found

12_sultan_flurbiprofen-postoperative-pain

Reference: `Sultan et al. [@the`*cochrane*`collaboration`*single*`2009]`

References to studies included in this review

1. **(X) Boraks 1987**
   Boraks S. Flurbiprofen in low dose compared to dipirona, acido acetilsalicilico and placebo in the treatment of pain post teeth extraction [Flurbiprofen em dose baixa comparado a dipirona, acido acetilsalicilico e placebo
   no tratamento da dor pos–extracao dentaria]. *Arquivos Brasileiros Medicina* 1987;**61**(4):24–30.

2. **(X) Cooper 1986**
   * Cooper SA, Mardirossian G. Comparison of flurbiprofen and aspirin in the relief of postsurgical pain using the dental pain model. *The American Journal of Medicine* 1986;**24;80** (3A):36–40.
   Mardirossian G, Cooper SA. Comparison of the analgesic efficacy of flurbiprofen and aspirin for postsurgical dental pain. *Journal of Oral and Maxillofacial Surgery* 1985;**43**(2): 106–9.

3. **(X) Cooper 1988**
   Cooper SA, Mardirossian G, Milles M. Analgesic relative potency assay comparing flurbiprofen 50, 100, and 150 mg, aspirin 600 mg, and placebo in postsurgical dental pain. *Clinical Journal of Pain* 1988;**4** (1):75–81.

4. **(X) Cooper 1991**
Cooper SA, Kupperman A. The analgesic efficacy of flurbiprofen compared to acetaminophen with codeine. *Journal of Clinical Dentistry* 1991;**2**(3):70–4.

5. **(X) De Lia 1986**
De Lia JE, Rodman KC, Jolles CJ. Comparative efficacy of oral flurbiprofen, intramuscular morphine sulfate, and placebo in the treatment of gynecologic postoperative pain. *American Journal of Medicine* 1986;**80**(3A):60–4.

6. **(X) Dionne 1994**
Dionne RA, Snyder J, Hargreaves KM. Analgesic efficacy of flurbiprofen in comparison with acetaminophen, acetaminophen plus codeine, and placebo after impacted third molar removal. *Journal of Oral and Maxillofacial Surgery* 1994;**52**(9):919–24.

7. **(X) Forbes 1989a**
Forbes JA, Butterworth GA, Burchfield WH, Yorio CC, Selinger LR, Rosenmertz SK, et al. Evaluation of flurbiprofen, acetaminophen, an acetaminophen-codeine combination, and placebo in postoperative oral surgery pain. *Pharmacotherapy* 1989;**9**(5):322–30.

8. **(X) Forbes 1989b**
Forbes JA, Yorio CC, Selinger LR, Rosenmertz SK, Beaver WT. An evaluation of flurbiprofen, aspirin, and placebo in postoperative oral surgery pain. *Pharmacotherapy* 1989;**9** (2):66–73.

9. **(X) Morrison 1986**
Morrison JC, Harris J, Sherrill J, Heilman CJ, Bucovaz ET, Wiser WL. Comparative study of flurbiprofen and morphine for postsurgical gynecologic pain. *American Journal of Medicine* 1986;**80**(3A):55–9.

10. **(X) Sunshine 1983**
Sunshine A, Olson NZ, Laska EM, Zighelboim I, De Castro A, De Sarrazin C. Analgesic effect of graded doses of flurbiprofen in post-episiotomy pain. *Pharmacotherapy* 1983;**3**(3): 177–81.

11. **(X) Sunshine 1986**
Sunshine A, Marrero I, Olson N, McCormick N, Laska EM. Comparative study of flurbiprofen, zomepirac sodium, acetaminophen plus codeine, and acetaminophen for the relief of postsurgical dental pain. *American Journal of Medicine* 1986;**24;80**(3A):50–4.

MEDLINE search strategy (via OVID)

1. Flurbiprofen.sh
2. (flurbiprofen OR Ansaid OR Froben).ti,ab,kw.
3. OR/1-2
4. Pain, postoperative.sh
5. ((postoperative adj4 pain$) or (post-operative adj4 pain$) or post-operative-pain$ or (post$ NEAR pain$) or (postoperative adj4 analgesi$) or (post-operative adj4 analgesi$) or ("post-operative analgesi$")).ti,ab,kw.
6. ((post-surgical adj4 pain$) or ("post surgical" adj4 pain$) or (post-surgery adj4 pain $)).ti,ab,kw.
7. (("pain-relief after surg$") or ("pain following surg$") or ("pain control after")).ti,ab,kw.

8. ((“post surg$” or post-surg$) AND (pain$ or discomfort)).ti,ab,kw.
9. ((pain$ adj4 “after surg$”) or (pain$ adj4 “after operat$”) or (pain$ adj4 “follow$ operat $”) or (pain$ adj4 “follow$ surg$”)).ti,ab,kw.
10. ((analgesi$ adj4 “after surg$”) or (analgesi$ adj4 “after operat$”) or (analgesi$ adj4 “follow$ operat$”) or (analgesi$ adj4 “follow$ surg$”)).ti,ab,kw.
11. OR/4-10
12. randomized controlled trial.pt.
13. controlled clinical trial.pt.
14. randomized.ab.
15. placebo.ab.
16. drug therapy.fs.
17. randomly.ab.
18. trial.ab.
19. groups.ab.
20. OR/12-19
21. humans.sh.
22. 20 AND 21
23. 3 AND 11 AND 22

Length: 1136 - 9*3* - *14*4 = 1053

GOOGLE SCHOLAR SEARCH

(flurbiprofen OR Ansaid OR Froben) pain (randomized OR randomised OR random OR placebo OR trial)

Length: 97

searched on 2013-04-10

Hits: 7950

11 refeerence/studies include

11 references found

13_oduyebo_antimicrobial-vaginosis

Reference: `Oduyebo et al. [@the`*`cochrane`*`collaboration`*`effects`*`2009]`

References to studies included in this review

1. **(X) Andres 1992**
   Andres FJ, Parker R, Hosein I, Benrubi GI. Clindamycin vaginal cream versus oral metronidazole in the treatment of bacterial vaginosis: A prospective double -blind clinical

trial. *Southern Medical Journal* 1992;**85**(11):1077–80.

2. **(X) Anukam 2006a**
Anukam K, Osazuwa E, Ahonkhai I, Ngwu M, Osemene G, Bruce A, Reid G. Augmentation of antimicrobial metronidazole therapy of bacterial vaginosis with oral probiotic Lactobacillus rhamnosus GR-1 and Lactobacillus reuteri RC -14: randomised, double blind, placebo controlled trial. *Microbes and Infection* 2006;**8**(6):1450–4.

3. **(X) Anukam 2006b** AnukamK,OsazuwaE,OsemeneG,AhonkhaiI,Ehigiagbe F, Bruce A, Reid G. Clinical study comparing probiotic Lactobacillus GR-1 and RC -14 with metronidazole vaginal gel to treat symptomatic bacterial vaginosis. *Microbes and Infection* 2006;**8**(12-13): 2772–6.

4. **(X) Beigi 2004**
Beigi RH, Austin MN, Meyn LA, Krohn MA, Hillier SL. Antimicrobial resistance associated with the treatment of bacterial vaginosis. *American Journal of Obstetrics and Gynecology* 2004;**191**:1124–9.

5. **(X) Burana 1990**
Buranawarodomkul P, Chandeying V, Sutthijumroon S. Seven day metronidazole versus single dose tinidazole as therapy for non-specific vaginitis. *Journal of the Medical Association of Thailand* 1990;**73**:283–7.

6. **(X) Chaithong 2003**
* Chaithongwongwatthana S, Limpongsanurak S, Sitthi- Amorn C. Single hydrogen peroxide vaginal douching versus single dose oral metronidazole for the treatment of bacterial vaginosis: a randomised controlled study. *Journal of the Medical Association of Thailand* 2003;**86 Suppl 2**: 379–84.

7. **(X) Eriksson2005** ***{publisheddataonly}***
Eriksson K, Carlsson B, Forsum U, Larsson PG. A double blind treatment study of bacterial vaginosis with normal vaginal lactobacilli after an open treatment with vaginal clindamycin ovule. *Acta Dermato-Venereologica* 2005;**85**: 42–6.

8. **(X) Fischbach 1993**
Fischbaach F, Peterson EE, Weissenbacher ER, Martius J, Hossmann J, Mayer H. Efficacy of clindamycin vaginal cream versus oral metronidazole in the treatment of bacterial vaginosis. *Obstetrics and Gynecology* 1993;**82**:405–10.

9. **(X) Gerli 2003**
Gerli S, Rossetti D, Di Renzo GC. A new approach for the treatment of bacterial vaginosis: use of polyhexamethylene biguanide. A prospective randomised study. *European Review for Medical and Pharmacological Sciences* 2003;**7**: 127–30.

10. **(X) Greaves 1988**
Greaves WL, Chungafung J, Morris B, Haile A, Townsend JL. Clindamycin versus metronidazole in the treatment
of bacterial vaginosis. *Obstetrics and Gynecology* 1988;**72**: 799–802.

11. **(X) Livengood 1990**
Livengood CH, Thomason JL, Hill GB. Bacctterial vaginosis: Treatment with topical intravaginal clindamycin phosphate. *Obstetrics and Gynaecology* 1990;**76**:1.

12. **(X) McCormack 2001**

McCormack WM, Covino JM, Thomason JL, Eschenbach DA, Mou S, Kaperick P, et al.Comparison of clindamycin phosphate vaginal cream with triple sulphonamide vaginal cream in the treatment of bacterial vaginosis. *Sexually Transmitted Diseases* 2001;**28**:569–75.

13. **(X) Milani 2003**
Milani M, Barcellona E, Agnello A. Efficacy of the combination of 2g oral tinidazole and acidic buffering vaginal gel in comparison with vaginal clindamycin alone in bacterial vaginosis: A randomised, investigator blinded, controlled trial. *European Journal of Obstetrics, Gynaecology, and Reproductive Biology* 2003;**109**:62–71.

14. **(X) Nunez 2005**
* Nunez JT, Gomez G. Low-dose secnidazole in the treatment of bacterial vaginosis. *International Journal of Gynaecology and Obstetrics* 2005;**88**:281–5.

15. **(X) Paavonen 2000**
Paavonen J, Mangioni C, Martin MA, Wajszczuk CP. Vaginal clindamycin and oral metronidazole for bacterial vaginosis: A randomised trial. *Obstetrics and Gynecology* 2000;**96**:256–60.

16. **(X) Piot 1983**
Piot P, Van Dyck E, Godts P, Vanderheyden. A placebo- controlled, double blind comparison of tinidazole and triple sulphonamide cream for the treatment of non-specific vaginitis. *American Journal of Obstetrics and Gynecology* 1983;**147**:85–9.

17. **(X) Schinder [this is a spelling error: Author is named Schindler] 1991**
Schinder EM, Thamm H, Ansmann EB, Sarnow E, Schinder AE. Treatment of bacterial vaginosis. *Fortschritte der Medizin* 1991;**109**:138–40.

18. **(X) Schmitt 1992**
Scmitt C, Sobel JD, Meriwhether C. Bacterial vaginosis: Treatment with clindamycin cream versus oral metronidazole. *Obstetrics and Gynecology* 1992;**79**:1020–3.

19. **(X) Schwebke 2000**
Schwebke JR. Asymptomatic bacterial vaginosis: Response to therapy. *American Journal of Obstetrics and Gynecology* 2000;**183**:1434–9.

20. **(X) Schwebke 2006**
* Schwebke JR, Desmond RA. A randomised trial of the duration of therapy with metronidazole plus or minus azithromycin for treatment of symptomatic bacterial vaginosis. *Clinical Infectious Diseases* 2007;**44**:213–9.

21. **(X) Sobel 2001**
Sobel J, Peipert JF, McGregor JA, Livengood C, Matin M, Robbins J, et al.Efficacy of clindamycin vaginal ovule (3 day) treatment versus clindamycin vaginal cream 7 day treatment in bacterial vaginosis. *Infectious diseases Obstetrics and Gynecology* 2001;**9**:9–15.

22. **(X) Stein 1993**
Stein GE, Christensen SL, Mummaw NL, Soper DE. Placebo-controlled trrial of intravaginal clindamycin 2% cream for the treatment of bacterial vaginosis. *The Annals of Pharmacotherapy* 1993;**27**:1343–5.

23. **(X) Voorspoels 2002**
Voorspoels J, Casteels M, Renion JP, Temmerman M. Local treatment of bacterial vaginosis with a bioadhesive metronidazole tablet. *European Journal of Obstetrics, Gynecology, and*

Reproductive Biology 2002;**105**:64–6.

24. **(X) Wathne 1989**
Wathne B, Hovelius B, Holst E. Cefadroxil as alternative to metronidazole in the treatment of bacterial vaginosis. *Scandinavian Journal of Infectious Diseases* 1989;**21**:585–6.

Detailed search strategies

#1	(BACTERIAL VAGINOSIS) OR (VAGINOSIS BACTERIAL) OR (BACTERIAL VAGINITIS)
#2	(BACTERIAL INFECTION) OR (BACTERIAL INFECTIONS)
#3	VAGINITIS OR VAGINOSIS
#4	#2 AND #3

#5 (NONSPECIFIC VAGINITIS) OR (NON-SPECIFIC VAGINITIS) OR (NONSPECI

#6 #1 OR #4 OR #5

#7 “ANTIMICROBIAL THERAPY” OR “ANTIMICROBIAL TREATMENT”

#8 “ANTIMICROBIAL AGENTS” OR ANTIMICROBIAL* OR ANTIBIOTIC OR ANTIBIOTICS

#9 TREATMENT OR THERAPY

#10 #8 AND #9

#11 #7 OR #10

#12 RANDOMIZED CONTROLLED TRIAL[PT] OR CONTROLLED CLINICAL TRIAL[PT] OR RANDOMIZED CONTROLLED TRIALS[MH] OR CONTROLLED CLINICAL TRIALS[MH]

#13 DOUBLE-BLIND METHOD[MH] OR SINGLE-BLIND METHOD[MH] OR CLINICAL TRIALS[MH] OR “CLIN- ICAL TRIAL”

#14 ((SINGL* OR DOUBL* OR TRIPL* OR TREBL*) AND (MASK* OR BLIND*))

#15 PLACEBO[TW] OR PLACEBOS[MH] OR RE DESIGN[MH:NOEXP]

#16 CONTROL[TW] OR PROSPECTIVE*[TW] OR RANDOM*[TW] OR VOLUNTEER*[TW]

#17 #12OR#13OR#14OR#15OR#16

#18 (ANIMALS[TW] OR ANIMAL[MH]) NOT HUMAN[MH]

#19 #17 NOT #18

#20 #6 AND #11 AND #19

#21 PREGNANCY[MH]

Length: 1076 - 45 -53 - 124 - 182 = 957

GOOGLE SCHOLAR SEARCH

(bacterial OR nonspecific OR "non-specific") (vaginitis OR vaginosis) (treatment OR therapy) (randomized OR randomised OR random OR placebo OR trial)

Length: 150

searched on 2013-04-10

Hits: 18,700

24 references/studies studies

24 references found

14_mestre_treatment-huntingtons

Reference: `Mestre et al. [@the`*`cochrane`*`collaboration`*`therapeutic`*`2009]`

References to studies included in this review

1. **(X) Como PG**
   Como PG, Rubin AJ, O'Brien CF, Lawler K, Hickey C, Rubin AE, et al.A controlled trial of fluoxetine in nondepressed patients with Huntington's disease. *Mov Disord 1997;12(3): 397-401* 1997;**12**(3):397–401.

2. **(X) Consroe P**
   Consroe P, Laguna J, Allender J, Snider S, Stern L, Sandyk R, et al.Controlled clinical trial of cannabidiol in Huntington's disease. *Pharmacol Biochem Behav* 1991;**40** (3):701–8.

3. **(X) Cubo E**
[*] Cubo E, Shannon KM, Tracy D, Jaglin JA, Bernard BA, Wuu J, Leurgans SE. Effect of donepezil on motor and cognitive function in Huntington disease. *Neurology* 2006;**10**(7): 1268–71. [DOI: 10.1212/ 01.wnl.0000238106.10423.00]

4. **(X) De Roover J**
Deroover J, Baro F, Bourguignon RP, Smets P. Tiapride versus placebo: a double-blind comparative study in the management of Huntington's chorea. *Curr Med Res Opin* 1984;**9**(5): 329–38.

5. **Goetz CG**
Goetz CG, Tanner CM, Cohen JA, Thelen JA, Carroll VS, Klawans HL, et al. L-acetyl-carnitine in Huntington's disease: double-blind placebo controlled crossover study of drug effects on movement disorder and dementia. *Mov Disord* 1990;**5**(3):263–5.

6. **(X) Hersch G**
Hersch SM, Gevorkian S, Marder K, Moskowitz C, Feigin A, Cox M, et al.Creatine in Huntington disease is safe, tolerable, bioavailable in brain and reduces serum 8OH2'dG. *Neurology* 2006;**66**(2):250–2.

7. **INTRO-HD**
The Huntington Study Group. Safety and tolerability of the free-radical scavenger OPC-14117 in Huntington's disease.. *Neurology* 1998;**50**:1366–1372.

8. **(X) Kieburtz K**
Kieburtz K, Feigin A, McDermott M, Como P, Abwender D, Zimmerman C, et al.A controlled trial of remacemide hydrochloride in Huntington's disease. *Mov Disord* 1996; **11**(3):273–7.

9. **(X) Mateo D**
Mateo D, Gimenez-Roldan S. The effect of piracetam on involuntary movements in Huntington's disease. A double- blind, placebo-controlled study [El efecto del piracetam en los movimientos involuntarios en la enfermedad de Huntington]. *Neurologia* 1996;**11**(1):16–9.

10. **MINO**
The Huntington study group. Minocycline safety and tolerability in Huntington disease. *Neurology* 2004;**63**(3): 547–9.

11. **(X) Murman DL**
Murman DL, Giordani B, Mellow AM, Johanns JR, Little RJ, Hariharan M, et al.Cognitive, behavioral, and motor effects of the NMDA antagonist ketamine in Huntington's disease. *Neurology* 1997;**49**(1):153–61.

12. **(X) O'Suilleabhain P** O'Suilleabhain P, Dewey RB, Jr. A randomized trial of amantadine in Huntington disease. *Arch Neurol* 2003;**60** (7):996–8.

13. **(X) Quinn N**
Quinn N, Marsden CD. A double blind trial of sulpiride in Huntington's disease and tardive dyskinesia. *J Neurol Neurosurg Psychiatry* 1984;**47**(8):844–7.

14. **RID-HD**
The Huntington study group. Dosage effects of riluzole in Huntington's disease: a multicenter

placebo-controlled study. *Neurology* 2003;**61**(11):1551–6..

15. **(X) Roos RA**
Roos RA, Buruma OJ, Bruyn GW, Kemp B, van der Velde EA. Tiapride in the treatment of Huntington's chorea. *Acta Neurol Scand* 1982;**65**(1):45–50.

16. **(X) Stocchi F**
Stocchi F, Carta A, Berardelli A, Antonini A, Argenta M, Formica A, et al.Effects of terguride in patients with Huntington's disease. *Clin Neuropharmacol* 1989;**12**(5): 435–9.

17. **(X) TETRA-HD**
The Huntington study group. Tetrabenazine as antichorea therapy in Huntington disease: a randomized controlled trial. *Neurology* 2006;**66**(3):366–72.

18. **TREND-HD**
The Huntington Study group. TREND-HD - A Trial of Ethyl-EPA (MiraxionTM) in Treating Mild to Moderate Huntington's Disease. World Congress in Huntington´ s disease. 2007.

19. **(X) Vaddadi K**
Vaddadi KS, Soosai E, Chiu E, Dingjan P. A randomised, placebo-controlled, double blind study of treatment
of Huntington's disease with unsaturated fatty acids. *Neuroreport* 2002;**13**(1):29–33.

20. **(X) van Vugt J**
van Vugt JP, Siesling S, Vergeer M, van der Velde EA, Roos RA. Clozapine versus placebo in Huntington's disease: a double blind randomised comparative study. *J Neurol Neurosurg Psychiatry* 1997;**63**(1):35–9.

21. **(X) Verhagen Metman L** Verhagen Metman L, Morris MJ, Farmer C, Gillespie M, Mosby K, Wuu J, et al 12. Huntington's disease: a randomized, controlled trial using the NMDA-antagonist amantadine. *Neurology* 2002;**59**(5):694–92.

Search methods for identification of studies

For MEDLINE and Cochrane Controlled Trials Register, we used the following MeSH search strategy:

1. huntington/all subheadings
2. chorea/all subheadings
3. drug therapy
4. prevention and control
5. rehabilitation
6. surgery
7. therapy
8. psychology
9. mortality
10. #1,2 AND #3
11. #1,2 AND #4
12. #1,2 AND #5
13. #1,2 AND #6
14. #1,2 AND #7
15. #1,2 AND #8

16. #1,2 AND #9
17. 10-16
18. #17 AND limit: clinical trial
19. in humans

Length: 334 - 9*3* - *10*4 = 267

GOOGLE SCHOLAR SEARCH

("huntington's disease" OR "huntington disease" OR "huntington chorea" OR "huntington's chorea") (therapy OR treatment OR surgery OR prevention OR prophylaxis OR psychology) (randomized OR randomised OR random OR placebo OR trial)

Length: 229

searched on 2013-04-10

Hits: 17,200

21 references/studies included

16 references found
