# Supplementary material for: Google Scholar as replacement for systematic literature searches: good relative recall and precision are not enough
Source: BMC Med Res Methodol. 2013 Oct 26;13:131. doi: 10.1186/1471-2288-13-131 (PMC3840556; doi:10.1186/1471-2288-13-131)
Supplement: Additional file 2 — Detailed information on the original precision of the searches for the Cochrane reviews. Data retrieved from the Cochrane reviews. Presented are: Number of databases searched and type of additional resources, number of references found with or without doublets where available, number of references included in the Cochrane review, and the calculated precision from the former. If a reference was cited for more than one study it was counted only once in the column “references included”. [file 1471-2288-13-131-S2.pdf]

Review	No.l DBs	references found (w or w/o doublets unknown)		references included	precision %
		w doublets	w/o doublets		
1 arroll	2 (?) controlled trials register: strategy available<br>X inconsistent about inclusion of additional 6 DBs	--	--	14	--
2 sinclair	5 (+1 study register)	**517**		49	9.4
3 mcqueen<br>new Issue 2011	5 (+1 study register)		**636**<br>614 (DBs) +<br>22 other	14 (in older Issue only 11)	2.2
4 bar-on<br>new Issue 2012	3 (+1 study register)	**246**		20	8.1
5 bohlius	3<br>(+ conference proceedings + ESA trials)	**5546**<br>(+ ongoing: 575)	--	**48**<br>(Gehanno only 39 due to excluded abstracts)	0.9
6 boehm	7	**675**		**51**	7.6
7 guimaraes	5	**775**		**11**	1.4
8 verbeek<br>new Issue 2012	8 or 14<br>(+ 1 study register). Inconsistent listing	**2491**<br>references (1360 in 2009 plus 1129 in 2012), of which 1198 came from a combined search of MEDLINE and EMBASE using Ovid, 86 from CINAHL, 76 from CENTRAL and nine from the COHF database up until 2005. An additional search from 2005 to December 2008 yielded an additional 256 references. The update in January and February 2012 for references from 2009 to 2012 brought 54 new references from PubMed, 299 from EMBASE, 601 from Web of Science, 168 from NIOSHTIC and 7 references from reference lists of articles.		**24**<br>(One article described two trials and two articles described the same study. This resulted in 25)	1.0
9 yip	7	**--**		**17**	--
10 okebe<br>neue Version 2011	5 (+ 2 study registers)	resulting in a large number of publications		**70**	--
11 ipser	4<br>(+ various study registers)	**1395**<br>sum of single DB hits<br>(+ ongoing: 655 + 193)		**22**<br>(Gehanno 25: possible due to inclusion of more than one reference per trial)	1.6
12 sultan	4	**--**	--	**11**	--
13 oduyebo	5 (+ conference proceedings)	**701**		**24**	3.4
14 mestre	3<br>(+ 1 study register + handsearch etc.)	**102**		**21**<br>(Gehanno 20)<br>Only published references, not personal commincation	20.6
